# Novel Trifluoromethyl-Substituted Dipyrimidinamine Derivatives as Potential Antifungal Agents for Controlling Postharvest Soft Rot in Kiwifruit

**DOI:** 10.3390/jof12090645

**Published:** 2026-09-01

**Authors:** Wenneng Wu, Jingyan Zhao, Peng Geng, Qiang Fei, Haijiang Chen

**Affiliations:** School of Food Science and Engineering, Guiyang University, Guiyang 550003, China; zhaojingyann@163.com (J.Z.); gengpeng231012@126.com (P.G.); fqorganic@163.com (Q.F.)

**Keywords:** fungicide, synthesis, dipyrimidinamine derivatives, antifungal activity

## Abstract

Postharvest soft rot poses a severe threat to the kiwifruit industry. In response to this challenge, 20 trifluoromethyl-substituted dipyrimidinamine derivatives were designed and synthesized based on the molecular hybridization strategy. The bioassay results showed that most of the target compounds exhibited good inhibitory activity against plant pathogenic fungi. Among these compounds, compound **6p** demonstrated the most potent activity against the postharvest pathogens *Botryosphaeria dothidea* and *Phomopsis* sp., with EC_50_ values of 8.92 and 13.11 μg/mL, respectively, which were significantly better than the commercial fungicide pyrimethanil. At 200 μg/mL, the protective activity and therapeutic activity of compound **6p** on kiwifruit were better than the commercial control pyrimethanil. Mechanistic evidence suggested that compound **6p** markedly perturbs the structural integrity of the pathogenic fungal membrane, thereby triggering extensive leakage of proteins, nucleic acids, and other cytoplasmic contents, and provoking reactive oxygen species (ROS) accumulation. Molecular docking simulation results indicated strong binding affinity of the compound toward the crystal structure of a homologous cystathionine gamma-synthase. The results of this study provide an important scientific basis and molecular design reference for new trifluoromethylpyrimidine fungicides.

## 1. Introduction

Postharvest plant pathogenic fungal diseases are key factors that lead to serious loss of agricultural products, decline in quality, and threats to food safety [1]. Soft rot caused by *Botryosphaeria dothidea* and *Phomopsis* spp. is one of the most severe postharvest diseases of kiwifruit [2,3]. This disease often leads to extensive fruit rot during storage and causes significant economic losses. At present, its prevention and control are still highly dependent on chemical fungicides such as difenoconazole, azoxystrobin, and fludioxonil [4]. However, long-term and repetitive use of these compounds has led to increasingly prominent resistance of pathogens, as well as problems such as pesticide residues and environmental risks [5,6,7]. For instance, resistance to difenoconazole, azoxystrobin, and fludioxonil has been documented in various plant pathogenic fungi [5]. Moreover, several field strains resistant to pyrimethanil have been isolated [8,9], and some pathogens have acquired multi-fungicide resistance (including pyrimethanil) through efflux pump-mediated mechanisms [10]. In this context, the introduction of new fungicides with distinct structural features is essential not only to provide effective disease control but also to enable rotation strategies that combine agents with different targets and mechanisms, thereby delaying the evolution of resistance in phytopathogens.

**Figure 1:**
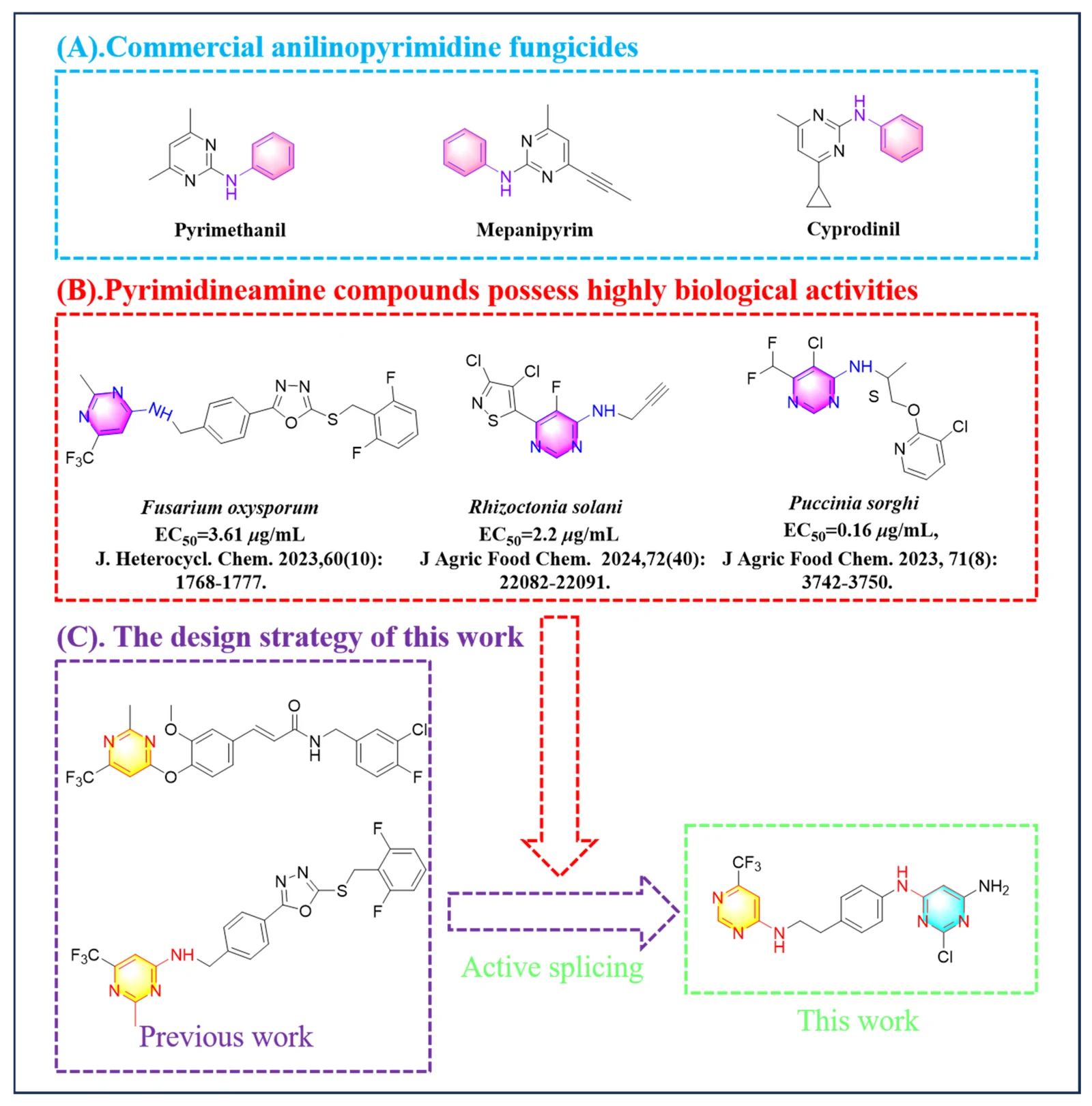
Structural diagram compounds: (**A**) Commercial anilinopyrimidine fungicides. (**B**) Pyrimidineamine compounds possess broad-spectrum biological activities [33,34,39]. (**C**) Design strategy of target compounds.

Pyrimidine, as an aromatic heterocyclic ring with strong structural plasticity, exhibits broad-spectrum biological activities, including herbicidal, antifungal, antiviral, antibacterial, and insecticidal properties [11,12,13,14,15,16]. It is the dominant skeleton for the construction of bioactive molecules. Among them, pyrimidamine compounds have broad-spectrum biological activities, and commercial pesticides such as pyrimethanil, mepanipyrim, cyprodinil (Figure 1A) against plant pathogenic fungi have been developed. They exhibit prominent advantages owing to their unique mechanisms of action and potent fungicidal activity, making them a key research direction for addressing pathogen resistance [17,18]. Based on the primary strategy of active scaffold derivatization for the development of innovative pesticides [19], targeted structural modification of the pyrimidine scaffold is a promising approach for discovering novel compounds with potent activity [20,21]. In addition, the trifluoromethyl group is the core fluorine-containing functional group in the molecular design of modern pesticides. It possesses unique physicochemical properties and the ability to modulate biological activity and is widely utilized in the development of insecticides, fungicides, and herbicides [22,23,24]. Its strong electron-withdrawing effect can precisely modulate the electron cloud distribution of the molecule, enhancing hydrogen bonding and hydrophobic interactions with the target protein, thereby significantly improving binding affinity and activity; core varieties such as fipronil and flonicamid rely heavily on this feature [22,25,26,27]. The high lipophilicity of trifluoromethyl group improves the transmembrane permeability and systemic translocation of pesticides, which facilitates crop absorption and translocation to target sites [28]. In recent years, trifluoromethyl group has been incorporated into the design and research of fungicides, such as flufenoxadiazam, cyprometoxazam and fluopimomide [29,30]. Therefore, the trifluoromethyl group is a key fragment to achieve high activity, stable metabolism, strong penetration and excellent selection, serving as a privileged motif for the development of novel agrochemicals.

Based on the above analysis, the introduction of a trifluoromethyl group into the pyrimidine skeleton can significantly improve the lipid solubility, metabolic stability, and binding ability of the molecule to its target, thereby enhancing its biological activity [31,32]. Introducing an aromatic amine linker on the pyrimidine skeleton has proven to be another key modification [33,34]. In addition, our previous studies on various trifluoromethylpyrimidine derivatives clearly confirmed that the introduction of trifluoromethyl group can greatly boost the antifungal potency of pyrimidine derivatives [35,36,37,38,39,40,41]. Guided by an active fragment splicing strategy, we introduced a trifluoromethyl group into the pyrimidinamine scaffold to optimize its physicochemical properties. Specifically, the trifluoromethyl group was incorporated into the core pyrimidinamine pharmacophore, and structural diversity was achieved via an aniline linker, to explore the potential activity of the resulting derivatives against phytopathogenic fungi.

Antifungal activity assays revealed that compound **6p** exhibited the most potent inhibitory activity against several key pathogens responsible for kiwifruit soft rot. Subsequent mechanistic studies revealed that **6p** inhibits fungal enzymatic activity, destroys the defense barrier of the mycelium, and promotes endogenous reactive oxygen species (ROS) accumulation, collectively suppressing pathogen growth. In summary, the trifluoromethyl-pyrimidinamine derivatives developed in this study demonstrate promising potential for the environmentally benign management of postharvest soft rot in kiwifruit.

## 2. Materials and Methods

### 2.1. Materials and Instruments

The nuclear magnetic resonance spectrometer was a JEOL-ECX500 (JEOL Ltd., Tokyo, Japan). The high resolution mass spectrometer was an Agilent ESI-MSD Trap VL (Agilent Technologies, Santa Clara, CA, USA). The element analyzer was an Elementar Vario-III (Elementar Analysensysteme GmbH, Langenselbold, Germany). The melting point was measured by an X-4 digital display micro melting point tester (Beijing Tech Instrument Co., Ltd., Beijing, China). All chemical reagents are analytically pure and purchased from commercial suppliers.

### 2.2. Synthesis of the Target Compound

**Figure 2:**
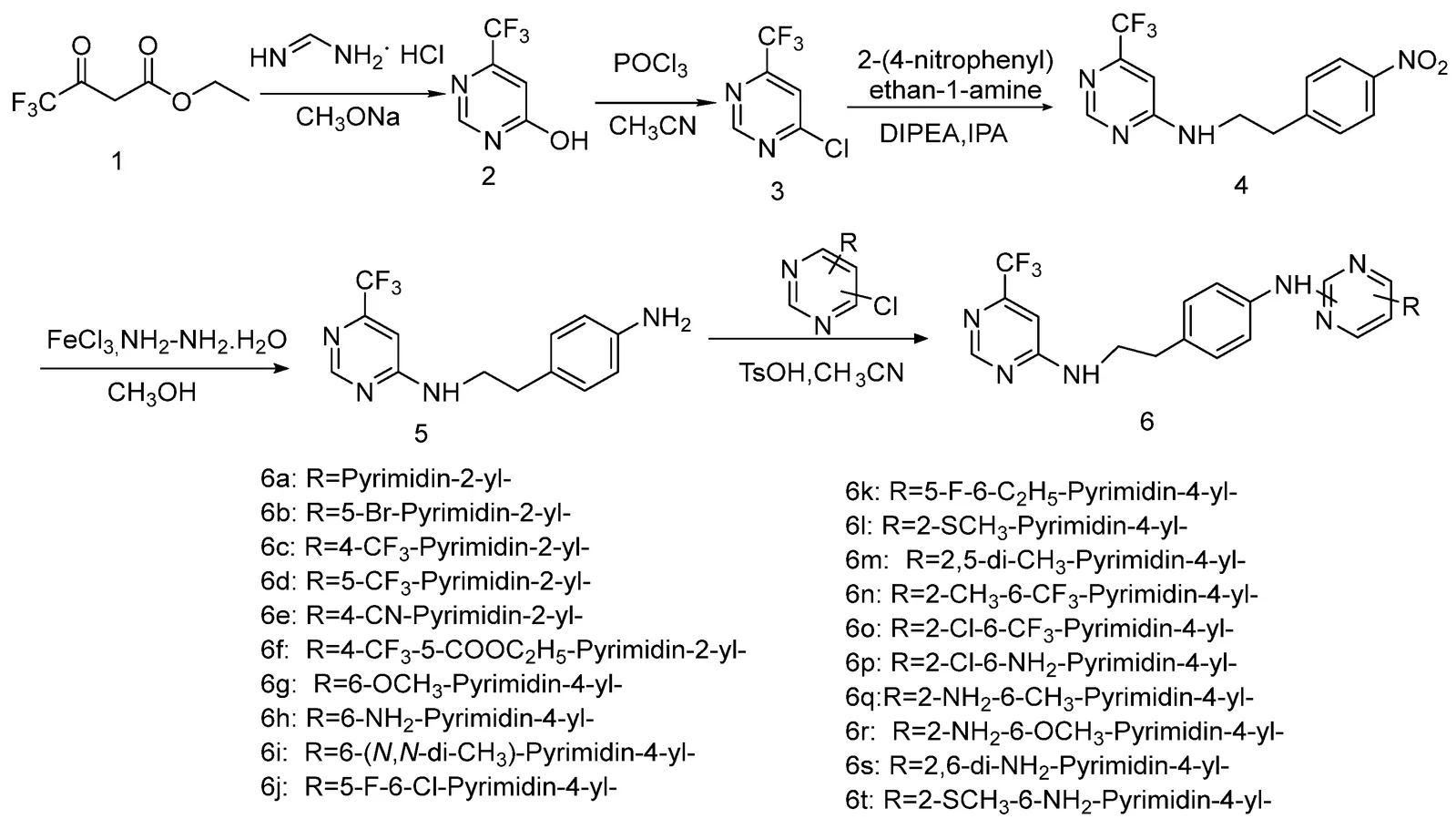
General synthesis route for the target compounds **6a**–**6t**.

Using ethyl trifluoroacetoacetate as the starting material, stepwise reactions sequentially gave intermediates **2**, **3**, **4**, **5**, and finally the target compounds **6a**–**6t**, which is illustrated in Figure 2. The preparation of intermediates **2** and **3** followed the method reported by our research group [38,42]. Then, intermediate **3** was reacted with 4-nitrophenylethylamine hydrochloride to obtain intermediate **4**, which was subjected to alkaline reaction conditions and underwent a reduction reaction to obtain intermediate **5**. The overall synthetic strategy is illustrated in Figure 2.

#### 2.2.1. Synthesis of Intermediate **4**

4-Nitrophenethylamine hydrochloride (19.7 mmol) was taken up in an appropriate amount of isopropanol, followed by the addition of DIPEA (24.2 mmol), and stirred for 30 min until complete dissolution. Intermediate **3** (24.15 mmol) was taken up in isopropanol and then introduced dropwise through a constant-pressure dropping funnel over 30 min. Upon completion of the addition, the reaction mixture was refluxed. TLC monitoring showed the reaction was complete.

Upon completion of the reaction, the solvent was removed under reduced pressure. Three sequential extractions with ethyl acetate were performed on the crude residue. The collected organic fractions were treated with anhydrous sodium sulfate for dehydration, subjected to filtration, and reduced in volume to yield crude intermediate **4**. The crude product solid was recrystallized from ethanol to obtain a yellow solid intermediate **4**.

*N*-(4-nitrophenethyl)-6-(trifluoromethyl)pyrimidin-4-amine (**4**): Yellow solid, yield 84.1%, m.p. 131.6–133.3 °C; ^1^H NMR (600 MHz, DMSO-*d*_6_, ppm) *δ* 8.58 (s, 1H, Pyrimidine-H), 8.16 (d, *J* = 8.43 Hz, 2H, Ph-H), (t, *J* = 5.10 Hz, 1H, -CH_2_NH-), 7.54 (d, *J* = 8.41 Hz, 2H, Ph-H), 6.85 (s, 1H, Pyrimidine-H), 3.68 (q, *J* = 6.72 Hz, 2H, -CH_2_NH-), 3.00 (t, *J* = 7.01 Hz, 2H, -CH_2_-).

#### 2.2.2. Synthesis of Intermediate **5**

Intermediate **4** (16.03 mmol) and ferric chloride (19.37 mmol) were weighed and added with fine activated carbon, poured into anhydrous methanol and stirred. A total of 15 mL of hydrazine hydrate (80%) was slowly dropped into the reaction system through a constant pressure drop funnel. The dropping time was controlled within 30 min. The mixture was then heated to 85 °C after the feed was complete, and TLC was employed to verify the reaction status. After the reaction, the activated carbon and solid impurities were removed by filtration, and the residue was washed twice with methanol. The filtrate was combined, the methanol was removed by vacuum concentration, and 50 mL of deionized water was added to the residue. After stirring, we performed three successive extractions of the residue with ethyl acetate. We added anhydrous sodium sulfate to the mixture for dehydration, then removed the solid by filtration and removed the solvent in vacuo. The residue was recrystallized with ethanol to obtain yellow solid intermediate **5**.

*N*-(4-aminophenethyl)-6-(trifluoromethyl)pyrimidin-4-amine (**5**): Yellow solid, yield 78.8%, m.p. 98.8–101.5 °C; ^1^H NMR (600 MHz, DMSO-*d*_6_, ppm) *δ* 8.57 (s, 1H, pyrimidine-H), 8.05 (t, *J* = 5.40 Hz, 1H, -CH_2_NH-), 6.90 (d, *J* = 8.04 Hz, 2H, Ph-H), 6.86 (s, 1H, pyrimidine-H), 6.51 (d, *J* = 10 Hz, 2H, Ph-H), 4.88 (s, 2H, -NH), 3.53 (q, *J* = 6.60 Hz, 2H, -CH_2_NH-), 2.68 (t, *J* = 7.38 Hz, 2H, -CH_2_-).

#### 2.2.3. Preparation Procedure of the Target Compounds **6a**–**6t**

Acetonitrile (10 mL) was introduced into a flask containing intermediate **5** (2.12 mmol) and p-toluenesulfonic acid (2.1 mmol), and the blend was agitated for 30 min. Then, the appropriate substituted pyrimidine chloride (2.31 mmol) was introduced, and the resulting mixture was heated to 85 °C under reflux. TLC assessment was conducted until the reaction was complete, at which point the volatiles were evaporated in vacuo. The residue was suspended in H_2_O (20 mL) and extracted three times with EtOAc (20 mL each). The collected organic phase was dried (Na_2_SO_4_), filtered, and concentrated under reduced pressure. Purification via silica gel column chromatography gave the title compounds **6a**–**6t**. The physical characteristics, along with the NMR and HRMS spectra for compounds **6a**–**6t** were documented in the Supplementary Materials.

(2-((6-(Trifluoromethyl)pyrimidin-4-yl)amino)ethyl)phenyl)pyrimidin-2-amine (**6a**): white solid; yield 45.4%; m.p. 163.4–165.1 °C; ^1^H NMR (600 MHz, DMSO-*d*_6_) *δ* 9.56 (s, 1H, -NH-), 8.58 (s, 1H, pyrimidine-H), 8.45 (d, *J* = 4.80 Hz, 2H, pyrimidine-H), 8.10 (t, *J* = 5.34 Hz, 1H, -CH_2_NH-), 7.68 (d, *J* = 8.40 Hz, 2H, Ph-H), 7.16 (d, *J* = 8.40 Hz, 2H, Ph-H), 6.87 (s, 1H, pyrimidine-H), 6.81 (t, *J* = 4.74 Hz, 1H, pyrimidine-H), 3.61 (q, *J* = 6.78 Hz, 2H, -CH_2_NH-), 2.81 (t, *J* = 7.26 Hz, 2H, -CH_2_-); ^13^C NMR (150 MHz, DMSO-*d*_6_) δ 162.81, 160.51, 159.78, 158.43, 151.58(q, *J* = 33.60 Hz), 139.14, 132.48, 129.13, 122.45 (q, *J* = 272.51 Hz), 119.48, 112.64, 102.86, 42.26, 34.29; HRMS(ESI)m/z [M+H]^+^ calcd for C_17_H_16_N_6_F_3_: 361.13831, found: 361.13841.

### 2.3. In Vitro Antifungal Activity Test

Strains of *Botryosphaeria dothidea* (BD) and *Phomopsis* spp. (PS) were previously isolated from postharvest kiwifruit suffering soft rot. To assess the broad-spectrum potential of the compounds, the rice blast model strain *Magnaporthe oryzae* Guy11 (MO), and several isolates of *Botrytis cinerea* separated from cucumber (CBC), blueberry (BBC), or tobacco (TBC) were included in the screening. The strains/isolates were regularly maintained in our lab and had been used in other works [35,36,39]. Potato dextrose agar (PDA) medium was supplemented with the test compounds at a final concentration of 50 μg/mL. The compounds were initially solubilized in DMSO and subsequently diluted with sterile water; an equal volume of DMSO was used as the solvent control. After solidification, a mycelial plug was centrally inoculated onto each plate, followed by incubation at 25 °C for 3–4 days. Each treatment was set with three replicates. Colony diameters were measured using the cross method, and the inhibition rate was calculated according to the formula [43,44]:

$$\mathrm{Inhibition}\;\mathrm{rate}\left(\%\right)=\frac{\mathrm{CK}-C_{1}}{\mathrm{CK}-0.5}\times 100\%$$

CK: Mycelial colony diameter of blank control (treated with sterile water); C_1_: Mycelial colony diameter after compound treatment (cm); 0.5: Diameter of inoculated mycelial plug (0.5 cm).

EC_50_ values were determined by probit analysis using SPSS software (version 26.0).

### 2.4. In Vivo Assay Against Kiwifruit Soft Rot

BD was cultured on PDA plates at 25 °C for 5–7 days. Mycelial plugs (0.5 cm in diameter) were excised from the colony margin using a sterile cork borer for fruit inoculation. Kiwifruit of uniform size and free from visible defects were surface-sterilized with 75% ethanol for 30 s, rinsed three times with sterile distilled water, and air-dried. For the protective assay, fruits were sprayed with test compound solutions at concentrations of 100 and 200 μg/mL (containing 0.1% Tween-80) until the entire fruit surface was wet. After 24 h, a wound (approximately 2 mm deep) was made at the equator of each fruit using a sterile needle, and a 0.5 cm mycelial plug of BD was inoculated onto each wound site. For the curative assay, fruits were first inoculated with BD as described above; 24 h after inoculation, the fruits were sprayed with the same concentrations of test compound solutions. Fruits treated with 0.1% Tween-80 and pyrimethanil (at the same concentrations) served as negative and positive controls, respectively. Each treatment consisted of six fruits (*n* = 6), and the entire experiment was performed in triplicate [45,46]. After treatment, all fruits were incubated at 25 °C with 85% relative humidity for 3–4 days. The lesion diameter (cm) of each fruit was measured using the cross method. Protective and curative effects were calculated according to the formula:

$$P\;\mathrm{or}\;C\left(\%\right)=\frac{D_{c}-D_{t}}{D_{c}-1}\times 100\%$$

where P and C are the protective and therapeutic effects, respectively, D_t_ is the lesion diameter in protective or therapeutic group (cm), D_c_ is the lesion diameter in blank control group (cm) and 1 is the fungal stem diameter (cm).

### 2.5. SEM Studies on the Cell Morphology

PDA media amended with compound **6p** and pyrimethanil both at 50 μg/mL, as well as DMSO-only control medium, were prepared. Each treatment was set with three replicates. The medium was poured into three plates, allowed to solidify, and then inoculated with BD mycelial plugs (cut with a sterile borer), followed by incubation at 25 °C for 4–6 days [47].

The cultured hyphae were divided into small sections. Then, they were fixed in 5 mL tubes with 2.5% glutaraldehyde. Once fixed, the specimens were washed three washes with phosphate buffer adjusted to pH 7.0 and subsequently immersed 1% osmium tetroxide for 1–2 h.

The osmium solution was removed. The samples were subjected to three additional washes using a phosphate-based buffer solution. Samples were dried with sequential alcohol concentrations, then desiccated and coated with gold via sputtering before being examined by scanning electron microscopy (SEM).

### 2.6. Determination of Mycelial Dry Weight

For the mycelial growth inhibition assay, a 0.5 g portion of mycelia was inoculated into 30 mL of potato dextrose broth (PDB) medium. Concentrations of Compound **6p** (12.5, 25, and 50 μg/mL) were established in the cultures, respectively. After culture, the medium was removed by centrifugation, and the mycelia were collected by washing with distilled water. The dry weight was measured after lyophilized for 12 h. Each treatment was set with three replicates, and the relative reduction rate of dry weight (R) was calculated using the reported formula [48].

### 2.7. Determination of Mycelial Cell Membrane Permeability

Mycelial plugs were introduced into PDB medium, shaken and centrifuged to remove the medium, and the hyphae were washed with sterile water. Compound **6p** was added to achieve final supplemented at 12.5, 25, and 50 μg/mL, with an equal volume of DMSO serving as the solvent control. The determination was performed at 0, 1, 2, 3, 4, and 5 h (Each treatment was set with three replicates.). Subsequently, the mycelia were boiled for 10 min, cooled, and measured again. Finally, the conductivity was calculated using the formula [49]. The calculation formula is as follows:

$$P=\frac{\left(C_{t}-C_{0}\right)}{\left(C_{k}-C_{0}\right)}\times 100\%$$

In the formula, P represents the relative membrane permeability; C_0_ is the conductivity before treatment; C_t_ is the conductivity of experimental groups at different time points; C_k_ is the conductivity of inactivated mycelial suspension.

### 2.8. Determination of Mycelial Cellular Contents Leakage

Mycelial samples (0.5 g) were placed into 30 mL of PDB medium with compound **6p** at 12.5, 25, and 50 μg/mL. A blank control consisted of equal volume of DMSO. The samples were kept at 25 °C for 24 h. A UV spectrometer was then used to take readings at 260 nm and 280 nm. Each condition was tested in triplicate [48,50].

### 2.9. Determination of Reactive Oxygen Species (ROS)

Pyrimethanil and compound **6p** were separately diluted in PDB medium to obtain series concentrations of 12.5, 25, and 50 μg/mL, and were incubated at 25 °C for 24 h. The mycelia were pelleted by centrifugation and washed with PBS. A total of 0.2 mL 10 mmol/L 2′,7′-dichlorodihydrofluorescein diacetate (DCFH-DA) staining solution was added. This was followed by a 20 min incubation in the dark. After staining, the mycelia were washed with PBS buffer, and the fluorescence was visualized under a fluorescence microscope [51,52].

### 2.10. Determination of Enzyme Activity

A commercial superoxide dismutase (SOD) assay kit was used to investigate the effects of compound **6p** and pyrimethanil on SOD activity. BD was cultured in PDB medium for 48 h before adding compound **6p** and pyrimethanil at 0, 12.5, 25, and 50 μg/mL. The treated cultures were then incubated at 25 °C for another 48 h, and the mycelia were harvested by filtration and freeze-dried. Then we measured the SOD inhibitory activity with the kit, just as the manual says [53].

### 2.11. Molecular Docking Simulation

Compound **6p** and pyrimethanil were selected for molecular docking simulation. Pyrimidinamine fungicides such as pyrimethanil are known to act primarily via inhibition of methionine biosynthesis, in which cystathionine γ-synthase (CGS) plays a key role. Here we directly adopted the crystal structure of CGS from tobacco (PDB ID: 1I48) for molecular docking target in consideration of the following reasons. Although the BD genome is publicly available, its annotation remains incomplete, and the gene models for CGS have not been fully curated. Additionally, searching the UniProt database revealed multiple CGS-like sequences from BD with varying lengths, all showing comparable sequence similarity to the tobacco CGS (1I48), making it difficult to determine which isoform represents the bona fide target. Moreover, these sequences originate from shotgun sequencing assemblies and may contain sequencing errors or assembly artifacts, further complicating accurate homology modeling. Importantly, despite these challenges in constructing a BD homology model, comparison of the CGS crystal structures from bacterial and plant sources confirms that the overall fold and active-site architecture are highly conserved across kingdoms. This structural conservation supports the use of the experimentally determined tobacco CGS as a reliable surrogate for predicting the binding mode of compound **6p**. AutoDock software (version 4.2.6) were used to perform molecular docking after removing water molecules and binding ligands and adding hydrogen atoms to the receptor protein, and PyMOL (version 2.6) was used for visual analysis of the docking conformation [18].

## 3. Results

### 3.1. Synthesis

This synthetic route employs ethyl trifluoroacetoacetate and formamidine hydrochloride as starting materials and involves five consecutive steps: cyclization, chlorination, condensation, reduction, and condensation to construct the target compounds **6a**–**6t**. First, the two starting materials undergo a cycloaddition reaction in the presence of sodium methoxide to afford intermediate **2** in 80.6% yield. Subsequently, intermediate **2** is chlorinated with phosphorus oxychloride to give intermediate **3**, which is then condensed with 4-nitrophenethylamine to provide intermediate **4** in 84.1% yield. Intermediate **4** is then refluxed with hydrazine hydrate and ferric chloride in methanol to yield the key intermediate **5** through reduction, with a yield of 77.8%. Finally, intermediate 5 is condensed with various substituted pyrimidine chlorides to smoothly afford the target compounds **6a**–**6t** in overall yields ranging from 39.5% to 57.8%. The ^1^H NMR, ^13^C NMR, and HRMS data for **6a**–**6t** are provided in the Supplementary Materials.

### 3.2. In Vitro Antifungal Activity

At a concentration of 50 μg/mL, the twenty synthesized compounds (**6a**–**6t**) exhibited inhibition rates of 13.91–70.72% against MO, 11.50–77.88% against PS, 11.34–92.54% against CBC, 10.36–91.72% against BD, 10.75–73.73% against TBC, and 18.60–83.43% against BBC. As detailed in Table 1, compound **6p** was the only one to outperform the reference fungicide pyrimethanil across all six pathogens. At 50 μg/mL, it gave inhibition rates of 70.72% against MO, 77.88% against PS, 92.54% against CBC, 91.72% against BD, 73.73% against TBC, and 83.43% against BBC. In contrast, compounds **6k** and **6t** showed only moderate activity against CBC, with inhibition rates of 73.73% and 79.10%, respectively—all higher than that of pyrimethanil (70.49%). However, their activities against MO, TBC, and BBC were relatively low.

Based on the above results, the target compound, which exhibited antifungal activity higher than 60%, was selected to determine its median effective concentration (EC_50_) values against BD, PS, CBC, BBC, TBC, and MO, the results are summarized in Table 2. The EC_50_ of compound **6p** against BD, PS, CBC, BBC, TBC, and MO were 8.92, 13.11, 12.6, 21.91, 26.84, and 31.32 μg/mL, respectively. All these values were far below those of the commercial fungicide pyrimethanil (37.53, 32.41, 40.76, 28.64, 35.49, and 43.33 μg/mL, respectively, demonstrating the superior in vitro efficacy of **6p**.

### 3.3. SAR Analysis

Based on the in vitro antifungal activity data of target compounds **6a**–**6t**, the structure-activity relationships (SAR) of trifluoromethylpyrimidine-aniline derivatives were analyzed. The electronic properties and spatial arrangement of substituents on the pyrimidine ring jointly determine both the potency and breadth of antifungal activity.

(1) Effect of 6-substituent electronic properties on activity. Comparing compounds **6p** (2-chloro-6-aminopyrimidin-4-yl) and **6o** (2-chloro-6-trifluoromethylpyrimidin-4-yl), which share a common 2-chloro group, reveals that the electron-donating amino group (91.31% inhibition against BD) confers significantly higher activity than the strongly electron-withdrawing trifluoromethyl group (32.84%).
(2) Influence of the 6-Substituent Electronic Nature on Activity. This trend is particularly pronounced in 6-amino derivatives. Compound **6p** (2-Cl) exhibits the most potent and broad-spectrum activity, inhibiting all six pathogens by over 70%. Substituting the 2-Cl with an electron-donating methylthio group (**6t**, 2-SCH_3_-6-NH_2_) drastically reduces the inhibition against BD to 29.88%; however, **6t** retains moderate activity against PS (70.5%) and CBC (79.1%). The 2-unsubstituted analog **6h** (6-NH_2_) yields only 21.89% inhibition against BD. Similarly, the 2-amino derivative **6s** (2,6-di-NH_2_-Pyrimidin-4-yl-) shows weak activity (44.08%). These findings indicate that a weak electron-withdrawing group at the 2-position (e.g., Cl) optimizes molecular charge distribution to sustain high activity. Conversely, electron-donating groups (e.g., -SCH_3_) compromise the efficacy against BD but may still exhibit moderate inhibitory effects on other fungal species.

In summary, the 2-chloro-6-aminopyrimidin-4-yl motif (exemplified by **6p**) constitutes the key pharmacophore responsible for the potent and broad-spectrum antifungal activity within this series. The 6-amino group is crucial for conferring high potency, whereas the 2-chloro substituent plays a pivotal role in broadening the antifungal spectrum and enhancing overall efficacy. Together, these results suggest that an electron-donating group at the 6-position (e.g., -NH_2_), combined with a weakly electron-withdrawing substituent at the 2-position (e.g., -Cl), provides an optimal balance of electronic distribution and moderate steric bulk, likely promoting favorable interactions with the biological target(s). By contrast, strongly electron-withdrawing (e.g., -CF_3_) or bulkier/more polar groups (e.g., -SCH_3_) at these positions tend to impair activity, especially against BD.

### 3.4. In Vivo Activity of Compounds Against Kiwifruit Soft Rot

**Figure 3:**
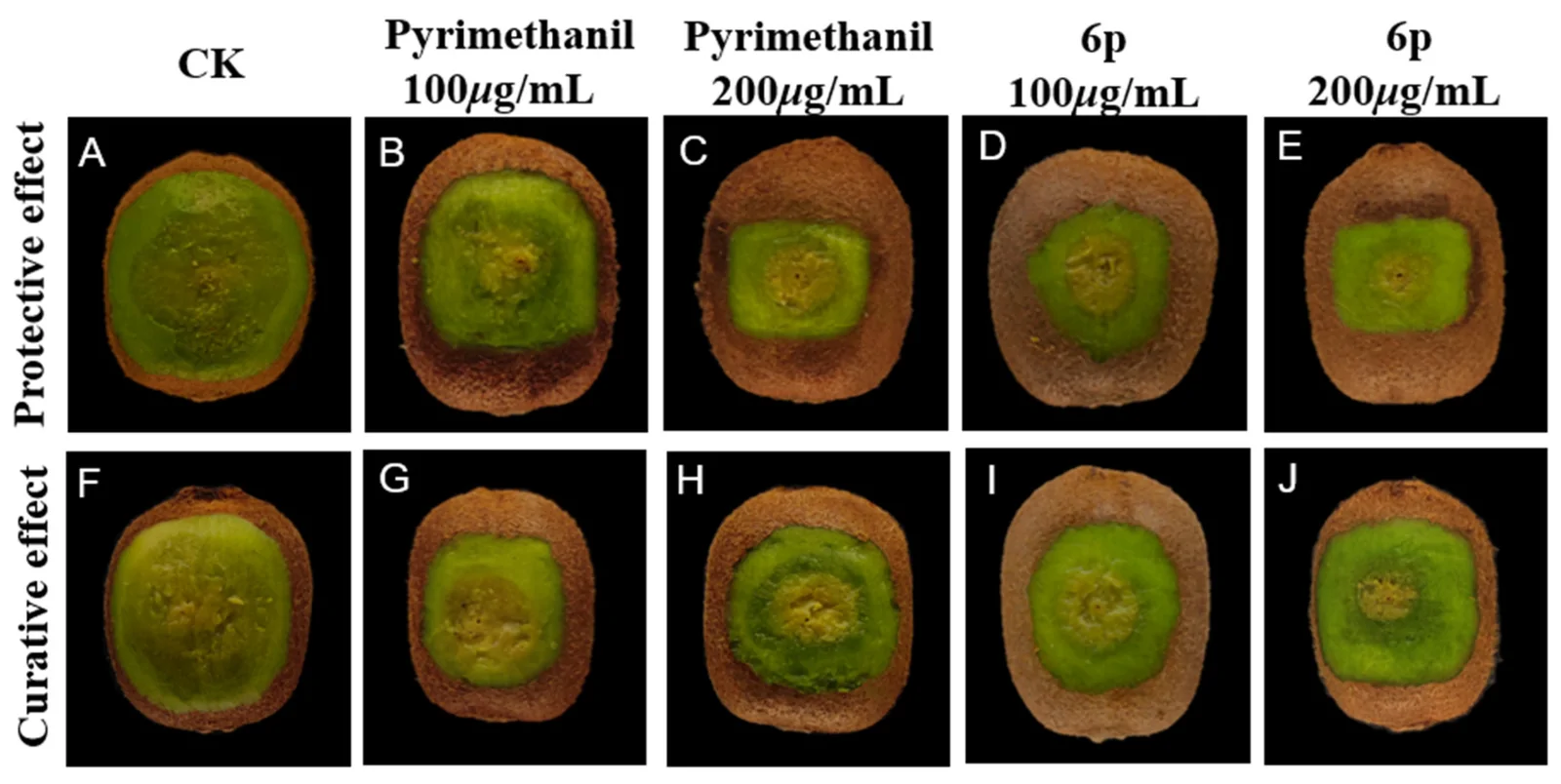
Kiwifruit treated with DMSO (**A**,**F**), Pyrimethanil (**B**,**C**,**G**,**H**), and **6p** (**D**,**E**,**I**,**J**). (**A**–**E**) Protective effects; (**F**–**J**) Curative effects.

Based on the excellent antifungal activity in vitro, compound **6p** was selected to further evaluate its inhibitory effect on BD on kiwifruit. The in vivo results are presented in Table 3 and Figure 3. Compound **6p** can effectively reduce the incidence of soft rot caused by BD. At concentrations of 100 and 200 μg/mL, compound **6p** exhibited protective efficacies of 62.13% and 77.81% on kiwifruit, respectively, outperforming the commercial fungicide pyrimethanil (44.97% and 60.65%). Similarly, the curative efficacies of compound **6p** were 59.45% and 75.35%, respectively, which were superior to those of pyrimethanil (38.16% and 58.64%). The above results indicate that compound **6p** is a potential antifungal candidate for the prevention and control of BD, the causal agent of kiwifruit soft rot.

### 3.5. Mechanism of Action

#### 3.5.1. Scanning Electron Microscopy (SEM) Observations

**Figure 4:**
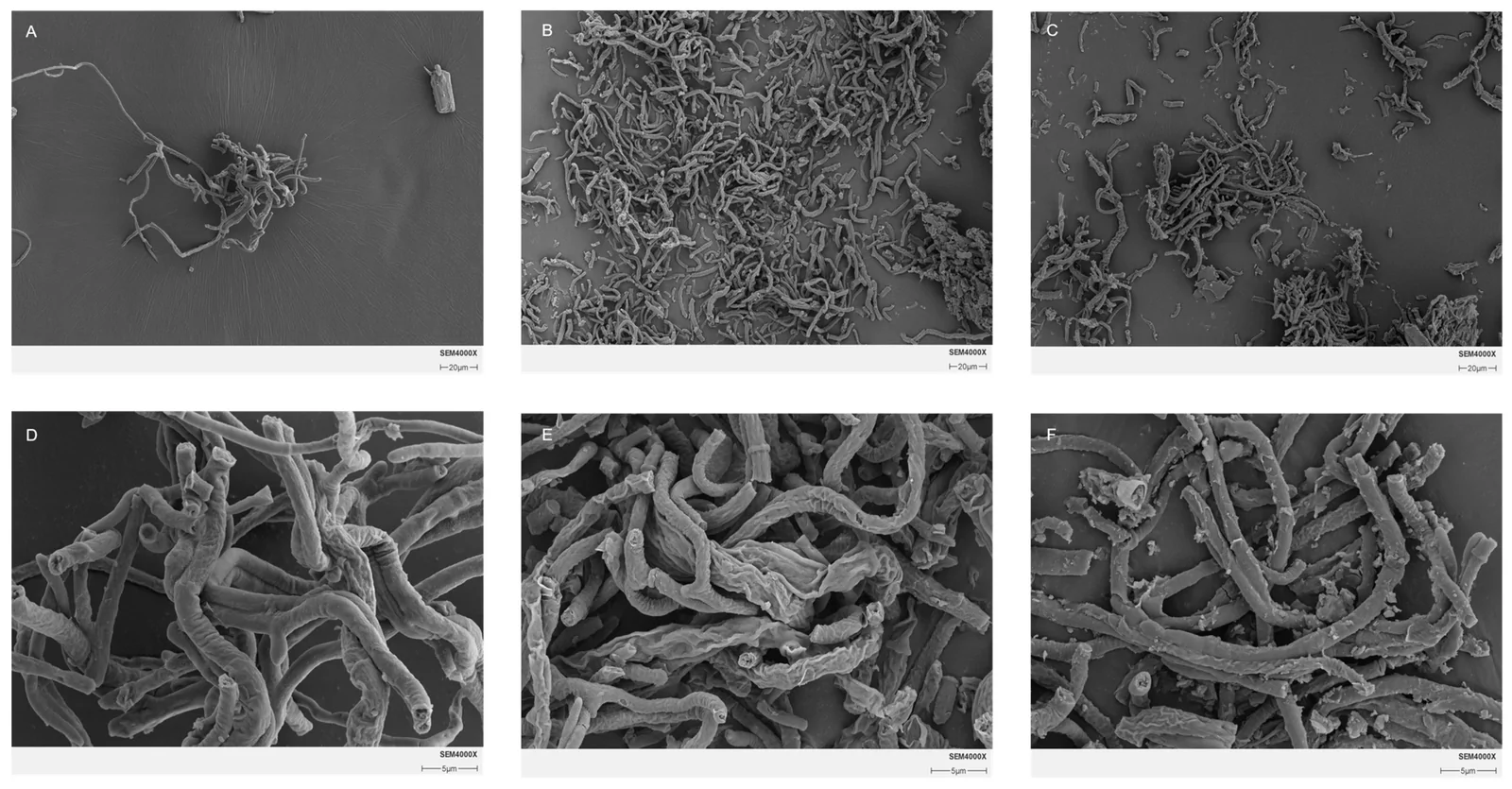
Scanning electron micrographs following treatment: (**A**,**D**) Blank control treated with DMSO; (**B**,**E**) Treated with Pyrimethanil at 50 μg/mL (**C**,**F**) Treated with compound **6p** at 50 μg/mL. Scale bars: 20 μm (**A**–**C**) and 5 μm (**D**–**F**).

We used SEM to examine the morphological effects of compound **6p** at 50 μg/mL on BD. The untreated mycelium was smooth and intact. After treatment with pyrimethanil at 50 μg/mL, the mycelium appeared rough and shriveled (Figure 4). However, compound **6p** treatment at 50 μg/mL caused clear structural changes. At high concentrations, the mycelium showed shrinkage, fracture, and surface roughness. Compound **6p** at 50 μg/mL causes cell death in BD, which supports its potential to control postharvest soft rot of kiwifruit.

#### 3.5.2. Measurement of Mycelium Dry Weight, Membrane Permeability, and Leakage of Cellular Contents

**Figure 5:**
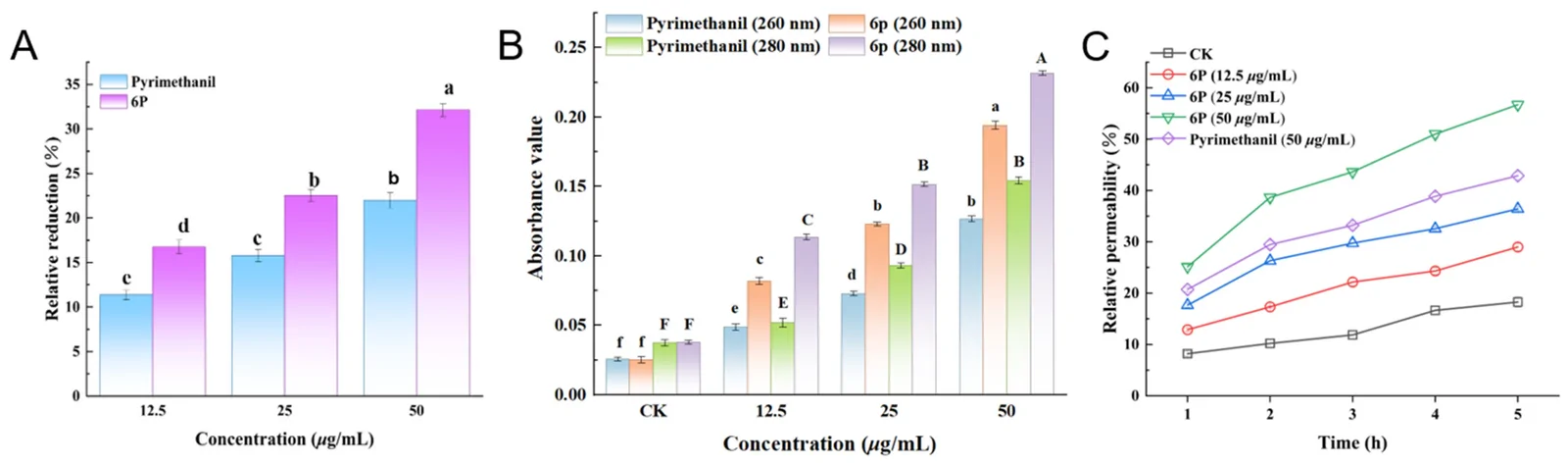
(**A**) Relative reduction rate of mycelial dry weight treated with 6p and pyrimethanil at 12.5, 25, and 50 μg/mL. Data are presented as mean ± SD (*n* = 3). Different lowercase letters (a–d) indicate significant differences (*p* < 0.05). (**B**) Leakage of nucleic acids (OD260) and proteins (OD280). Lowercase letters (a–f) indicate significance for OD260; uppercase letters (A–F) for OD280 (*p* < 0.05). (**C**) Data points represent mean ± SD (*n* = 3). Linear regression slopes of relative conductivity (%/h) were 2.68 ± 0.06 for CK, 3.58 ± 0.16 for **6p** 12.5 μg/mL, 4.55 ± 0.10 for **6p** 25 μg/mL, 5.52 ± 0.16 for Pyrimethanil 50 μg/mL, and 7.55 ± 0.12 for **6p** 50 μg/mL. One-way ANOVA indicated significant differences among groups (*p* < 0.05).

Mycelial biomass was measured to quantify growth inhibitory efficacy (Figure 5A). One-way ANOVA revealed significant differences among treatment groups (*p* < 0.001). Duncan’s multiple range test further showed that **6p** at 50 μg/mL exhibited the highest inhibition rate (32.40%), significantly greater than pyrimethanil at the same concentration (22.55%, *p* < 0.05). Notably, the inhibition rate of **6p** at 25 μg/mL (21.95%) was comparable to that of pyrimethanil at 50 μg/mL (22.53%), indicating that **6p** achieves superior efficacy at lower concentrations.

Intracellular constituent leakage was quantified by measuring OD260 and OD280 (Figure 5B). One-way ANOVA revealed significant differences among treatment groups for both OD260 and OD280 (*p* < 0.001). Duncan’s multiple range test showed that **6p** at 50 μg/mL caused the highest leakage of nucleic acids, significantly greater than pyrimethanil at the same concentration (*p* < 0.05). For protein leakage, **6p** at 50 μg/mL was comparable to pyrimethanil at 50 μg/mL, with no significant difference between them. These results indicate that **6p** disrupts membrane integrity, particularly affecting nucleic acid leakage.

Membrane integrity was further assessed by measuring conductivity (Figure 5C). Compound **6p** induced a concentration- and time-dependent increase in electrolyte leakage. One-way ANOVA revealed significant differences among treatment groups at each time point (*p* < 0.05). After 5 h, **6p** at 50 μg/mL exhibited the highest relative conductivity, significantly greater than CK and the 12.5 and 25 μg/mL treatments (*p* < 0.05). These results confirm that **6p** disrupts cell membrane integrity, consistent with the observed leakage of nucleic acids and proteins.

#### 3.5.3. Determination of ROS

**Figure 6:**
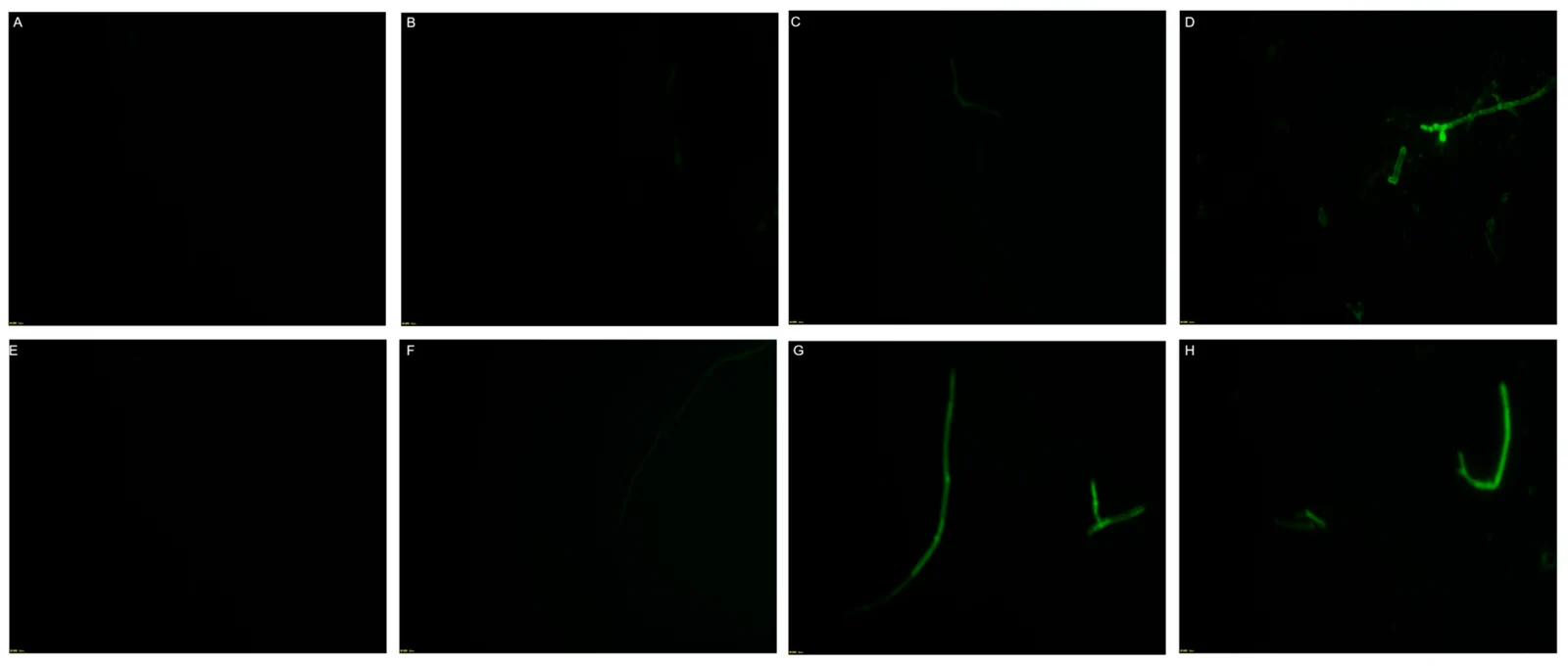
Fluorescence microscopy image of mycelia: Blank control group (**A**,**E**); Pyrimethanil group ((**B**), 12.5 μg/mL; (**C**), 25 μg/mL; (**D**), 50 μg/mL); Compound **6p** group ((**F**), 12.5 μg/mL; (**G**), 25 μg/mL; (**H**), 50 μg/mL).

ROS can induce lipid peroxidation, compromise membrane integrity, increase permeability, and ultimately cause cell death. As shown in Figure 6, compound **6p**-treated hyphae displayed bright green fluorescence, which intensified at higher concentrations. In contrast, the pyrimethanil group showed only a weak green signal. These results indicate that compound **6p** triggers intracellular ROS generation in the fungi, which then drives membrane lipid peroxidation.

#### 3.5.4. Superoxide Dismutase (SOD) Enzyme Assay

**Figure 7:**
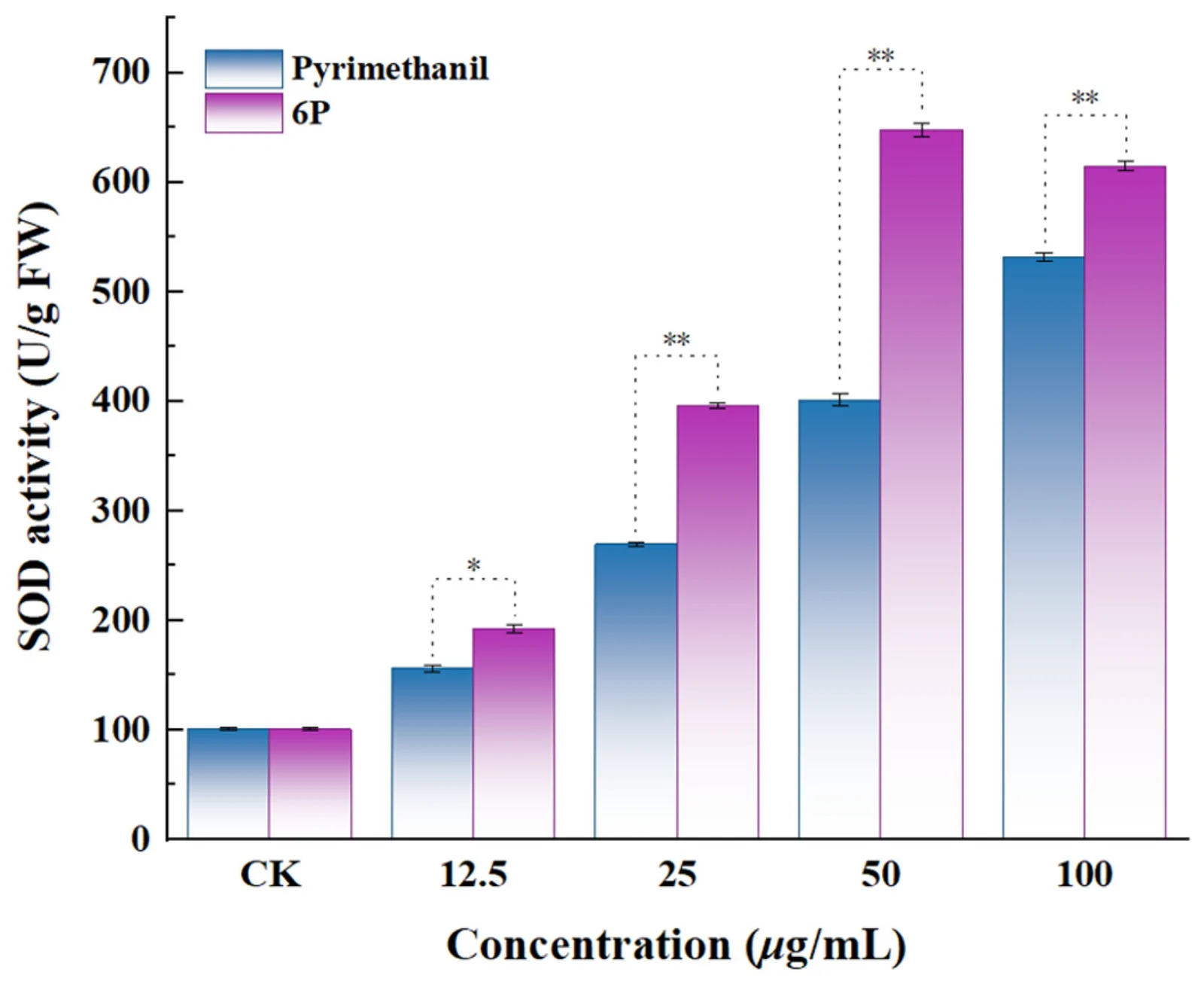
Effect of compound **6p** and pyrimethanil on superoxide dismutase (SOD) activity in BD mycelia. Data are presented as mean ± SD (*n* = 3). Statistical significance between compound **6p** and pyrimethanil at the same concentration was determined by independent samples *t*-test (SPSS): * *p* < 0.05, ** *p* < 0.01. CK, untreated control.

The effect of compound **6p** on the oxidative stress response was evaluated by measuring SOD activity in BD mycelia (Figure 7). Data were analyzed using independent samples *t*-test (SPSS 26.0) to compare the activities between compound **6p** and pyrimethanil at each tested concentration. At 12.5 μg/mL, compound **6p** induced a significantly higher SOD activity than pyrimethanil (* *p* < 0.05). At 25, 50, and 100 μg/mL, compound **6p** also led to higher SOD activities than pyrimethanil, and the differences were highly significant (** *p* < 0.01) at all three concentrations. However, at 100 μg/mL, SOD activity of **6p** declined to 620 U/g FW, significantly lower than at 50 μg/mL (*p* < 0.05), indicating that high concentrations of **6p** caused severe cellular damage that compromised enzymatic function. In contrast, pyrimethanil induced a monotonic increase in SOD activity up to 100 μg/mL. These results confirm that **6p** disrupts the ROS-scavenging system, particularly SOD, leading to toxic ROS accumulation and contributing to fungal growth inhibition.

### 3.6. Molecular Docking of Compound 6p and Pyrimethanil with Cystathionine γ-Synthase (CGS, PDB ID: 1I48)

**Figure 8:**
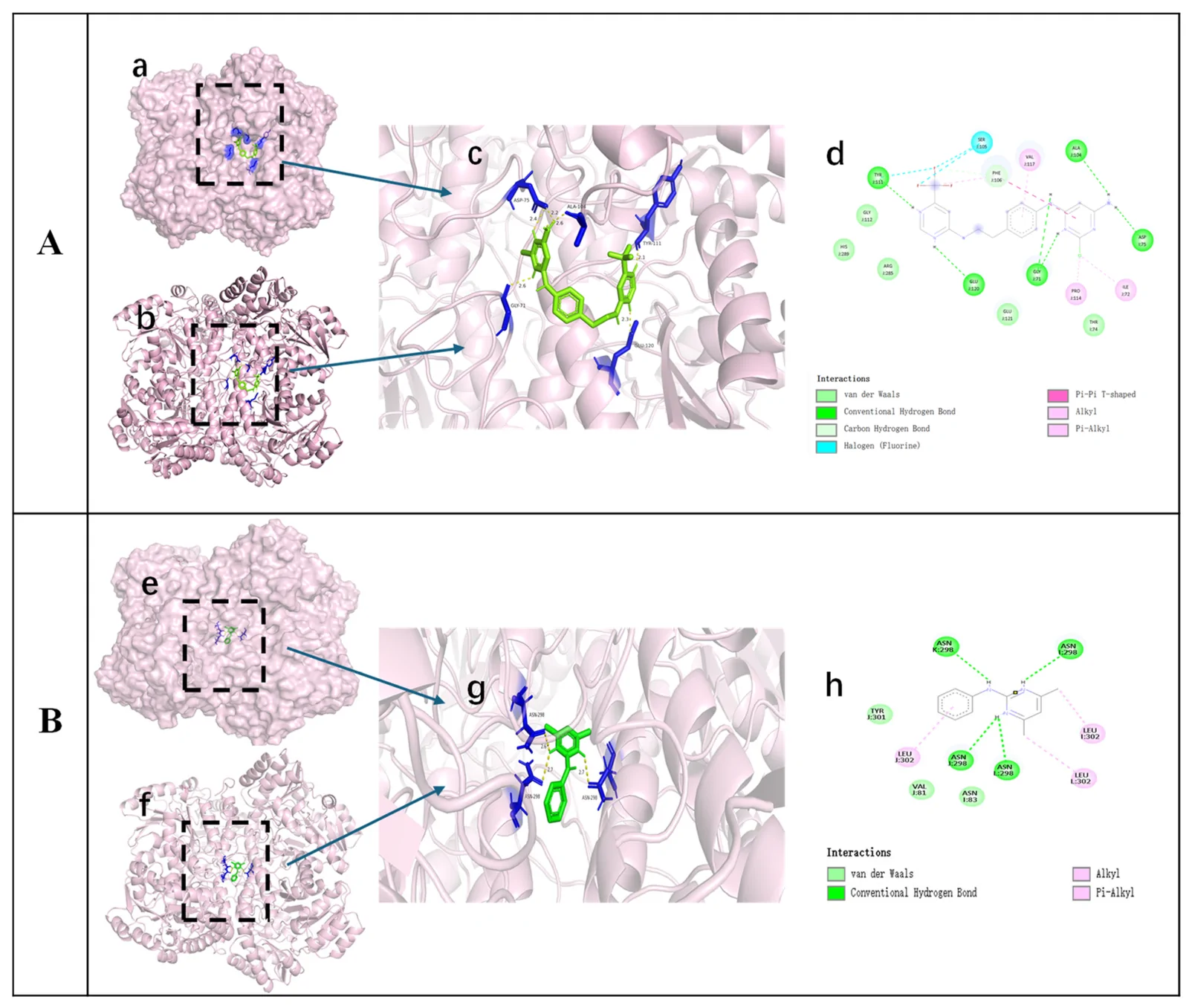
Molecular docking of compound **6p** (**A**) and pyrimethanil (**B**) with 1I48. (**A**) Compound **6p**: (**a**) Three-dimensional model of 1i48. (**b**,**c**) The optimal conformer of compound **6p** docks with the binding pocket of 1i48. (**d**) The binding mode and intermolecular interaction of compound 6p and 1i48. (**B**) pyrimethanil: (**e**) Three-dimensional model of 1i48. (**f**,**g**) The optimal conformer of compound pyrimethanil docks with the binding pocket of 1i48. (**h**) The binding mode and intermolecular interaction of compound Pyrimethanil and 1i48.

Molecular docking simulations were performed to predict the binding modes of compound **6p** and pyrimethanil with cystathionine γ-synthase (CGS, PDB ID: 1I48). The docking parameters and receptor preparation followed the procedures described in Section 2.11. Docking positioned compound **6p** inside the substrate-binding pocket of CGS, where it formed multiple contacts with adjacent residues. Notably, **6p** formed hydrogen bonds with Asp-75, Ala-104, and Gly-71, with bond distances of 2.4, 2.6, and 2.6 Å, respectively (Figure 8A). The binding was further stabilized by van der Waals forces and π-π stacking interactions. The **6p**-1I48 complex had a binding free energy of −8.2 kcal/mol; the commercial fungicide pyrimethanil scored −7.8 kcal/mol with the same protein. Pyrimethanil formed hydrogen bonds with Asn-298 (2.6 and 2.7 Å) (Figure 8B).

Docking results suggest that **6p** binds the target pocket more tightly than pyrimethanil, owing to a broader and more cooperative set of interactions. The **6p**-1I48 complex is stabilized by multiple non-covalent interactions, including van der Waals contacts, C–H···π interactions, π–π and π–alkyl stacking, and a halogen bond. Collectively, these contacts—rather than any single dominant force—likely account for the superior binding stability of **6p**. These computational results therefore offer a rationale for the enhanced antifungal efficacy observed.

## 4. Discussion

Applying the active fragment splicing strategy [42], we designed and synthesized a series of trifluoromethyl-substituted dipyrimidinamine derivatives, among which compound **6p** exhibited the most potent and broad-spectrum antifungal activity, significantly outperforming the commercial fungicide pyrimethanil. Structure–activity analysis highlighted the 2-chloro-6-amino substitution on the pyrimidine ring as a critical pharmacophore, while the trifluoromethyl group likely contributes to enhanced lipophilicity and target affinity [54]. The compound also showed promising protective and curative effects in fruit assays, supporting its potential as a practical alternative for postharvest disease management [55,56]. It should be noted that the concentrations used in these in vivo assays were 100 and 200 μg/mL, which are screening doses selected to ensure robust bioactivity under controlled laboratory conditions; they do not represent final field application rates or expected residue levels at harvest. Regulatory Maximum Residue Limits (MRLs) apply to residues on the commodity. For context, the current Maximum Residue Limit (MRL) for pyrimethanil on pome fruits is 10 mg/kg in China (GB 2763), while in markets such as the EU and USA, a default limit of 0.01 mg/kg applies in the absence of a specific tolerance for kiwifruit. Future field studies will be necessary to optimize application dosages and to evaluate residue levels in relation to these regulatory standards.

Mechanistic studies revealed that **6p** exerts its fungicidal effect through a multi-faceted mode of action. While molecular docking simulations suggest that **6p** may interact with enzymes in the methionine biosynthesis pathway (similar to classical anilinopyrimidines), our physiological experiments reveal a distinct and dominant phenotype: severe membrane disruption and oxidative stress. The strategic introduction of the trifluoromethyl group is likely a key driver of this physical effect; its high lipophilicity facilitates deep penetration into the fungal lipid bilayer, leading to the observed structural compromise of the membrane. Concurrently, **6p** triggered excessive reactive oxygen species (ROS) accumulation, overwhelming the fungal antioxidant defense system and leading to irreversible cellular damage [57]. Therefore, **6p** functions not only as a potential metabolic inhibitor but also as a pro-oxidant agent that physically compromises the cell, offering a broader mechanism than single-site inhibitors [58].

It should be noted, however, that compound **6p** shares the pyrimidinamine core with pyrimethanil, and cross-resistance cannot be excluded without experimental validation using resistant field strains. Future work should address this question to fully assess the practical utility of **6p** in resistance management programs.

## 5. Conclusions

In summary, compound **6p** represents a promising antifungal lead for managing postharvest kiwifruit soft rot, demonstrating superior potency against susceptible strains compared to the commercial fungicide pyrimethanil. Although **6p** retains the pyrimidinamine core, its distinct physiological effects—specifically, severe membrane disruption and pro-oxidant activity—suggest a multi-target mode of action. Furthermore, the strategic introduction of a trifluoromethyl group significantly enhanced bioavailability, representing a successful optimization of the chemical scaffold to achieve greater molecular efficacy. This design strategy may contribute to more reduced environmental impact by potentially reducing chemical inputs, although the risk of cross-resistance with existing fungicides remains to be validated in future studies using resistant field isolates.

## Figures and Tables

**Table 1:** Antifungal activities at the concentration of 50 μg/mL.

Compounds	Inhibition Rate (%)					
	MO	PS	CBC	BD	TBC	BBC
**6a**	30.72 ± 1.14	20.94 ± 0.73	28.36 ± 0.78	10.36 ± 0.53	16.42 ± 0.67	18.60 ± 0.53
**6b**	31.88 ± 1.03	13.57 ± 0.92	26.27 ± 1.30	33.14 ± 0.60	10.75 ± 0.75	35.17 ± 0.54
**6c**	29.86 ± 0.92	19.76 ± 0.73	21.79 ± 0.84	25.15 ± 0.86	28.66 ± 0.85	25.87 ± 0.58
**6d**	58.55 ± 1.24	41.59 ± 1.09	37.91 ± 0.96	26.92 ± 0.71	68.36 ± 1.24	59.88 ± 1.17
**6e**	62.32 ± 1.56	31.56 ± 0.95	36.42 ± 0.80	31.66 ± 0.61	22.39 ± 0.70	76.74 ± 1.20
**6f**	19.42 ± 0.85	25.66 ± 0.88	16.42 ± 0.67	18.93 ± 0.71	34.33 ± 1.07	24.13 ± 0.88
**6g**	29.86 ± 0.92	34.81 ± 0.94	11.34 ± 0.66	26.04 ± 1.14	16.72 ± 0.68	72.97 ± 1.12
**6h**	15.65 ± 0.95	26.55 ± 0.78	14.33 ± 0.74	21.89 ± 1.23	20.60 ± 0.69	21.22 ± 0.82
**6i**	46.52 ± 1.27	41.59 ± 1.09	33.73 ± 0.82	26.04 ± 0.75	25.67 ± 0.73	34.30 ± 0.60
**6j**	40.29 ± 1.01	18.58 ± 0.77	36.12 ± 0.78	22.78 ± 0.57	38.81 ± 0.75	20.35 ± 0.85
**6k**	42.61 ± 1.09	65.19 ± 1.47	73.73 ± 1.71	75.74 ± 1.18	43.28 ± 1.02	46.22 ± 1.03
**6l**	30.14 ± 0.89	25.07 ± 0.76	23.88 ± 0.72	18.34 ± 0.60	18.21 ± 0.68	21.80 ± 0.68
**6m**	13.91 ± 0.83	26.25 ± 0.74	14.63 ± 0.81	83.14 ± 1.54	11.34 ± 0.66	20.35 ± 0.54
**6n**	64.06 ± 1.62	75.22 ± 1.95	31.94 ± 0.81	84.02 ± 1.73	69.25 ± 1.48	42.73 ± 0.99
**6o**	36.52 ± 0.99	11.50 ± 0.74	23.28 ± 0.66	32.84 ± 0.54	34.63 ± 0.78	19.77 ± 0.60
**6p**	70.72 ± 1.58	77.88 ± 2.30	92.54 ± 3.39	91.72 ± 2.21	73.73 ± 1.41	83.43 ± 1.54
**6q**	24.35 ± 0.89	22.71 ± 0.88	23.28 ± 1.27	24.85 ± 0.74	34.63 ± 0.78	40.41 ± 0.79
**6r**	13.91 ± 0.83	38.05 ± 1.05	31.64 ± 0.87	33.43 ± 0.62	18.21 ± 0.68	71.80 ± 1.31
**6s**	27.54 ± 0.90	23.89 ± 0.93	43.58 ± 1.45	44.08 ± 1.35	29.25 ± 0.75	24.13 ± 0.57
**6t**	52.17 ± 1.48	70.50 ± 1.39	79.10 ± 1.64	29.88 ± 0.60	25.07 ± 0.83	36.05 ± 0.61
Pyrimethanil	55.43 ± 2.57	73.96 ± 4.65	70.49 ± 1.34	77.17 ± 3.23	65.11 ± 3.76	75.05 ± 2.28

**Table 2:** EC_50_ values of target compounds against phytopathogenic fungi.

Pathogens	Compounds	Regression Equation	R^2^	EC_50_ (µg/mL)	95% Confidence Interval (µg/mL)
**MO**	**6e**	*y* = 2.7388*x* + 0.5337	0.9904	42.73	38.98–47.87
	**6n**	*y* = 2.9353*x* + 0.3588	0.9936	38.12	34.87–42.05
	**6p**	*y* = 1.0493*x* + 3.4305	0.9938	31.32	24.45–41.77
	Pyrimethanil	*y* = 1.1155*x* + 3.1742	0.9831	43.33	33.05–54.41
**PS**	**6k**	*y* = 1.0585*x* + 3.5998	0.9884	21.03	16.25–28.92
	**6n**	*y* = 1.227*x* + 3.5808	0.9783	14.47	11.43–18.03
	**6p**	*y* = 1.3068*x* + 3.5396	0.9904	13.11	10.61–16.31
	**6t**	*y* = 1.238*x* + 3.4711	0.9867	17.17	13.69–21.85
	Pyrimethanil	*y* = 2.5491*x* + 1.149	0.974	32.41	29.48–36.36
**C** **B** **C**	**6k**	*y* = 1.389*x* + 3.3493	0.9878	15.43	12.15–18.31
	**6** **p**	*y* = 2.1726*x* + 2.609	0.9966	12.6	11.06–14.67
	**6t**	*y* = 1.3378*x +* 3.4435	0.9831	14.57	12.09–18.46
	Pyrimethanil	*y* =2.2996*x* + 1.2971	0.9833	40.76	34.64–45.48
**BD**	**6k**	*y* = 1.1901*x +* 3.6977	0.9941	12.42	9.81–13.58
	**6n**	*y* = 1.4798*x +* 3.4905	0.9995	10.47	8.57–12.59
	**6m**	*y* = 1.432*x +* 3.5075	0.9964	11.02	9.01–13.39
	**6p**	*y* = 1.5952*x* +3.4442	0.9945	8.92	7.32–10.64
	Pyrimethanil	*y* = 1.1302x + 3.2207	0.9936	37.53	33.57–42.31
**TBC**	**6d**	*y* = 2.1186*x +* 1.8024	0.9902	32.31	29.58–38.13
	**6n**	*y* = 2.1111*x +* 1.8348	0.9847	31.57	28.11–36.59
	**6p**	*y* = 1.9557*x +* 2.2057	0.981	26.84	23.58–31.13
	Pyrimethanil	*y* = 2.1356*x +* 1.6895	0.9895	35.49	31.45–41.29
**BBC**	**6e**	*y* = 1.9462*x +* 2.3107	0.9852	24.09	21.79–28.76
	**6g**	*y* = 1.7469*x +* 2.5202	0.9749	26.27	23.42–31.76
	**6p**	*y* = 2.212*x +* 2.0346	0.9782	21.91	19.12–24.97
	**6** **r**	*y* = 2.0719*x +* 1.9328	0.9794	30.22	26.85–35.03
	Pyrimethanil	*y* = 2.1605*x +* 1.8521	0.9787	28.64	24.71–32.11

**Table 3:** In Vivo Activity of **6p** against *B. dothidea* in Kiwifruit.

Compound	Concentration (μg/mL)	Protective Activity		Curative Activity	
		Lesion Length (cm)	Protective Effectiveness (%)	Lesion Length (cm)	Curative Effectiveness (%)
**6p**	200	0.75 ± 0.06 d	77.81	0.87 ± 0.07 d	75.35
	100	1.28 ± 0.05 c	62.13	1.43 ± 0.05 c	59.49
Pyrimethanil	200	1.33 ± 0.07 c	60.65	1.46 ± 0.06 c	58.64
	100	1.86 ± 0.07 b	44.97	2.09 ± 0.06 b	38.16
CK	-	3.38 ± 0.16 a	-	3.53 ± 0.17 a	-

Data are the average of three replicates. One-way ANOVA combined with Duncan’s multiple range test was used for statistical analysis. Different lowercase letters within the same column indicate significant differences among treatments at *p* < 0.05 based on lesion length. Pyrimethanil was used as positive control fungicide, and CK refers to the untreated negative control group.

## Data Availability

All data supporting the findings of this study are included within the article and its Supplementary Materials.

## Supplementary Materials

The following supporting information can be downloaded at: https://www.mdpi.com/article/10.3390/jof12090645/s1, 1.1H NMR, 13C NMR and HRMS; 2. 1H NMR, 13C NMR and HRMS Spectra (Figures S1–S60); Figure S1. 1H NMR Spectrum of of **6a**; Figure S2. 13C NMR Spectrum of **6a**; Figure S3. HRMS Spectrum of **6a**; Figure S4. 1H NMR Spectrum of **6b**; Figure S5. 13C NMR Spectrum of **6b**; Figure S6. HRMS Spectrum of **6b**; Figure S7. 1H NMR Spectrum of **6c**; Figure S8. 13C NMR Spectrum of **6c**; Figure S9. HRMS Spectrum of **6c**; Figure S10. 1H NMR Spectrum of **6d**; Figure S11. 13C NMR Spectrum of **6d**; Figure S12. HRMS Spectrum of **6d**; Figure S13. 1H NMR Spectrum of **6e**; Figure S14. 13C NMR Spectrum of **6e**; Figure S15. HRMS Spectrum of **6e**; Figure S16. 1H NMR Spectrum of **6f**; Figure S17. 13C NMR Spectrum of **6f**; Figure S18. HRMS Spectrum of **6f**; Figure S19. 1H NMR Spectrum of **6g**. Figure S20. 13C NMR Spectrum of **6g**; Figure S21. HRMS Spectrum of **6g**; Figure S22. 1H NMR Spectrum of **6h**; Figure S23. 13C NMR Spectrum of **6h**; Figure S24. HRMS Spectrum of **6h**; Figure S25. 1H NMR Spectrum of **6i**; Figure S26. 13C NMR Spectrum of **6i**; Figure S27. HRMS Spectrum of **6i**; Figure S28. 1H NMR Spectrum of **6j**; Figure S29. 13C NMR Spectrum of **6j**; Figure S30. HRMS Spectrum of **6j**; Figure S31. 1H NMR Spectrum of **6k**; Figure S32. 13C NMR Spectrum of **6k**; Figure S33. HRMS Spectrum of **6k**; Figure S34. 1H NMR Spectrum of **6l**; Figure S35. 13C NMR Spectrum of **6l**; Figure S36. HRMS Spectrum of **6l**; Figure S37. 1H NMR Spectrum of **6m**; Figure S38. 13C NMR Spectrum of **6m**; Figure S39. HRMS Spectrum of **6m**; Figure S40. 1H NMR Spectrum of **6n**; Figure S41. 13C NMR Spectrum of **6n**; Figure S42. HRMS Spectrum of **6n**; Figure S43. 1H NMR Spectrum of **6º**; Figure S44. 13C NMR Spectrum of **6o**; Figure S45. HRMS Spectrum of **6o**; Figure S46. 1H NMR Spectrum of **6p**; Figure S47. 13C NMR Spectrum of **6p**; Figure S48. HRMS Spectrum of **6p**; Figure S49. 1H NMR Spectrum of **6q**; Figure S50. 13C NMR Spectrum of **6q**; Figure S51. HRMS Spectrum of **6q**; Figure S52. 1H NMR Spectrum of **6r**; Figure S53. 13C NMR Spectrum of **6r**; Figure S54. HRMS Spectrum of **6r**; Figure S55. 1H NMR Spectrum of **6s**; Figure S56. 13C NMR Spectrum of **6s**; Figure S57. HRMS Spectrum of **6s**; Figure S58. 1H NMR Spectrum of **6t**; Figure S59. 13C NMR Spectrum of **6t**; Figure S60. HRMS Spectrum of **6t**.
