# Supplementary material for: Novel Trifluoromethyl-Substituted Dipyrimidinamine Derivatives as Potential Antifungal Agents for Controlling Postharvest Soft Rot in Kiwifruit
Source: J Fungi (Basel). 2026 Sep 1;12(9):645. doi: 10.3390/jof12090645 (PMC13608136; doi:10.3390/jof12090645)
Supplement: Supplementary file 1 [file jof-12-00645-s001.zip › jof-4446445-supplementary.pdf]

Supporting Information

Novel Trifluoromethyl-substituted Dipyrimidinamine Derivatives as Potential Antifungal Agents for Controlling Postharvest Soft Rot in Kiwifruit

Wenneng Wu*, Jingyan Zhao, Peng Geng, Qiang Fei, Haijiang Chen*

School of Food Science and Engineering, Guiyang University, Guiyang 550003, China; zhaojinyann@163.com(J.Z.), gengpeng231012@126.com(G.P.), fqorganic@163.com (Q.F.)

*Correspondence: wuwenneng123@126.com (W.W.), b05chenhj@126.com(C.H.)

1、 ^{1}H NMR, ^{13}C NMR and HRMS

N-(4-nitrophenethyl)-6-(trifluoromethyl)pyrimidin-4-amine (**4**): Yellow solid, yield 84.1%, m.p. 131.6-133.3◦C; ^{1}H NMR (600 MHz, DMSO-d_6, ppm) δ 8.58 (s, 1H, Pyrimidine-H), 8.16 (d, J = 8.43 Hz, 2H, Ph-H), (t, J = 5.10 Hz, 1H, -CH$_2$NH-), 7.54 (d, J = 8.41 Hz, 2H, Ph-H), 6.85 (s, 1H, Pyrimidine-H), 3.68 (q, J = 6.72 Hz, 2H, -CH$_2$NH-), 3.00 (t, J = 7.01 Hz, 2H, -CH2-).

N-(4-aminophenethyl)-6-(trifluoromethyl)pyrimidin-4-amine (**5**): Yellow solid, yield 78.8%, m.p. 98.8-101.5◦C; ^{1}H NMR (600 MHz, DMSO-d_6, ppm) δ 8.57 (s, 1H, pyrimidine-H), 8.05 (t, J = 5.40 Hz, 1H, -CH$_2$NH-), 6.90 (d, J = 8.04 Hz, 2H, Ph-H), 6.86 (s, 1H, pyrimidine-H), 6.51 (d, J = 10 Hz, 2H, Ph-H), 4.88 (s, 2H, -NH), 3.53 (q, J= 6.60 Hz, 2H, -CH$_2$NH-), 2.68 (t, J = 7.38 Hz, 2H, -CH$_2$-).

(2-((6-(trifluoromethyl)pyrimidin-4-yl)amino)ethyl)phenyl)pyrimidin-2-amine (**6a**): white solid; yield 45.4%; m.p. 163.4-165.1 °C; ^{1}H NMR (600 MHz, DMSO-d_6) δ 9.56 (s, 1H, -NH-), 8.58 (s, 1H, pyrimidine-H), 8.45 (d, J = 4.80 Hz, 2H, pyrimidine-H), 8.10 (t, J = 5.34 Hz, 1H, -CH$_2$NH-), 7.68 (d, J = 8.40 Hz, 2H, Ph-H), 7.16 (d, J = 8.40 Hz, 2H, Ph-H), 6.87 (s, 1H, pyrimidine-H), 6.81 (t, J = 4.74 Hz, 1H, pyrimidine-H), 3.61 (q, J = 6.78 Hz, 2H, -CH$_2$NH-), 2.81 (t, J = 7.26 Hz, 2H, -CH$_2$-); ^{13}C NMR (150 MHz, DMSO-d_6) δ 162.81, 160.51, 159.78, 158.43, 151.58(q, J = 33.60 Hz), 139.14, 132.48, 129.13, 122.45 (q, J = 272.51 Hz), 119.48, 112.64, 102.86, 42.26, 34.29; HRMS(ESI)m/z [M+H]$^+$ calcd for $C_{17}H_{16}N_6F_3$: 361.13831, found: 361.13841.

2-((4-(2-((6-(trifluoromethyl)pyrimidin-4-yl)amino)ethyl)phenyl)amino)pyrimidine-4-carbonitrile (**6b**): yellow solid; yield 53.6%; m.p. 207.1-208.9 °C; ^{1}H NMR (600 MHz, DMSO-d_6) δ 10.11 (s, 1H, -NH-), 8.74 (t, J = 4.4 Hz, 1H, pyrimidine-H), 8.58 (s, 1H, pyrimidine-H), 8.09 (s, 1H, pyrimidine-H), 7.60 (d, J = 4.7 Hz, 2H, Ph-H), 7.35 (t, J = 4.4 Hz, 1H, -NH-), 7.22 (d, J = 4.7 Hz, 2H, Ph-H), 6.87 (s, 1H, pyrimidine-H), 3.61-3.58 (m, 2H,-CH$_2$-), 2.83-2.80 (m, 2H, -CH$_2$-); ^{13}C NMR (150 MHz, DMSO-d_6) δ 167.44, 162.83, 161.42, 160.36, 159.76, 151.84 (q, J = 33.11 Hz), 140.96, 137.87, 133.96, 132.17, 131.98, 129.33, 124.26 (q, J = 272.58 Hz), 120.28, 116.61, 115.27, 102.86, 42.16, 34.31; HRMS(ESI)m/z [M+H]$^+$ calcd for $C_{18}H_{15}N_7F_3$: 386.13355, found: 386.13339

N^4-(4-(2-((6-(trifluoromethyl)pyrimidin-4-yl)amino)ethyl)phenyl)pyrimidine-4,6-diamine (**6c**): yellow solid; yield 40.7%; m.p. 142.3-145.1 °C; ^{1}H NMR (600 MHz, DMSO-d_6) δ 9.93 (s, 1H, -NH$_2$), 8.58 (s, 1H, pyrimidine-H), 8.30 (s, 1H, pyrimidine-H), 8.15 (t, J = 4.92 Hz, 1H, -CH$_2$NH-), 7.49 (d, J = 7.98 Hz, 2H, Ph-H), 7.13 (d, J = 7.86 Hz, 2H, Ph-H), 6.87 (s, 1H, pyrimidine-H), 5.87

(s, 1H, pyrimidine-H), 3.62 (q, J = 6.60 Hz, 2H, -CH2NH-), 2.84 (t, J = 7.26 Hz, 2H, -CH_2-); ^{13}C NMR (150 MHz, DMSO-d_6) δ 162.83, 159.78, 151.43 (q, J = 28.73 Hz), 146.00, 138.20, 136.58, 129.91, 128.56, 125.96, 123.18 (q, J = 276.53 Hz), 122.97, 102.86, 82.30, 42.08, 34.33; HRMS(ESI)m/z $[M+H]^+$ calcd for $C_{17}H_{17}N_7F_3$: 376.14921, found: 376.14886.

4-(Trifluoromethyl)-N-(4-(2-((6-(trifluoromethyl)pyrimidin-4-yl)amino)ethyl)phenyl)pyrimidin-2-amine (**6d**): white solid; yield 48.7%; m.p. 209.1-211.1 °C; ^1H NMR (600 MHz, DMSO-d_6) δ 10.15 (s, 1H, -NH-), 8.79 (d, J = 4.90 Hz, 1H, pyrimidine-H), 8.59 (s, 1H, pyrimidine-H), 8.10 (t, J = 4.98 Hz, 1H, -CH2NH-), 7.68 (d, J = 8.28 Hz, 2H, Ph-H), 7.23 (d,J = 4.74 Hz, 2H, Ph-H), 7.21 (s, 1H, pyrimidine-H), 6.88 (s, 1H, pyrimidine-H), 3.62 (q,J = 6.54 Hz, 2H, -CH2NH-), 2.83 (t, J = 7.14 Hz, 2H, -CH_2-); ^{13}C NMR (150 MHz, DMSO-d_6) δ 162.84, 162.04, 160.45, 159.76, 151.84 (q, J = 33.23 Hz), 138.15, 133.72, 129.29, 124.26 (q, J = 272.70 Hz), 123.78 (q, J = 273.63 Hz), 120.04, 107.41, 102.87, 42.16, 34.29; HRMS(ESI)m/z $[M+H]^+$ calcd for $C_{18}H_{15}N_6F_6$: 429.12569, found: 429.12564.

5-(Trifluoromethyl)-*N*-(4-(2-((6-(trifluoromethyl)pyrimidin-4-yl)amino)ethyl)phenyl)pyrimidin-2-amine (**6e**): white solid; yield 53.4%; m.p. 178.6-180.4 °C; ^1H NMR (600 MHz, DMSO-d_6) δ 10.27 (s, 1H, -NH-), 8.82 (s, 2H, pyrimidine-H), 8.59 (s, 1H, pyrimidine-H), 8.11 (t, J = 5.34 Hz, 1H, -CH2NH-), 7.67 (d, J = 8.28 Hz, 2H, Ph-H), 7.22 (d, J = 8.28 Hz, 2H, Ph-H), 6.87 (s, 1H, pyrimidine-H), 3.63 (q, J = 6.72 Hz, 2H, -CH2NH-), 2.84 (t, J = 7.08 Hz, 2H, -CH_2-), 2.38 (s, 2H, -CH_3), 2.23 (s, 3H, -CH_3). ^{13}C NMR (150 MHz, DMSO-d_6) δ 162.83, 161.84, 159.78, 156.35, 151.60 (q, J = 32.88 Hz), 137.76, 134.26, 129.30, 125.54 (q, J = 268.67 Hz), 122.45 (q, J = 272.25 Hz), 114.27 (q, J = 33.02 Hz), 102.87, 42.15, 34.31; HRMS(ESI)m/z $[M+H]^+$ calcd for $C_{18}H_{15}N_6F_6$: 429.12569, found: 429.12512.

6-Methoxy-*N*-(4-(2-((6-(trifluoromethyl)pyrimidin-4-yl)amino)ethyl)phenyl) pyrimidin-4-amine (**6f**): yellow solid; yield 50.3%; m.p. 131.1-133.7 °C; ^1H NMR (600 MHz, DMSO-d_6) δ 9.35 (s, 1H, -NH-), 8.59 (s, 1H, pyrimidine-H), 8.35 (s, 1H, pyrimidine-H), 8.11 (t, J = 5.34 Hz, 1H, -CH2NH-), 7.50 (d, J = 8.28 Hz, 2H, Ph-H), 7.21 (d, J = 8.28 Hz, 2H, Ph-H), 6.88 (s, 1H, pyrimidine-H), 6.06 (s, 1H, pyrimidine-H), 3.85 (s, 3H, -OCH_3), 3.61 (q, J = 6.72 Hz, 2H, -CH2NH-), 2.83 (t, J = 7.26 Hz, 2H, -CH_2-); ^{13}C NMR (150 MHz, DMSO-d_6) δ 169.83, 162.84, 162.48, 159.76, 158.23, 151.84 (q, J = 33.44 Hz), 138.60, 133.58, 129.49, 124.27 (q, J = 272.40 Hz), 120.63, 114.51, 102.85, 87.71, 53.80, 42.18, 34.30; HRMS(ESI)m/z $[M+H]^+$ calcd for $C_{18}H_{18}ON_6F_3$: 391.14887, found: 391.14859.

5-Bromo-*N*-(4-(2-((6-(trifluoromethyl)pyrimidin-4-yl)amino)ethyl)phenyl)pyrimidin-2-amine (**6g**): brown solid; yield 45.1%; m.p. 174.6-175.4 °C; ^{1}H NMR (600 MHz, DMSO-d_6) δ 9.79(s, 1H, -NH-), 8.59 (s, 1H, pyrimidine-H), 8.56 (s, 2H, pyrimidine-H), 8.11 (t, J = 5.46 Hz, 1H, -CH$_2$NH-), 7.63 (d, J = 8.34 Hz, 2H, Ph-H), 7.18 (d, J = 8.34 Hz, 2H, Ph-H), 6.88 (s, 1H, pyrimidine-H), 3.60 (q, J = 6.72 Hz, 2H, -CH$_2$), 2.82 (t, J = 7.20 Hz, 2H, -CH$_2$-); ^{13}C NMR (150 MHz, DMSO-d_6) δ, 162.82, 159.76, 158.86, 158.56, 151.82 (q, J = 33.50 Hz), 138.52, 133.20, 129.22, 12426 (q, J = 272.46 Hz), 119.84, 107.80, 102.86, 42.21, 34.29; HRMS(ESI)m/z [M+H]$^+$ calcd for $C_{17}H_{15}N_6BrF_3$:`439.04882, found:439.04855.

2-(Methylthio)-N-(4-(2-((6-(trifluoromethyl)pyrimidin-4-yl)amino)ethyl)phenyl)pyrimidin-4-amine (**6h**): white solid; yield 51.1%; m.p. 151.0-152.5 °C; ^{1}H NMR (600 MHz, DMSO-d_6) δ 9.60 (s, 1H, -NH$_2$), 8.58 (s, 1H, pyrimidine-H), 8.11 (t, J = 5.16 Hz, 1H, -CH$_2$NH-), 8.08 (d, J = 5.82 Hz, 1H, pyrimidine-H), 7.57 (d, J = 7.86 Hz, 2H, Ph-H), 7.22 (d, J = 8.22 Hz, 2H, Ph-H), 6.86 (s, 1H, pyrimidine-H), 6.46 (d, J = 5.82 Hz, 1H, pyrimidine-H), 3.62 (q, J = 6.66 Hz, 2H, -CH$_2$NH-), 2.83 (t, J = 7.14 Hz, 2H, -CH$_2$-), 2.46 (s, 3H, -CH$_3$); ^{13}C NMR (150 MHz, DMSO-d_6) δ 170.92, 162.82, 159.99, 159.76, 155.65, 151.81 (q, J = 33.17 Hz), 138.08, 133.89, 129.65, 129.47, 124.26 (q, J = 272.45 Hz), 120.57, 103.14, 102.86 (d, J = 3.23 Hz), 42.12, 34.29, 13.95; HRMS(ESI)m/z [M+H]$^+$ calcd for $C_{18}H_{18}N_6F_3S$: 407.12603, found: 407.1257.

N^4,N^4-dimethyl-N^6-(4-(2-((6-(trifluoromethyl)pyrimidin-4-yl)amino)ethyl) phenyl)pyrimidine-4,6-diamine (**6i**): brown solid; yield 42.6%; m.p. 177.4-179.1 °C; ^{1}H NMR (600 MHz, DMSO-d_6) δ 8.94 (s, 1H, -NH-), 8.58 (s, 1H, pyrimidine-H), 8.14 (s, 1H, pyrimidine-H), 8.09 (t, J = 5.5 Hz, 1H, -NH-), 7.50 (d, J = 8.3 Hz, 2H, Ph-H), 7.15 (d, J = 8.3 Hz, 2H, Ph-H), 6.87 (s, 1H, pyrimidine-H), 5.79 (s, 1H, pyrimidine-H), 3.60 (q, J = 6.7 Hz, 2H, -CH$_2$-), 2.99 (s, 6H, -CH$_3$), 2.80 (t, J = 7.3 Hz, 2H, -CH$_2$-); ^{13}C NMR (150 MHz, DMSO-d_6) δ 162.82, 162.68, 161.06, 159.77, 157.56, 151.18 (q, J = 29.85 Hz), 139.49, 129.34, 122.66 (q, J = 272.81 Hz), 120.05, 102.87, 83.36, 42.23, 37.11, 34.26; HRMS(ESI)m/z [M+H]$^+$ calcd for $C_{19}H_{21}N_7F_3$: 404.1805, found: 404.18021.

Ethyl 4-(trifluoromethyl)-2-((4-(2-((6-(trifluoromethyl)pyrimidin-4-yl)amino)ethyl)phenyl)amino)pyrimidine-5-carboxylate (**6j**): yellow solid; yield 57.2%; m.p. 191.1-193.7 °C; ^{1}H NMR (600 MHz, DMSO-d_6) δ 10.70 (s, 1H, -NH-),9.03 (s, 1H, pyrimidine-H), 8.59 (s, 1H, pyrimidine-H), 8.11 (t, J = 4.50 Hz, 1H, -CH$_2$NH-), 7.68 (d, J = 7.50 Hz, 2H, Ph-H), 7.25 (d, J = 7.92 Hz, 2H, Ph-H), 6.87 (s, 1H, pyrimidine-H), 4.32(q, J = 6.90 Hz, 2H, -CH$_2$-), 3.62

(q, J = 5.64 Hz, 2H, -CH2NH-), 2.85 (t, J = 6.84 Hz, 2H, $-CH_2-$), 1.32 (t, J = 7.02 Hz, 3H, $-CH_3$); ^{13}C NMR (150 MHz, DMSO-d_6) δ 163.24, 163.08, 162.83, 160.23, 159.78, 151.60 (q, J = 34.58 Hz), 137.20, 129.43, 122.45, 120.63, 112.98, 102.87, 61.95, 42.09, 34.31, 14.28; HRMS(ESI)m/z $[M+Na]^+$ calcd for $C_{21}H_{18}O_2N_6F_6Na$: 523.12876, found: 523.1283.

N^4-(4-(2-((6-(trifluoromethyl)pyrimidin-4-yl)amino)ethyl)phenyl)pyrimidine-2,4,6-triamine (**6k**):gray solid; yield 52.2%; m.p. 198.7-199.8 °C;^{1}H NMR (600 MHz, DMSO-d_6) δ 11.27 (s, 1H, $-NH_2$), 9.60 (s, 1H, -NH-), 8.58 (s, 1H, pyrimidine-H), 8.15 (t, J = 4.92 Hz, 1H, $-CH_2$NH-), 7.55-7.51 (m, 2H, Ph-H), 7.14 (d, J = 7.80 Hz, 2H, Ph-H), 6.89 (s, 1H, pyrimidine-H), 5.50 (s, 1H, pyrimidine-H), 3.62 (q, J = 6.66 Hz, 2H, -CH2NH-), 2.84 (t, J= 7.20 Hz, 2H, $-CH_2-$); ^{13}C NMR (150 MHz, DMSO-d_6) δ 162.85, 159.76, 154.53, 151.62 (q, J = 33.06 Hz), 145.68, 145.68, 138.43, 128.64, 125.96, 122.45 (q, J = 272.51 Hz),102.88, 42.15, 34.31; HRMS(ESI)m/z $[M+H]^+$ calcd for $C_{17}H_{17}N_3F_8$: 391.15982, found: 391.15873.

6-Methoxy-N^4-(4-(2-((6-(trifluoromethyl)pyrimidin-4-yl)amino)ethyl)phenyl)pyrimidine-2,4-diamine (**6l**): white solid; yield 42.5%; m.p. 190.2-192.8 °C; ^{1}H NMR (600 MHz, DMSO-d_6) δ 10.18 (s, 1H, -NH-), 8.58 (s, 1H, pyrimidine-H), 8.15 (s, 1H, $-CH_2$NH-), 7.52 (d, J = 7.98 Hz, 3H, Ph-H), 7.28 (d, J = 7.92 Hz, 2H, Ph-H), 7.14 (d, J = 8.22 Hz, 2H, Ph-H), 6.88 (s, 1H, pyrimidine-H), 5.63 (s, 1H, -NH), 3.14 (s, 3H, $-CH_3$), 3.63 (q, J = 6.66 Hz, 2H, -CH2NH-), 2.87 (t, J = 7.20 Hz, 2H, $-CH_2-$); ^{13}C NMR (150 MHz, DMSO-d_6) δ 162.85, 159.74, 155.99, 151.88 (q, J = 39.81 Hz), 145.73, 138.40, 129.86, 128.63, 125.95, 122.44 (q, J = 272.45 Hz), 102.87, 42.06, 34.32, 21.24; HRMS(ESI)m/z $[M+H]^+$ calcd for $C_{18}H_{18}F_3N_7O$: 406.15977, found: 406.15872.

6-methyl-N^4-(4-(2-((6-(trifluoromethyl)pyrimidin-4-yl)amino)ethyl)phenyl)pyrimidine-2,4-diamine (**6m**): yellow solid; yield 52.6%; m.p. 183.1-185.5 °C; ^{1}H NMR (600 MHz, DMSO-d_6)δ 12.31 (s, 1H, -NH-), 10.41 (s, 1H, pyrimidine-H), 8.59 (s, 1H, pyrimidine-H), 8.16 (t, J = 5.16 Hz, 1H, -NH-), 7.68 (s, 2H, N-H), 7.56 (d, J = 8.04 Hz, 2H, Ph-H), 7.17 (d, J = 7.86 Hz, 2H, Ph-H), 6.90 (s, 1H, pyrimidine-H), 3.63 (q, J = 6.60 Hz, 2H, $-CH_2-$), 2.85 (t, J = 7.20 Hz, 2H, $-CH_2-$), 2.30 (s, 3H, $-CH_3$); ^{1}C NMR (150 MHz, DMSO-d_6) δ 162.86, 159.74, 155.94, 151.84 (q, J = 30.08 Hz), 145.27, 138.72, 128.74, 125.94, 124.26(q, J = 272.55 Hz), 102.90, 42.09, 34.32, 21.23; HRMS(ESI)m/z $[M+H]^+$ calcd for $C_{18}H_{19}N_7F_3$: 390.16485, found: 390.16485.

2-chloro-N^4-(4-(2-((6-(trifluoromethyl)pyrimidin-4-yl)amino)ethyl)phenyl)pyrimidine-4,6-diamine (**6n**):yellow solid; yield 53.6%; m.p. 187.5-189.1 °C; ^{1}H NMR (600 MHz, DMSO-d_6) δ

9.31 (s, 1H, -NH-), 8.63 (s, 1H, pyrimidine-H), 8.12 (t, *J* = 5.28 Hz, 1H, -CH_2<u>NH</u>-), 7.70 (d, *J* = 8.46 Hz, 2H, Ph-H), 7.17 (d, *J* = 8.40 Hz, 2H, Ph-H), 6.95 (s, 2H, N-H), 6.91 (s, 1H, pyrimidine-H), 5.95 (s, 1H, pyrimidine-H), 3.63 (q, *J* = 6.66 Hz, 2H, -<u>CH_2</u>NH-), 2.84 (t, *J* = 7.14 Hz, 2H, -CH_2-); ^{13}C NMR (150 MHz, DMSO-d_6) δ 165.50, 162.82, 159.96, 159.77, 158.19, 151.61 (q, *J* = 40.18 Hz), 139.21, 132.39, 129.05, 124.29 (q, *J* =272.55 Hz), 102.86, 94.22, 42.28, 34.26; HRMS(ESI)m/z $[M+H]^+$ calcd for $C_{17}H_{16}N_7ClF_3$: 410.11023, found: 410.11008.

2-(methylthio)-N^4-(4-(2-((6-(trifluoromethyl)pyrimidin-4-yl)amino)ethyl)phenyl)pyrimidine-4,6-diamine (**6o**):pink solid; yield 57.8%; m.p. 140.3-142.9 °C;^{1}H NMR (600 MHz, DMSO-d_6)*δ* 8.85 (s, 1H, -NH-), 8.59 (s, 1H, pyrimidine-H), 8.10 (t, *J* = 5.46 Hz, 1H, -CH_2<u>NH</u>-), 7.44 (d, *J* = 8.28 Hz, 2H, Ph-H), 7.16 (d, *J* = 8.28 Hz, 2H, Ph-H), 6.87 (s, 1H, pyrimidine-H), 6.35 (s, 2H, -NH_2), 5.50 (s, 1H, pyrimidine-H), 3.61 (q, *J* = 6.78 Hz, 2H, -<u>CH_2</u>NH-), 2.81 (t, *J* = 7.32 Hz, 2H, -CH_2-), 2.40 (s, 3H, -CH_3); ^{13}C NMR (150 MHz, DMSO-d_6) δ 169.80, 164.05, 162.84, 160.63, 159.78, 151.61 (q, *J* = 32.91 Hz), 139.32, 132.67, 129.32, 122.46 (q, *J* = 272.63 Hz), 120.41, 102.88, 80.86, 60.22, 42.22, 34.28, 13.62; HRMS(ESI)m/z $[M+H]^+$ calcd for $C_{18}H_{19}N_7F_3S$: 422.13693, found: 422.13654.

2-chloro-6-(trifluoromethyl)-N-(4-(2-((6-(trifluoromethyl)pyrimidin-4-yl)amino)ethyl)phenyl)pyrimidin-4-amine (**6p**):white solid; yield 48.4%; m.p. 148.6-150.4 °C; ^{1}H NMR (600 MHz, DMSO-d_6) δ 10.54 (s, 1H, -NH-), 8.59 (s, 1H, pyrimidine-H), 8.12 (t, *J* = 5.40 Hz, 1H, -CH_2<u>NH</u>-), 7.57 (s, 2H, Ph-H), 7.31 (d, *J* = 7.98 Hz, 2H, Ph-H), 7.09 (s, 1H, pyrimidine-H), 6.87 (s, 1H, pyrimidine-H), 3.63 (q, *J* = 6.72 Hz, 2H, -<u>CH_2</u>NH-), 2.86 (t, *J* = 7.14 Hz, 2H, -CH_2-); ^{13}C NMR (150 MHz, DMSO-d_6) δ 162.84, 160.69, 159.78, 151.79 (q, *J* = 33.21 Hz), 136.45, 129.89, 121.56, 120.63, 102.88, 42.09, 34.34; HRMS(ESI)m/z $[M+Na]^+$ calcd for $C_{18}H_{13}N_6ClF_6Na$: 485.06866, found: 485.06793.

2-(methylthio)-6-(trifluoromethyl)-*N*-(4-(2-((6-(trifluoromethyl)pyrimidin-4-yl)amino)ethyl)phenyl)pyrimidin-4-amine (**6q**):yellow solid; yield 57.7%; m.p. 165.4-167.7 °C; ^{1}H NMR (600 MHz, DMSO-d_6) δ 10.13 (s, 1H, -NH-), 8.57 (s, 1H, pyrimidine-H), 8.10 (s, 1H, -CH_2<u>NH</u>-), 7.59 (s, 2H, Ph-H), 7.26 (d, *J* = 7.80 Hz, 2H, Ph-H), 6.86 (s, 1H, pyrimidine-H), 6.79 (s, 1H, pyrimidine-H), 3.62 (q, *J* = 6.48 Hz, 2H, -<u>CH_2</u>NH-), 2.84 (t, *J* = 6.78 Hz, 2H, -CH_2-), 2.49 (s, 3H, -CH_3); ^{13}C NMR (150 MHz, DMSO-d_6) δ 173.06, 162.82, 159.74,151.83 (q, *J* = 33.44 Hz), 137.19, 129.60, 122.43 (q, *J* = 272.51 Hz), 122.02 (q, *J* = 272.67 Hz), 120.91, 102.83, 42.04, 34.31, 14.06; HRMS(ESI)m/z $[M+H]^+$ calcd for $C_{19}H_{17}N_6F_6S$: 475.11341, found: 475.11356.

6-chloro-5-fluoro-N-(4-(2-((6-(trifluoromethyl)pyrimidin-4-yl)amino)ethyl)phenyl)pyrimidin-4-amine (**6R**): yellow solid; yield 41.4%; m.p. 175.2-177.1 °C; ^{1}H NMR (600 MHz, DMSO-d_6) δ 9.84 (s, 1H, -NH-), 8.58 (s, 1H, pyrimidine-H), 8.25 (s, 1H, pyrimidine-H), 8.12 (t, *J* = 5.22 Hz, 1H, -CH_2NH-), 7.63 (d, *J* = 8.22 Hz, 2H, Ph-H), 7.24 (d, *J* = 8.22 Hz, 2H, Ph-H), 6.86 (s, 1H, pyrimidine-H), 6.46 (d, *J* = 5.82 Hz, 1H, pyrimidine-H), 3.63 (q, *J* = 6.72 Hz, 2H, -CH_2NH-), 2.85 (t, *J* = 7.14 Hz, 2H, -CH_2-); 170.80, 162.82, 159.77, 153.10(d, *J* = 11.13 Hz), 151.60 (q, *J* = 33.87 Hz), 150.94 (d, *J* = 9.33 Hz), 142.41 (d, *J*= 13.91 Hz), 141.60, 139.85, 136.84, 135.35, 129.29, 122.44 (q, *J* = 272.82 Hz), 122.27, 102.85, 42.09, 34.33; HRMS(ESI)m/z $[M+H]^+$ calcd for $C_{17}H_{16}N_6ClF_4$: 413.08991, found: 413.08962.

6-ethyl-5-fluoro-*N*-(4-(2-((6-(trifluoromethyl)pyrimidin-4-yl)amino)ethyl)phenyl)pyrimidin-4-amine (**6s**):gray solid;yield 43.5%; m.p.208.7-211.5 °C;^{1}H NMR (600 MHz, DMSO-d_6) δ 9.39 (s, 1H, -NH-), 8.59 (s, 1H, pyrimidine-H), 8.32 (d, *J* = 1.56 Hz, pyrimidine-H), 8.11 (t,*J* = 5.34 Hz, 1H, -CH_2NH-), 7.68 (d, *J* = 8.40 Hz, 2H, Ph-H), 7.22 (d, *J* = 8.28 Hz,2H,Ph-H), 6.87 (s, 1H, pyrimidine-H), 3.63 (q, *J* = 6.72 Hz, 2H, -CH_2NH-), 2.84 (t, *J* = 7.14 Hz, 2H, -CH_2-), 2.73-2.69 (m, 2H, -CH_2), 1.23 (t, *J* = 7.62 Hz, 2H, -CH_2-); ^{13}C NMR (150 MHz, DMSO-d_6) δ 162.84, 159.78, 153.33 (d, *J* = 11.19 Hz), 153.03 (d, *J* = 10.47 Hz), 149.91 (d, *J* = 22.91 Hz), 144.10, 142.39, 137.59, 134.41, 129.19, 124.29 (q, *J*= 275.58 Hz), 121.70, 102.86, 42.15, 34.33, 23.52, 12.27; HRMS(ESI)m/z $[M+H]^+$ calcd for $C_{19}H_{19}N_6F_4$: 407.16018, found: 407.16010.

2,6-dimethyl-*N*-(4-(2-((6-(trifluoromethyl)pyrimidin-4-yl)amino)ethyl)phenyl)pyrimidin-4-amine (**6t**): white solid; yield 39.5%; m.p. 128.1-129.8 °C;^{1}H NMR (600 MHz, DMSO-d_6) δ 9.30 (s, 1H, -NH-), 8.58 (s, 1H, pyrimidine-H), 8.11 (t, *J* = 5.34 Hz, 1H, -CH_2NH-), 7.56 (d, *J* = 8.16 Hz, 2H, Ph-H), 7.19 (d, *J* = 8.28 Hz, 2H, Ph-H), 6.87 (s, 1H, pyrimidine-H), 6.41 (s, 1H, pyrimidine-H), 3.61 (q, *J* = 6.78 Hz, 2H, -CH_2NH-), 2.81 (t, *J* = 7.20 Hz, 2H, -CH_2-), 2.38 (s, 2H, -CH_3), 2.23 (s, 3H, -CH_3). ^{13}C NMR (150 MHz, DMSO-d_6) δ 167.43, 166.57, 164.86, 162.82, 161.10, 159.77, 151.82 (q, *J* = 33.93 Hz), 138.74, 133.34, 132.16, 131.97, 129.45, 129.12, 124.26 (q, *J* = 272.21 Hz), 120.46, 102.86 (d, *J* = 2.88 Hz), 101.99, 42.19, 34.30, 26.16, 23.94; HRMS(ESI)m/z $[M+H]^+$ calcd for $C_{19}H_{20}N_6F_3$:389.16961, found: 389.16916.

2、 ^{1}H NMR, ^{13}C NMR and HRMS Spectra (Figure S1-S60)

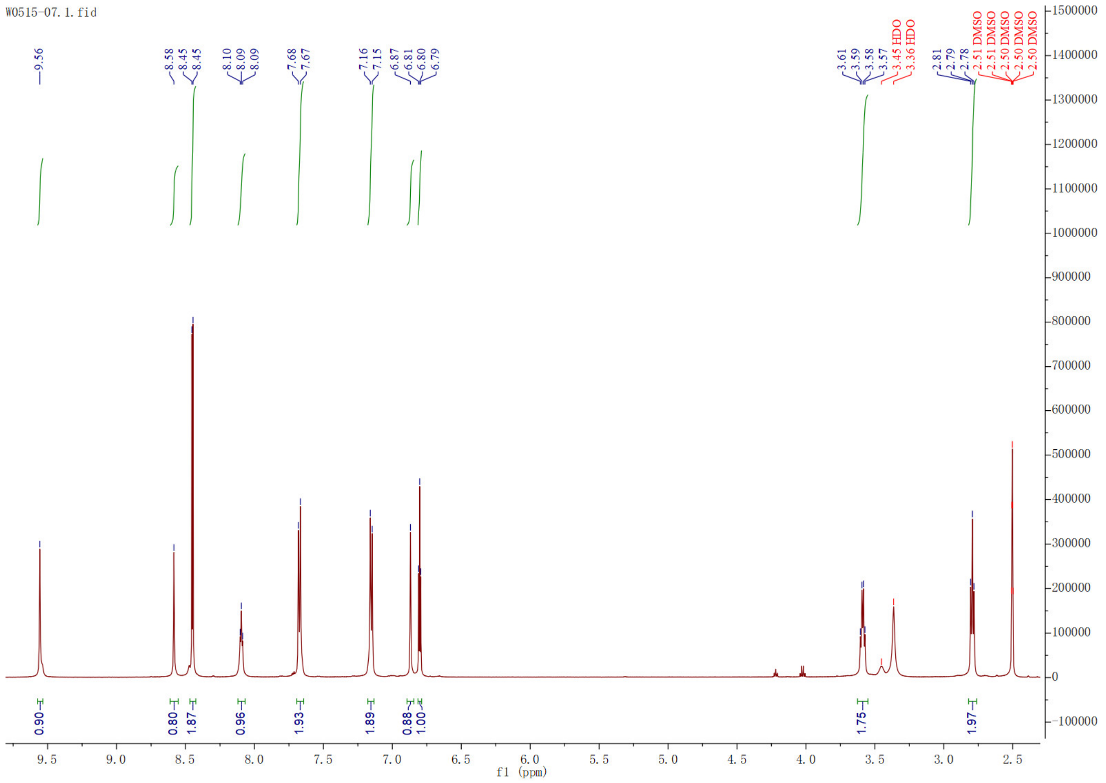

Figure S1. ^{1}H NMR Spectrum of of 6a.

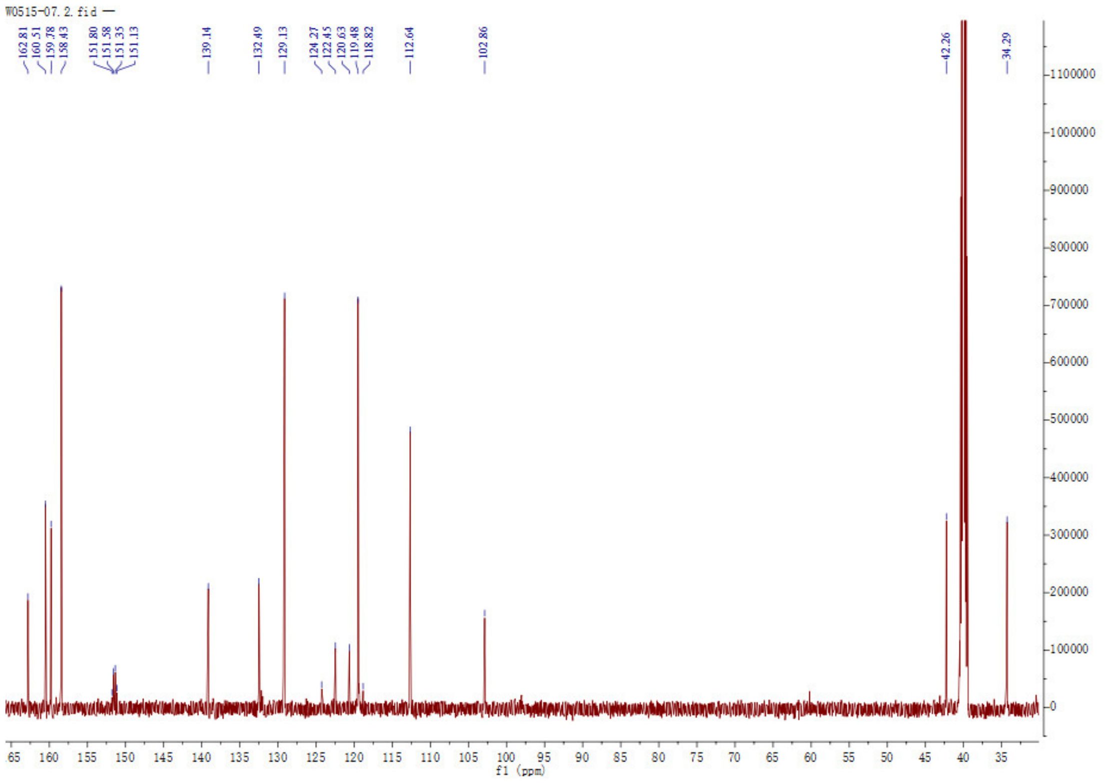

Figure S2. ^{13}C NMR Spectrum of 6a.

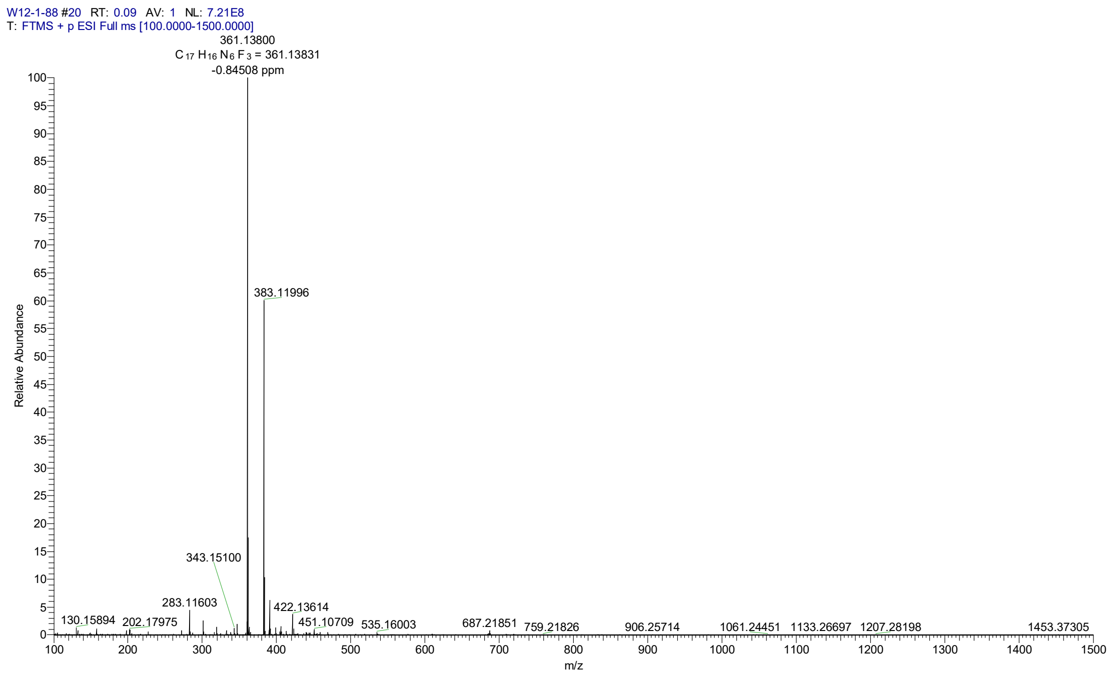

Figure S3. HRMS Spectrum of 6a.

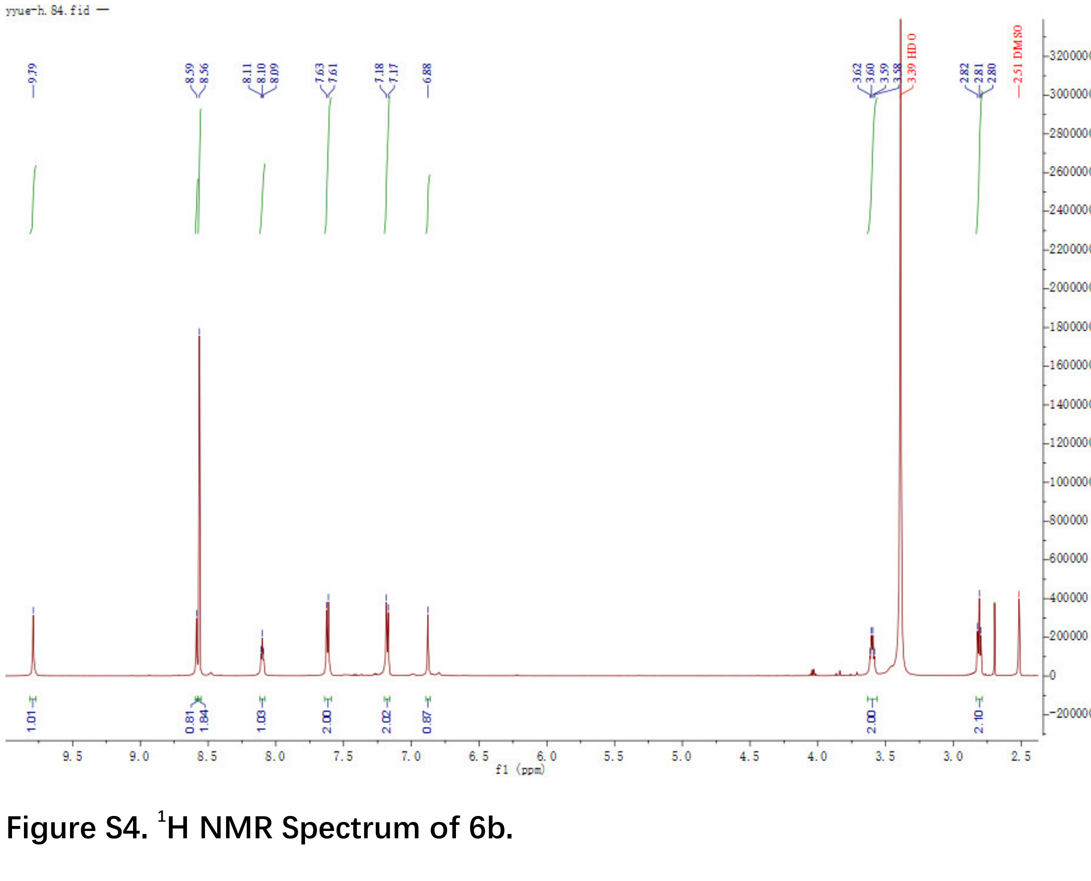

Figure S4. ^{1}H NMR Spectrum of 6b.

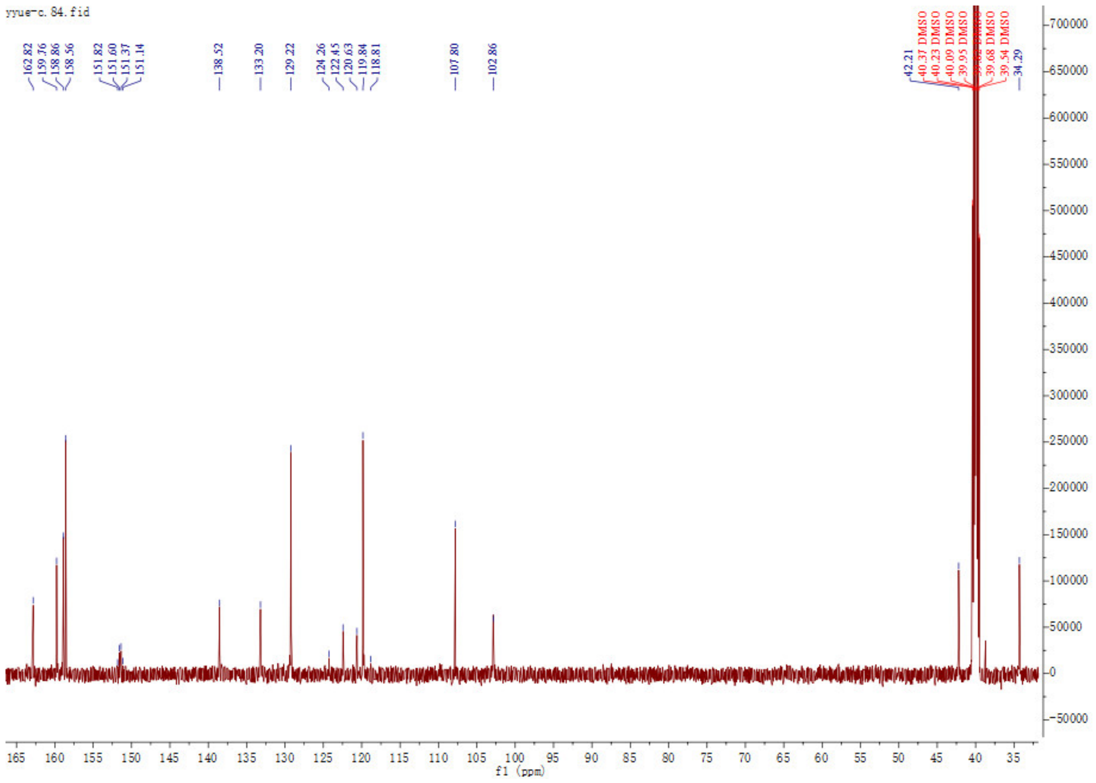

Figure S5. ^{13}C NMR Spectrum of 6b.

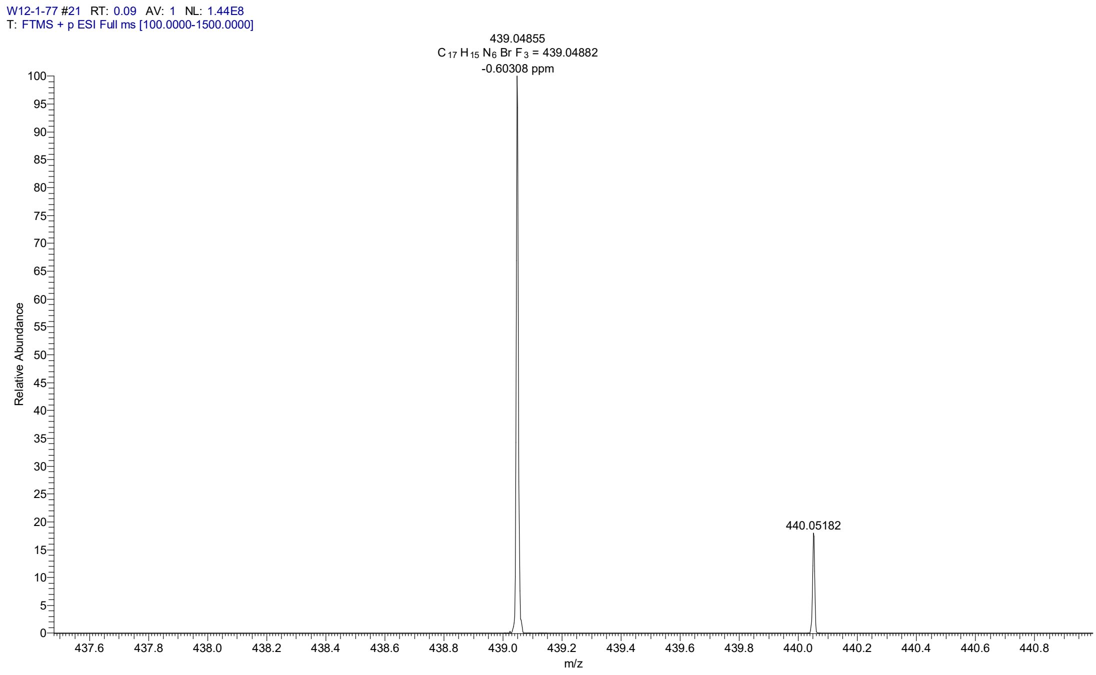

Figure S6. HRMS Spectrum of 6b.

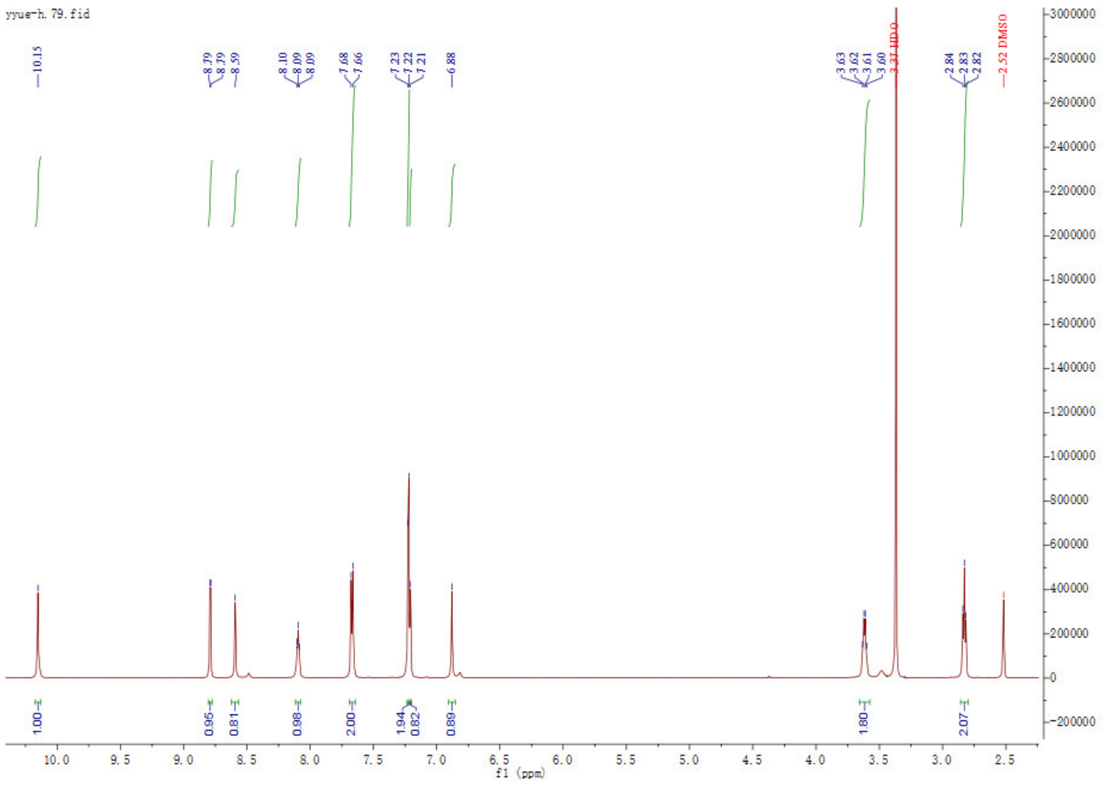

Figure S7. ^{1}H NMR Spectrum of 6c.

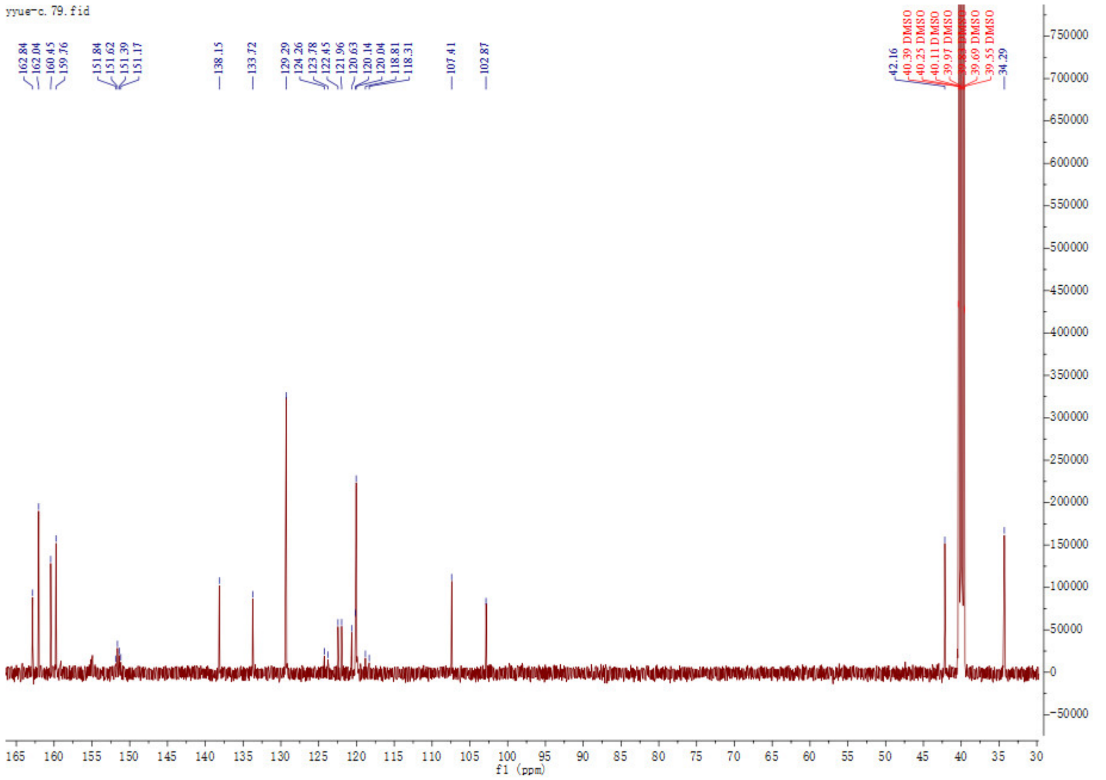

Figure S8. ^{13}C NMR Spectrum of 6c.

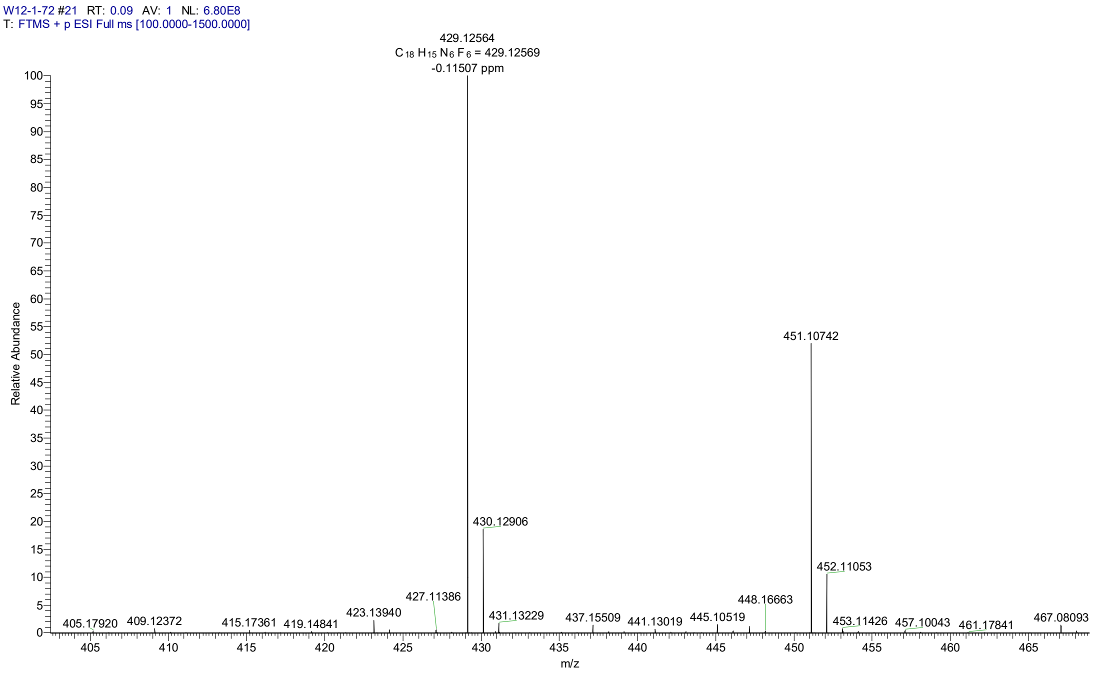

Figure S9. HRMS Spectrum of 6c.

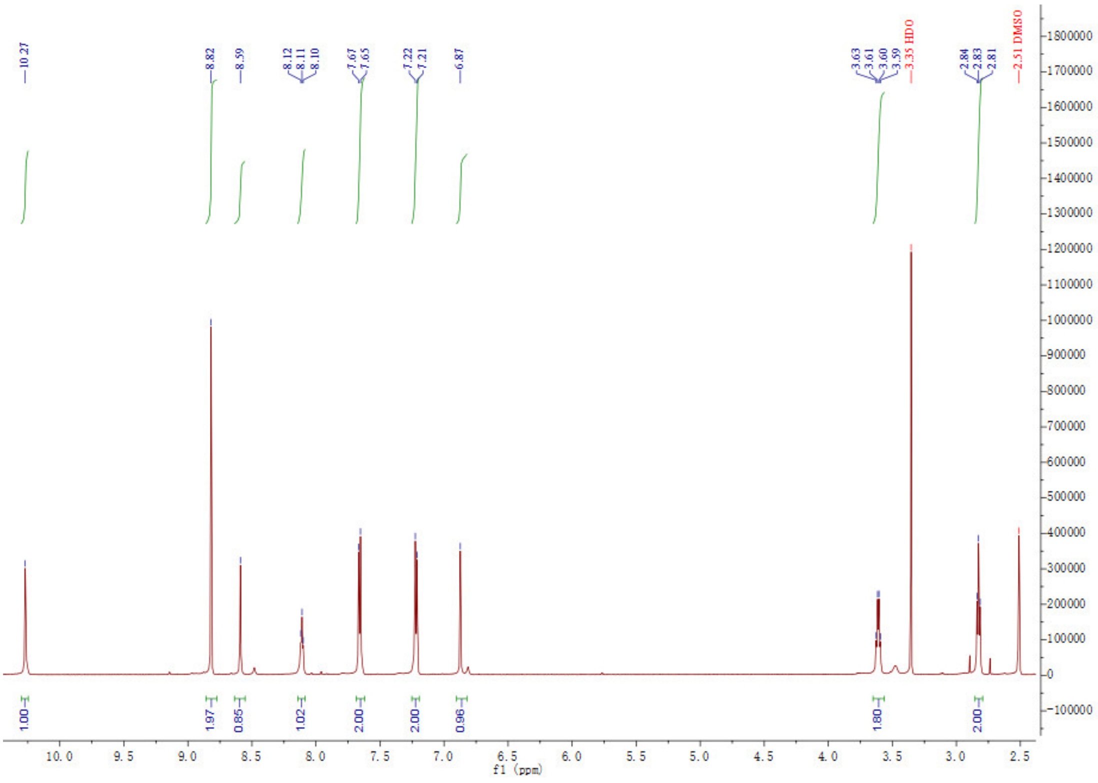

Figure S10. ^{1}H NMR Spectrum of 6d.

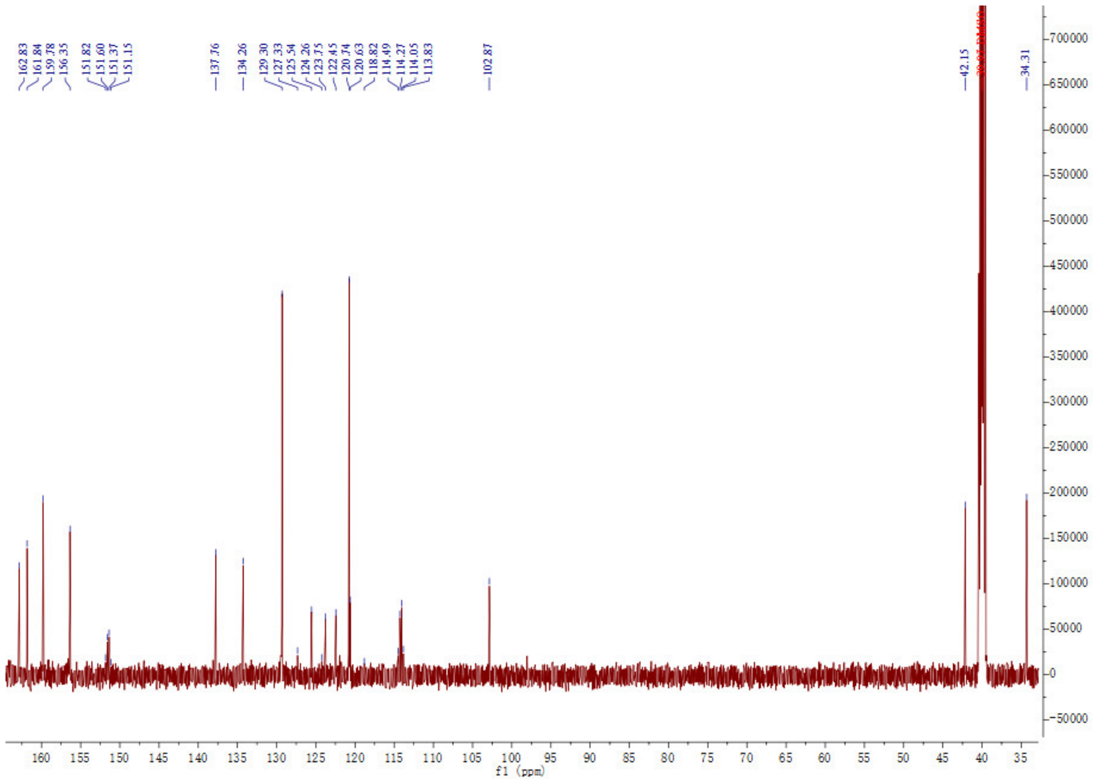

Figure S11. ^{13}C NMR Spectrum of 6d.

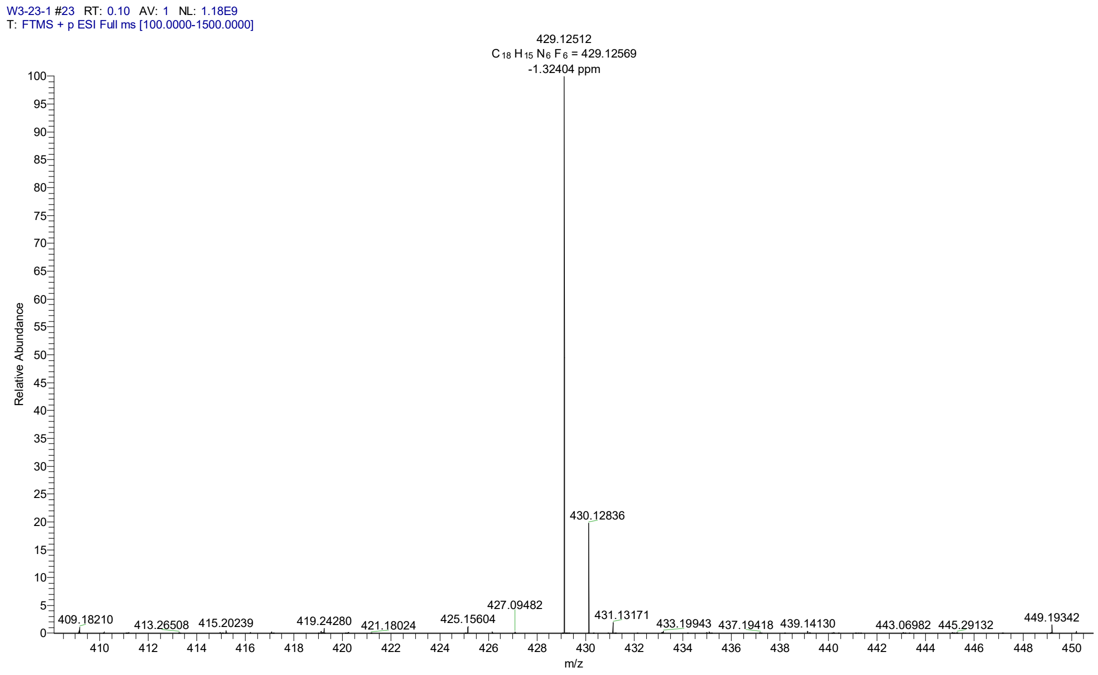

Figure S12. HRMS Spectrum of 6d.

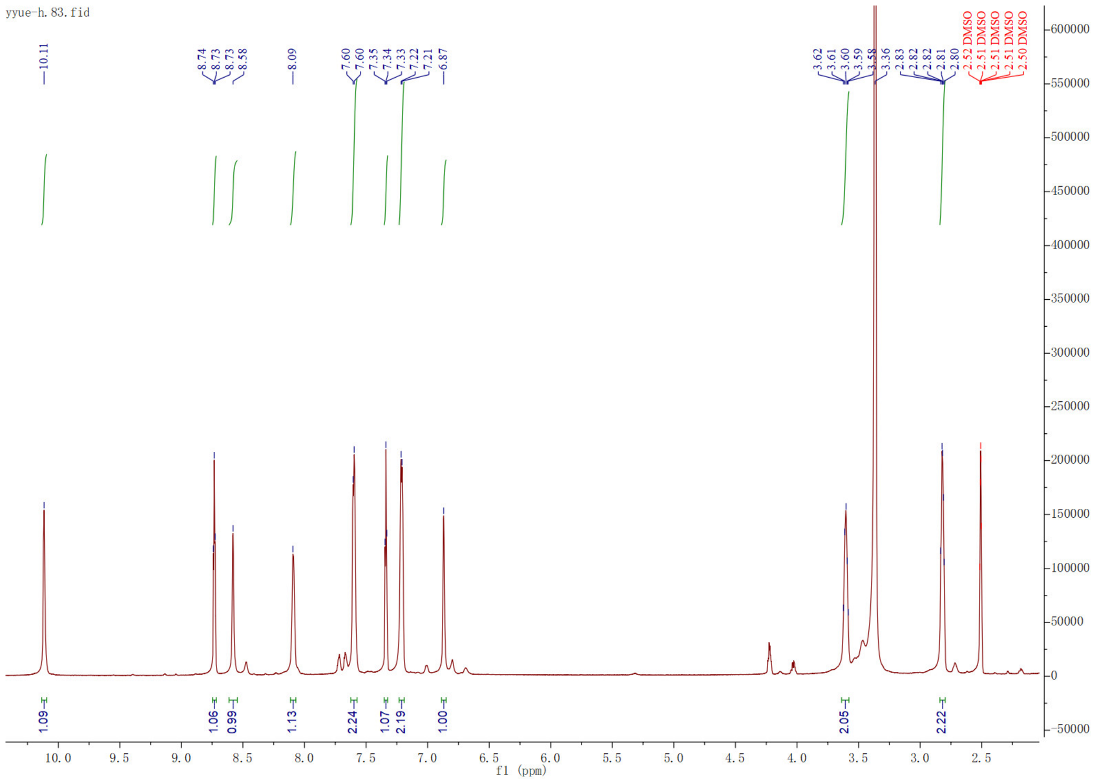

Figure S13. ¹H NMR Spectrum of 6e.

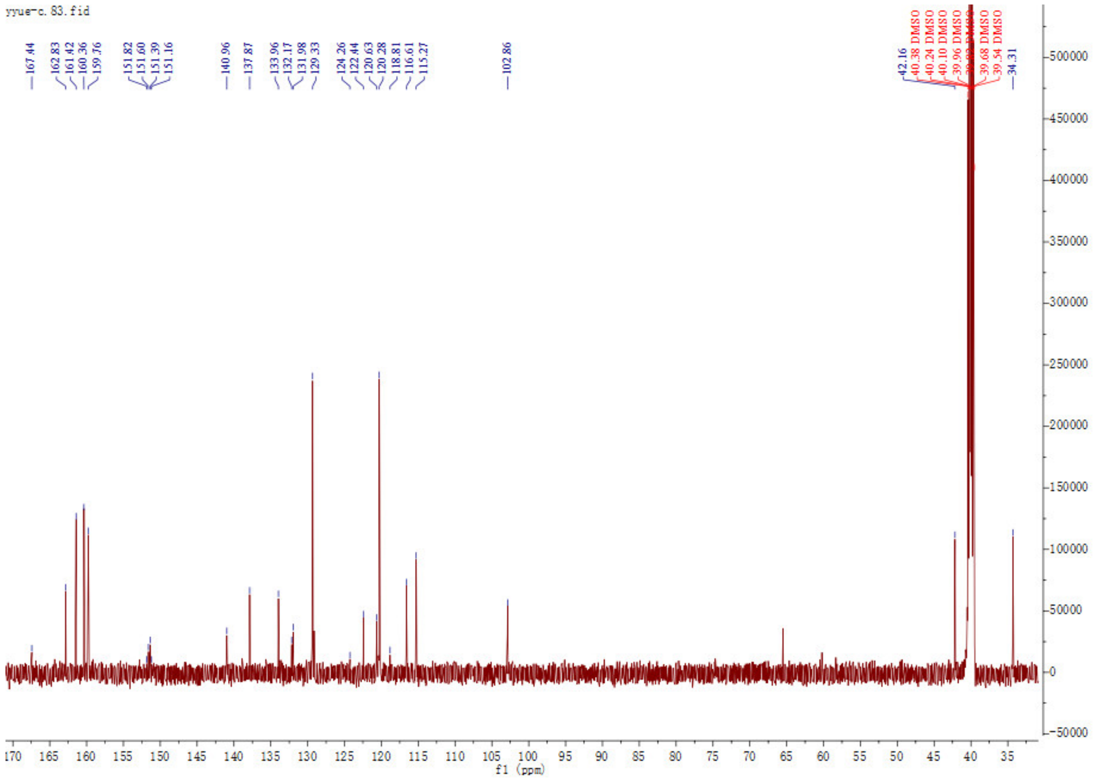

Figure S14. ¹³C NMR Spectrum of 6e.

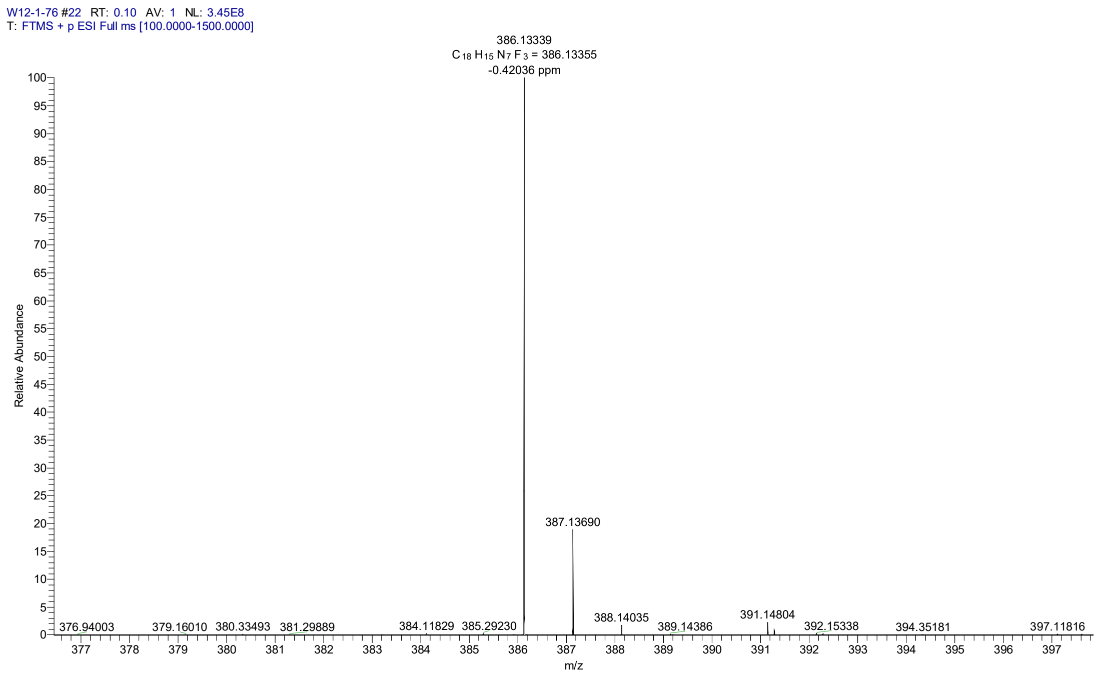

Figure S15. HRMS Spectrum of 6e.

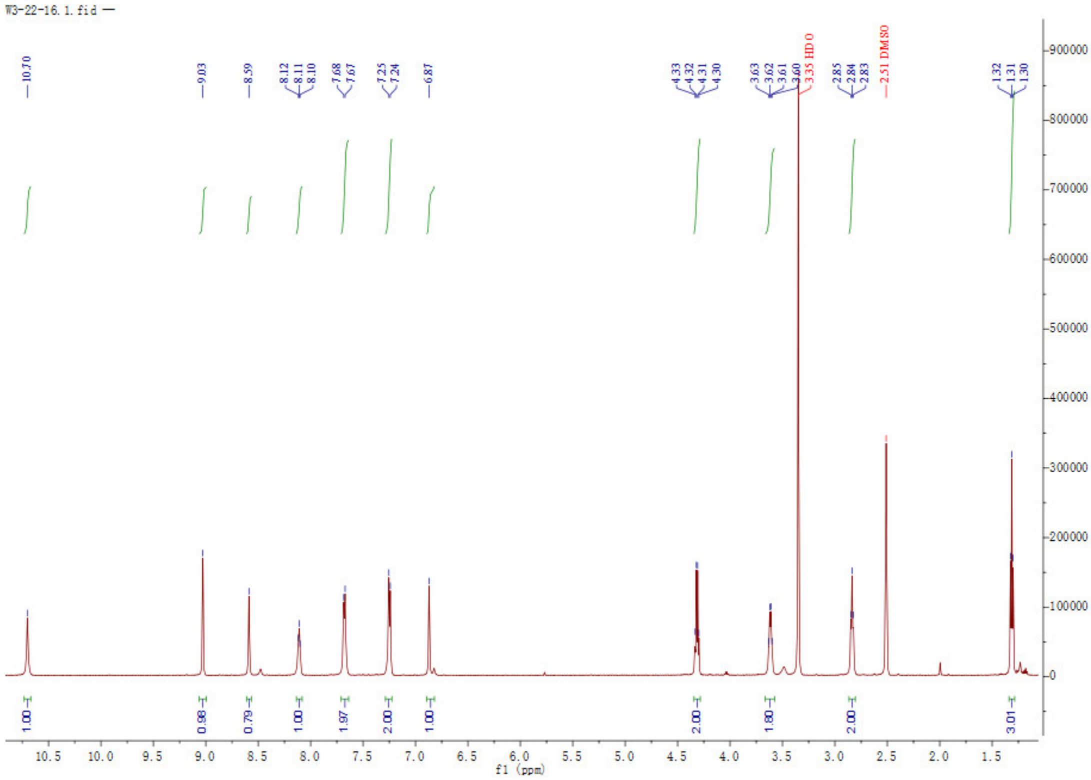

Figure S16. ^{1}H NMR Spectrum of 6f.

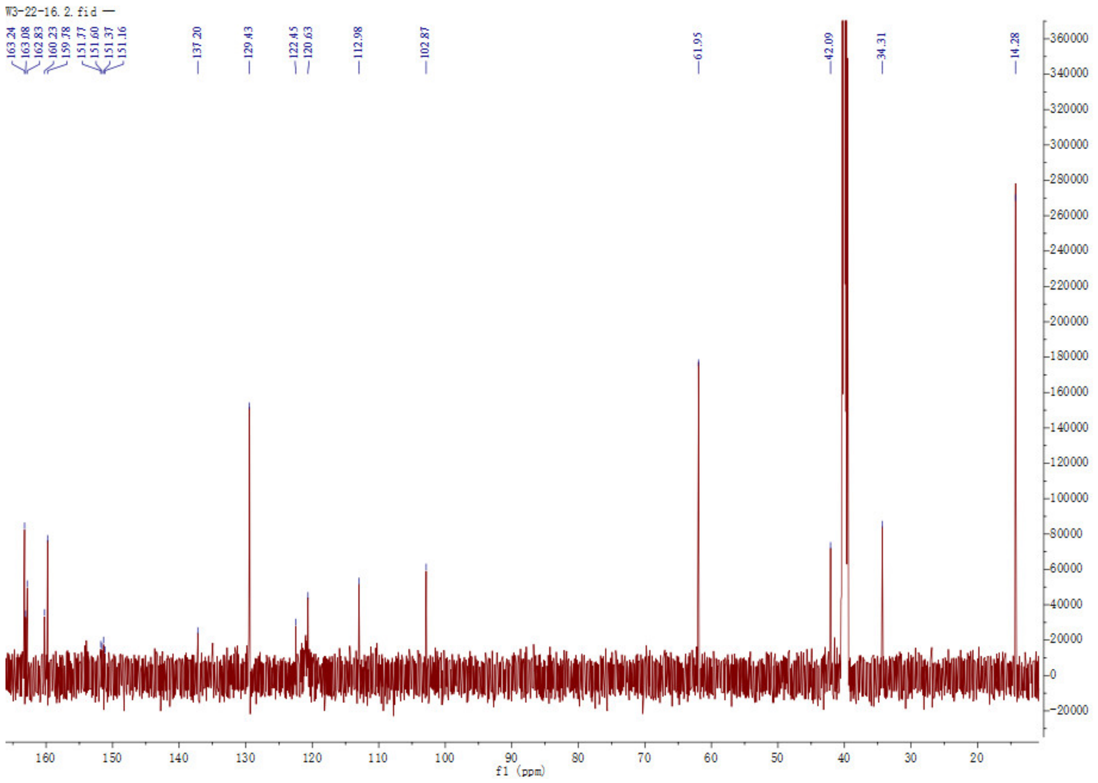

Figure S17. ^{13}C NMR Spectrum of 6f.

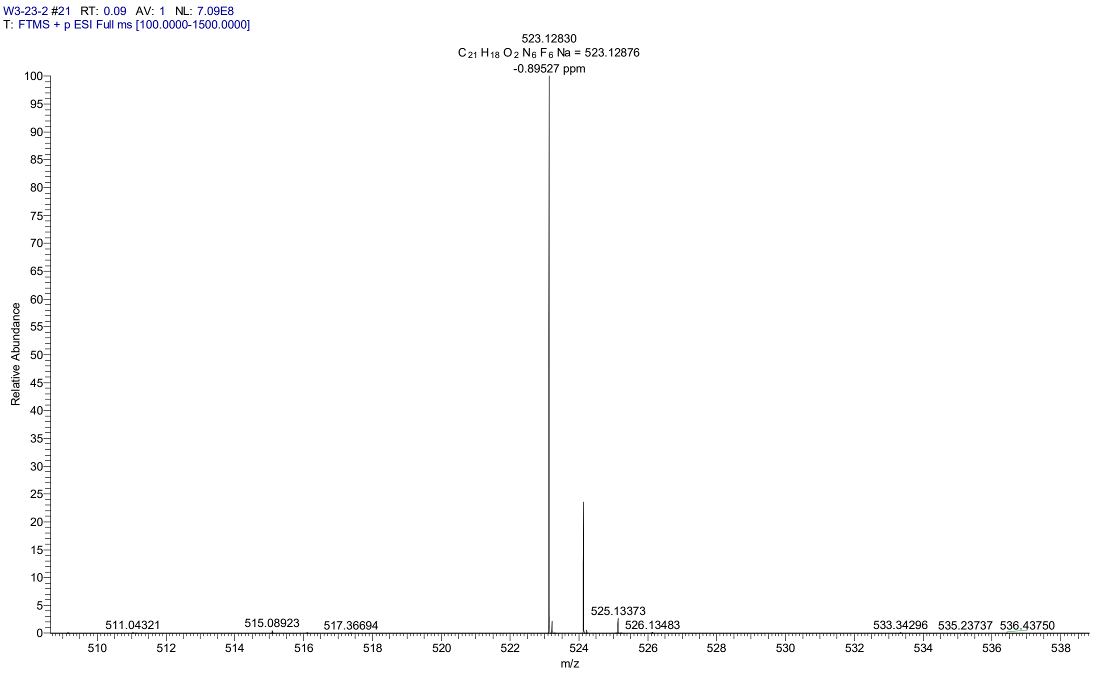

Figure S18. HRMS Spectrum of 6f.

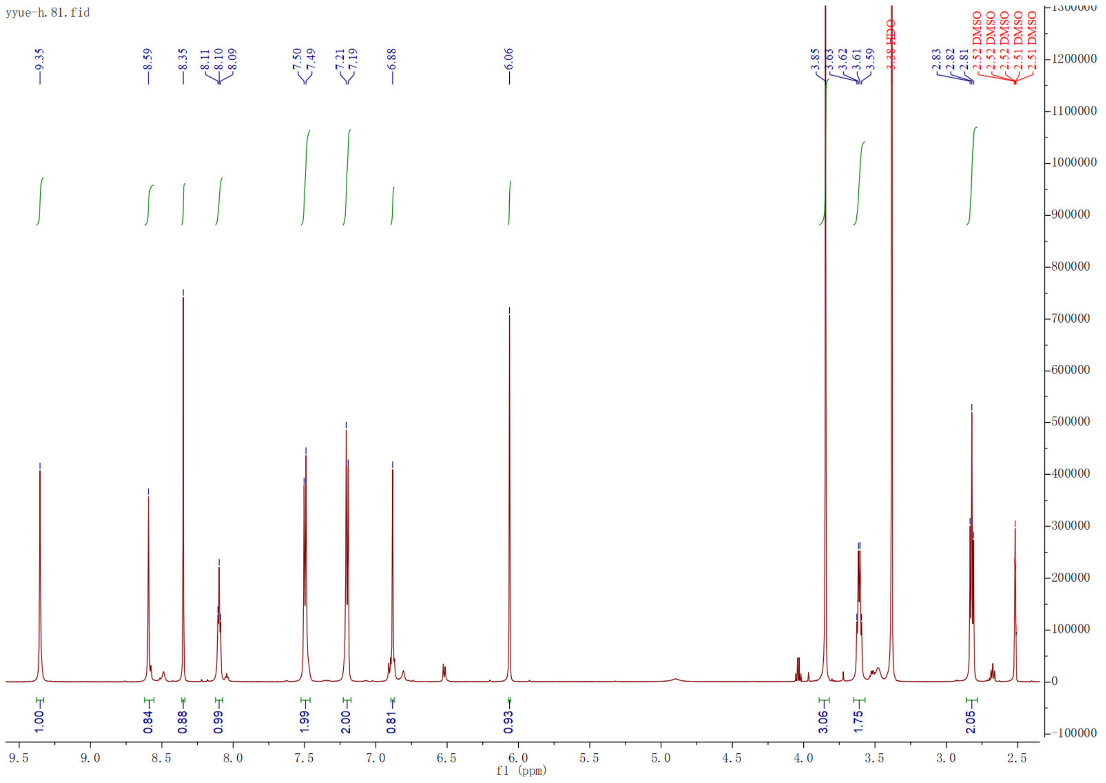

Figure S19. ^{1}H NMR Spectrum of 6g.

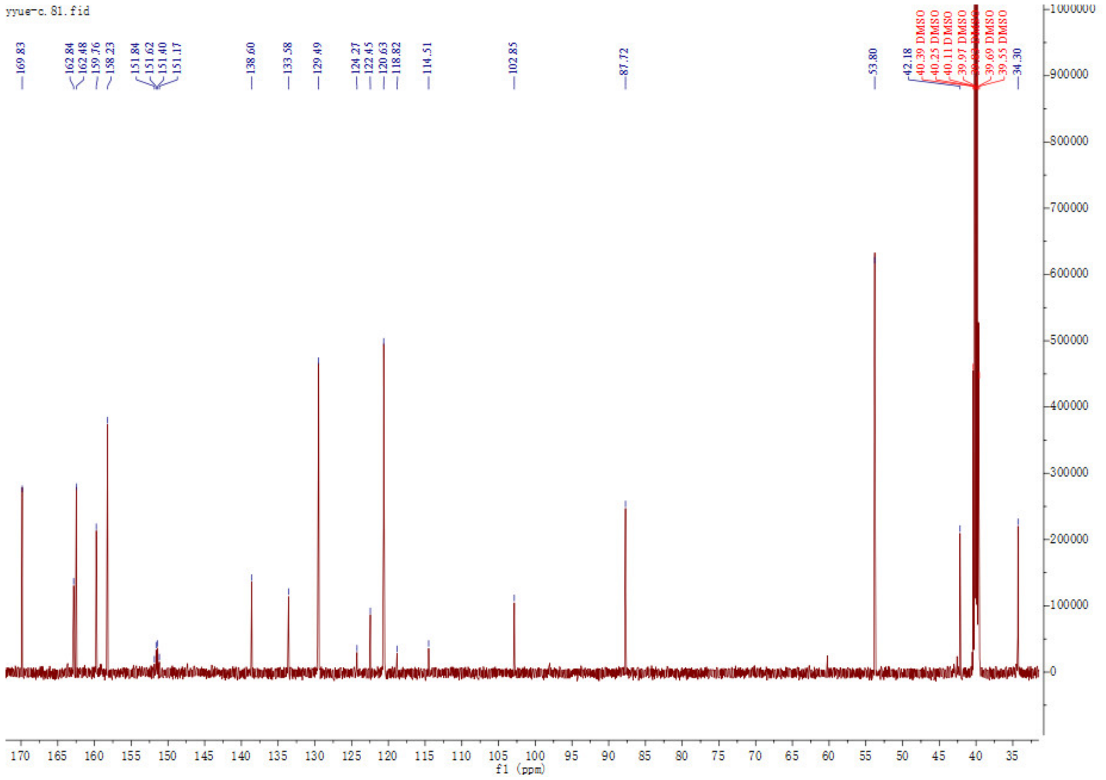

Figure S20. ^{13}C NMR Spectrum of 6g.

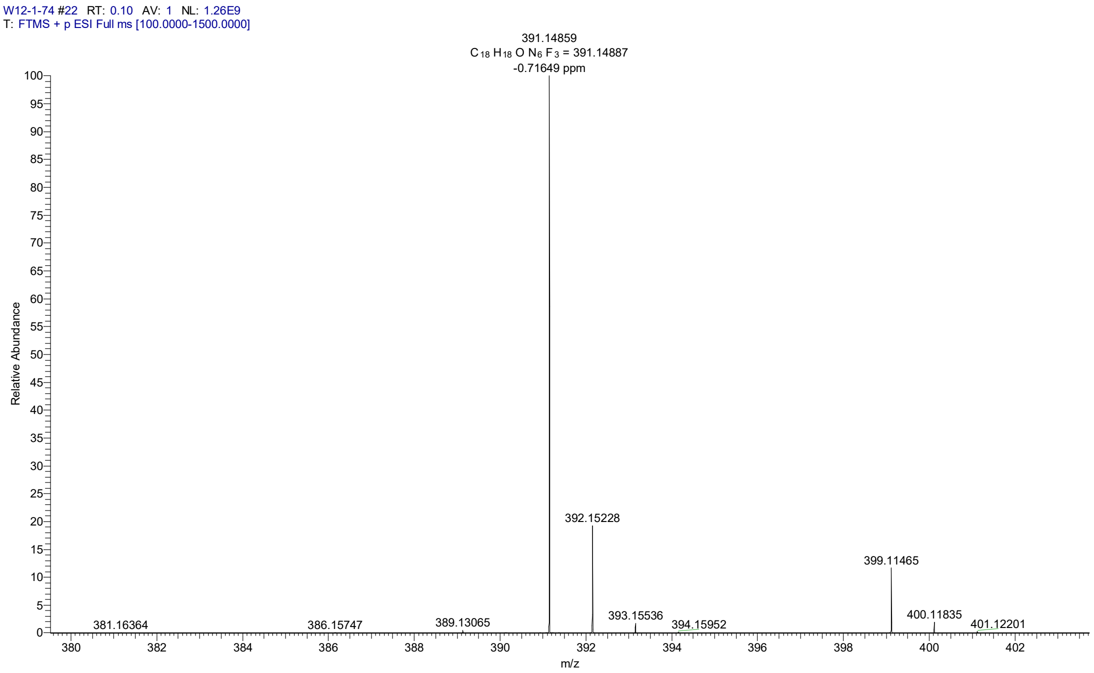

Figure S21. HRMS Spectrum of 6g.

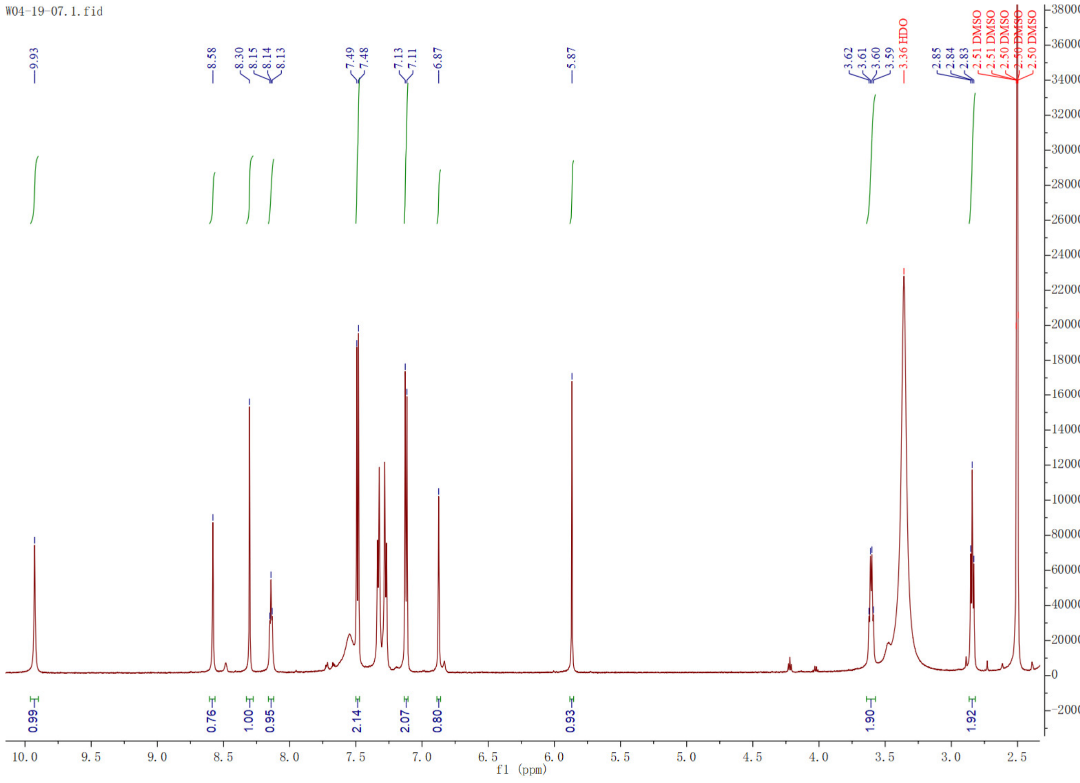

Figure S22. ^{1}H NMR Spectrum of 6h.

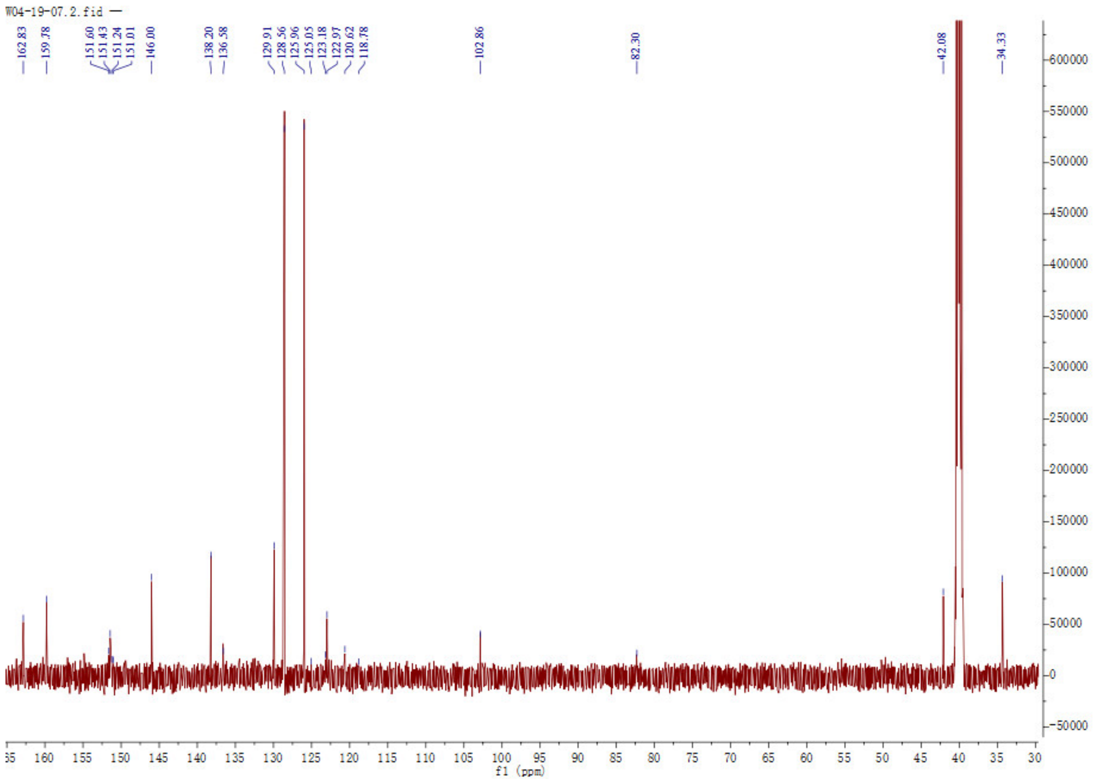

Figure S23. ^{13}**C NMR Spectrum of 6h.**

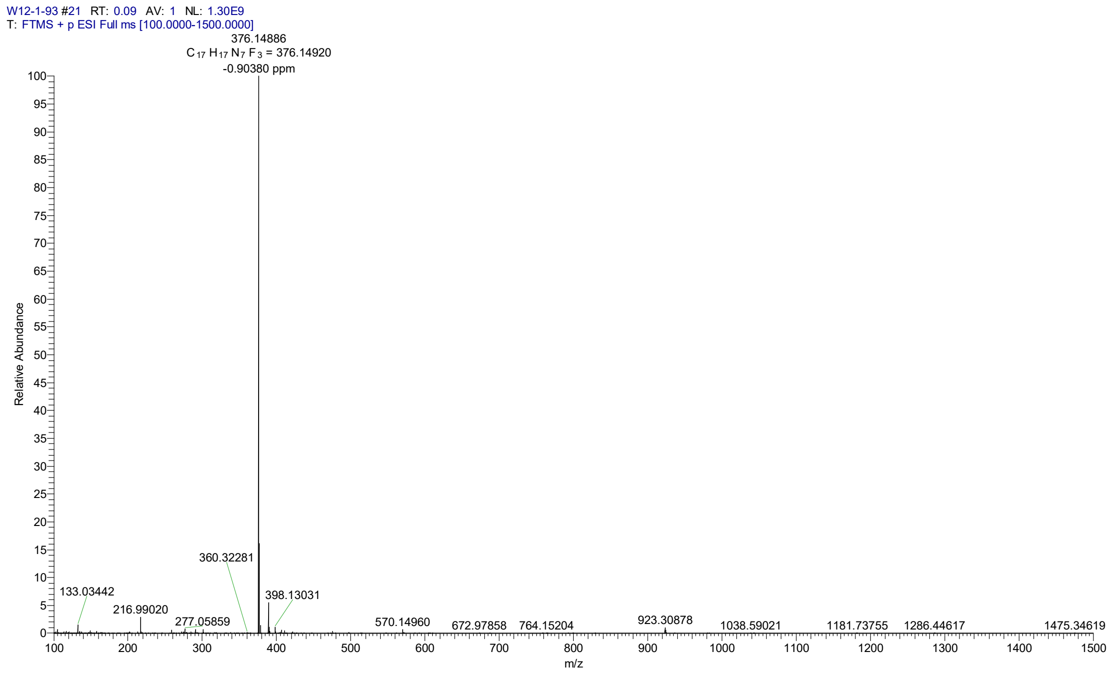

Figure S24. HRMS Spectrum of 6h.

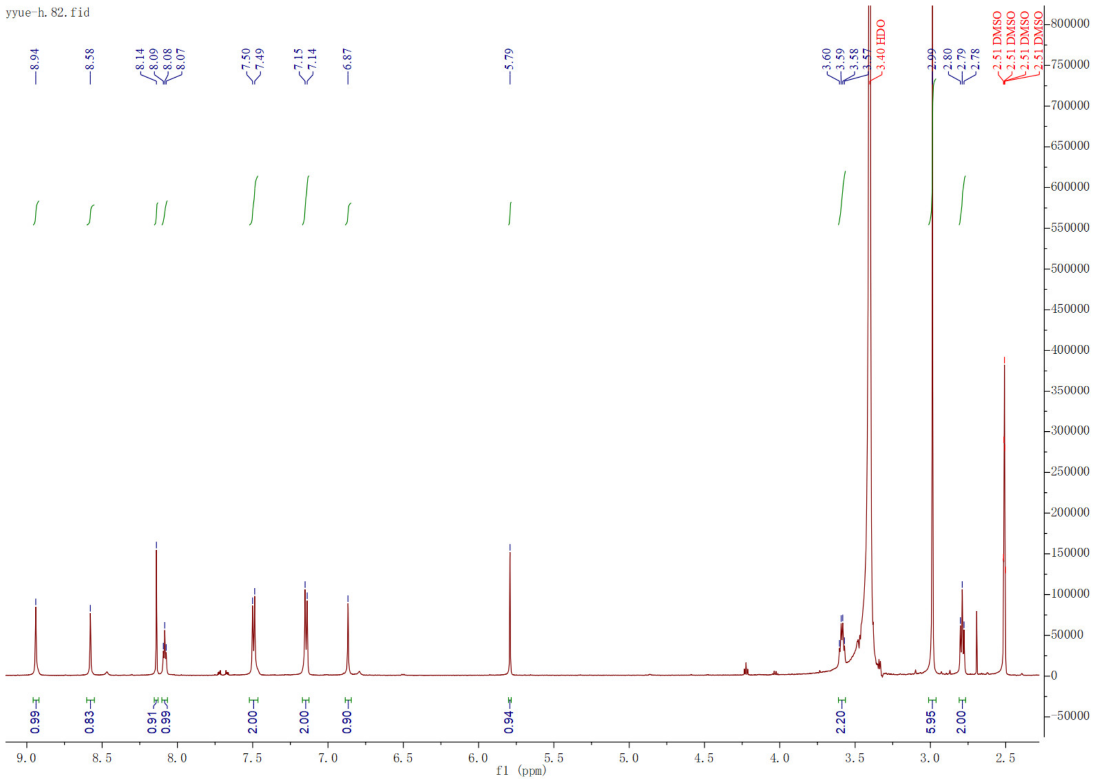

Figure S25. ¹H NMR Spectrum of 6i.

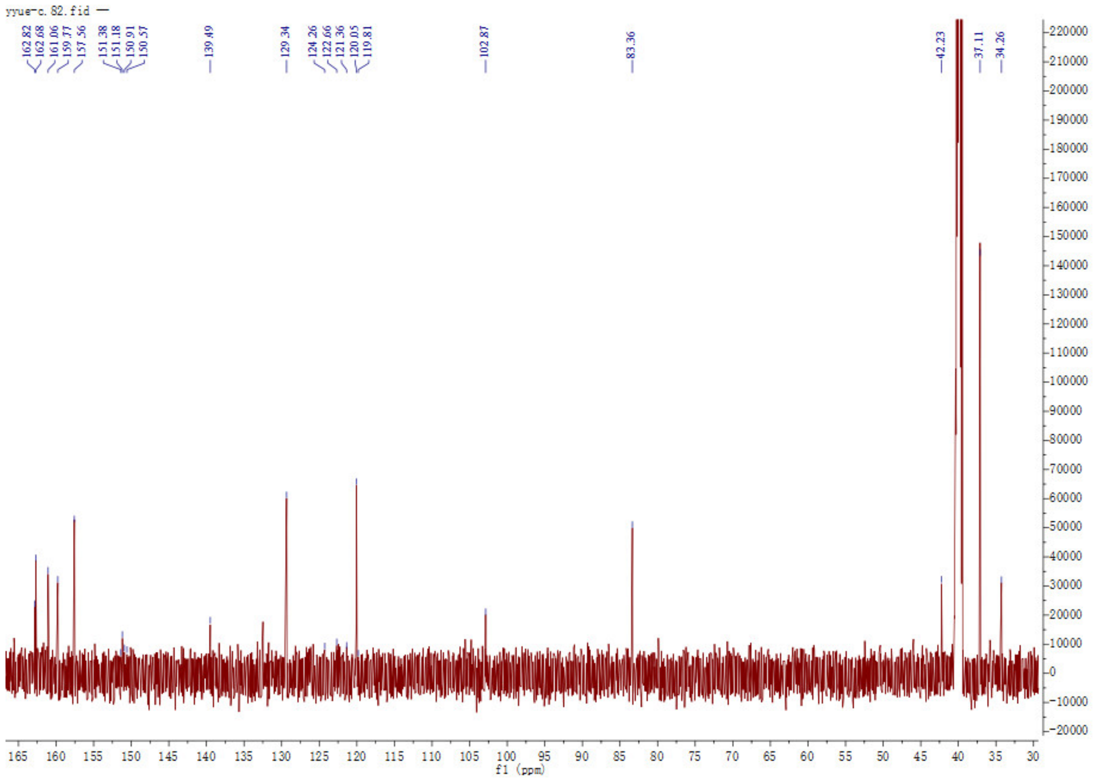

Figure S26. ¹³C NMR Spectrum of 6i.

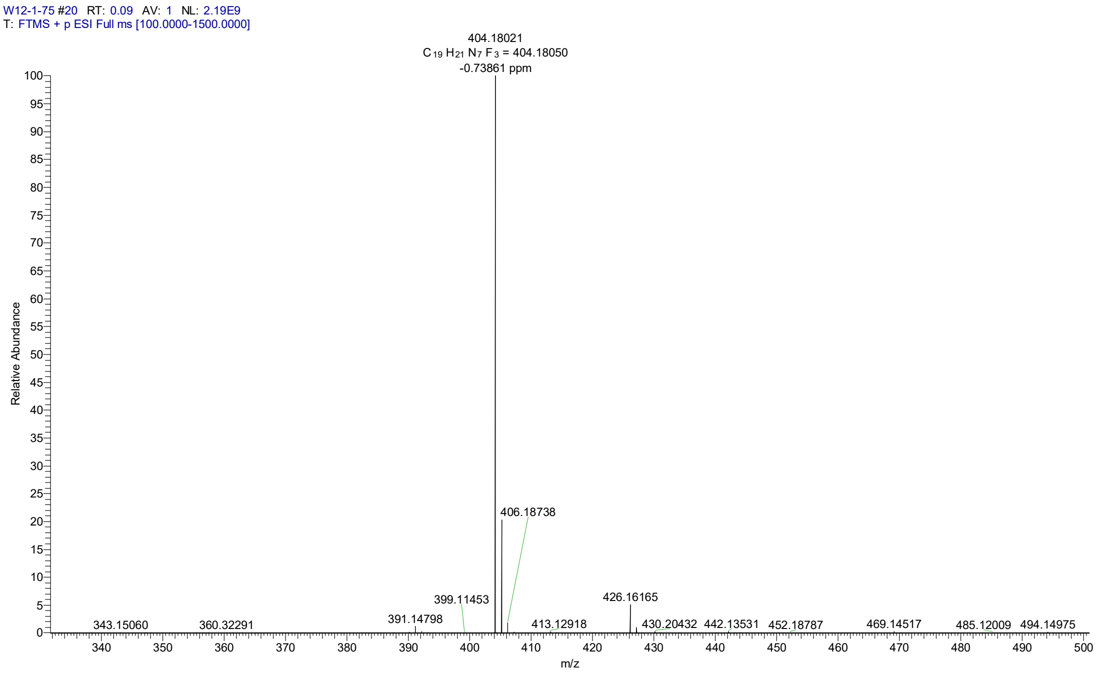

Figure S27. HRMS Spectrum of 6i.

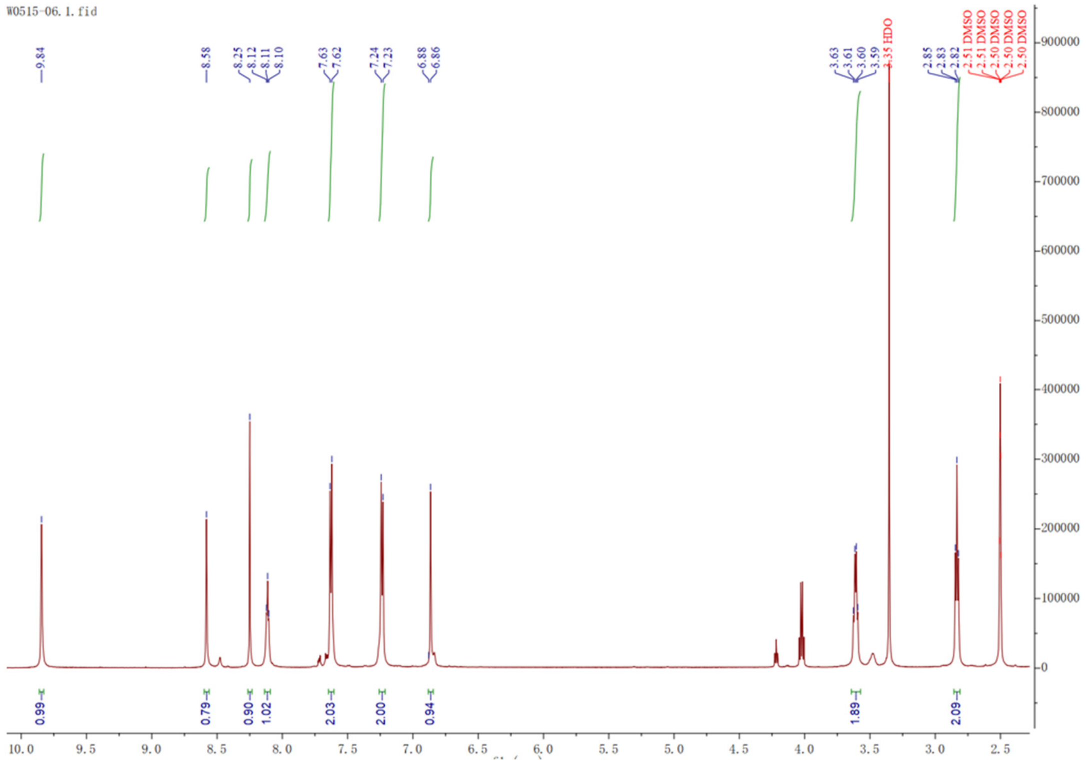

Figure S28. ^{1}H NMR Spectrum of 6j.

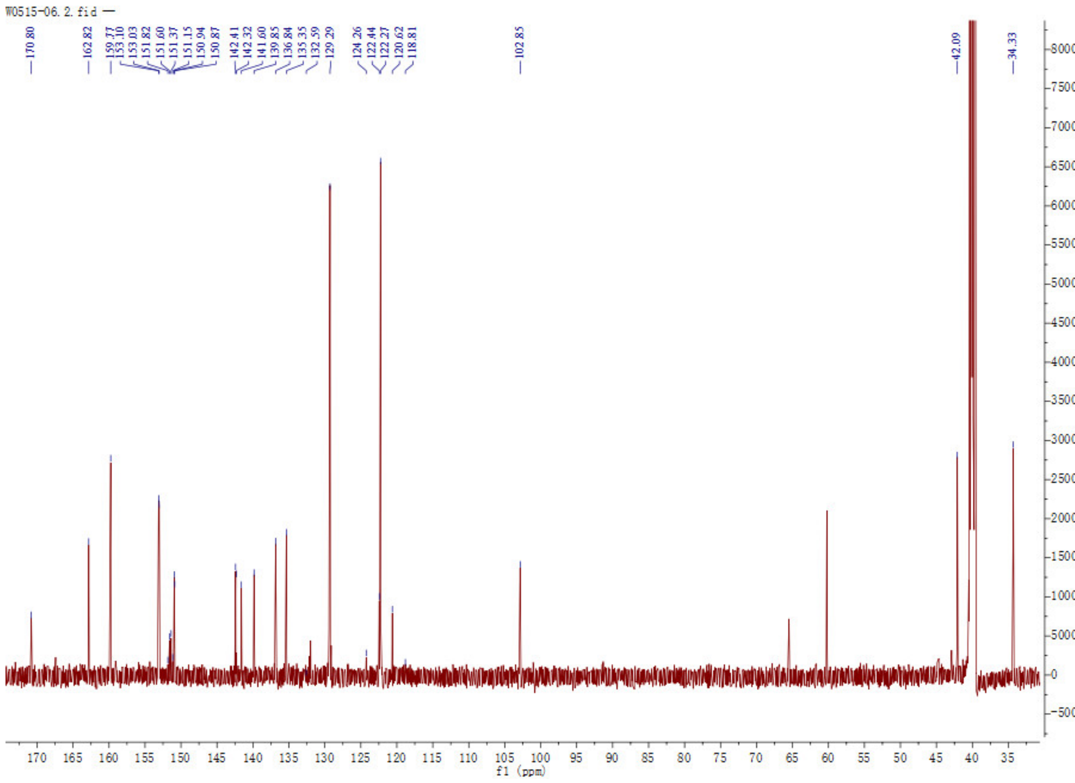

Figure S29. ^{13}C NMR Spectrum of 6j.

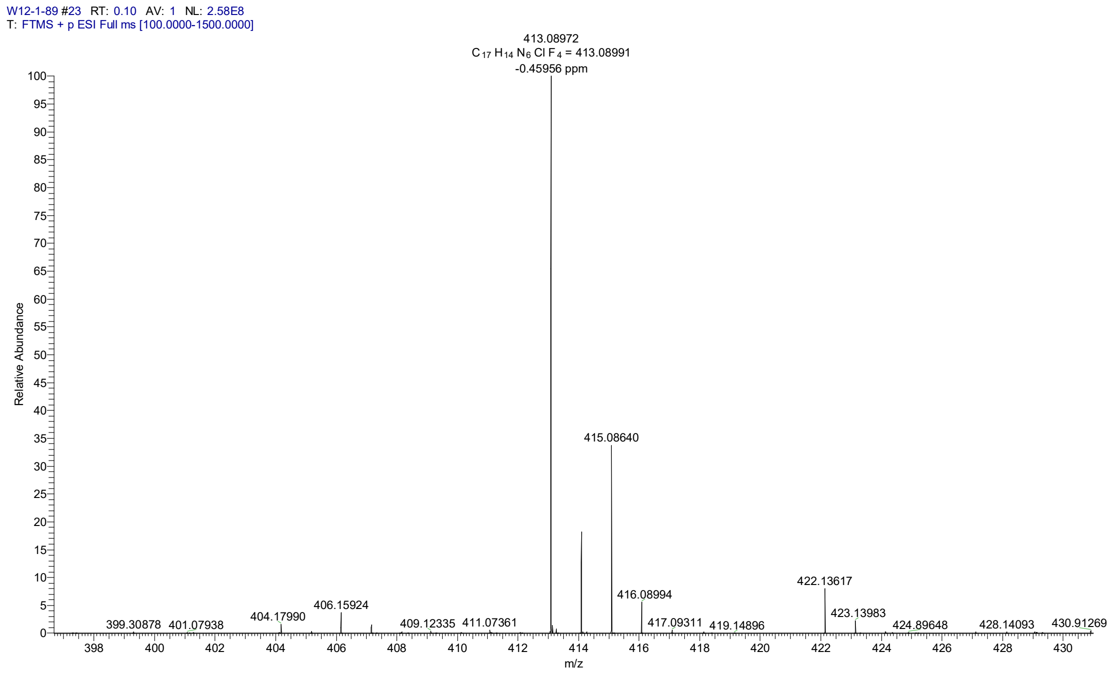

Figure S30. HRMS Spectrum of 6j.

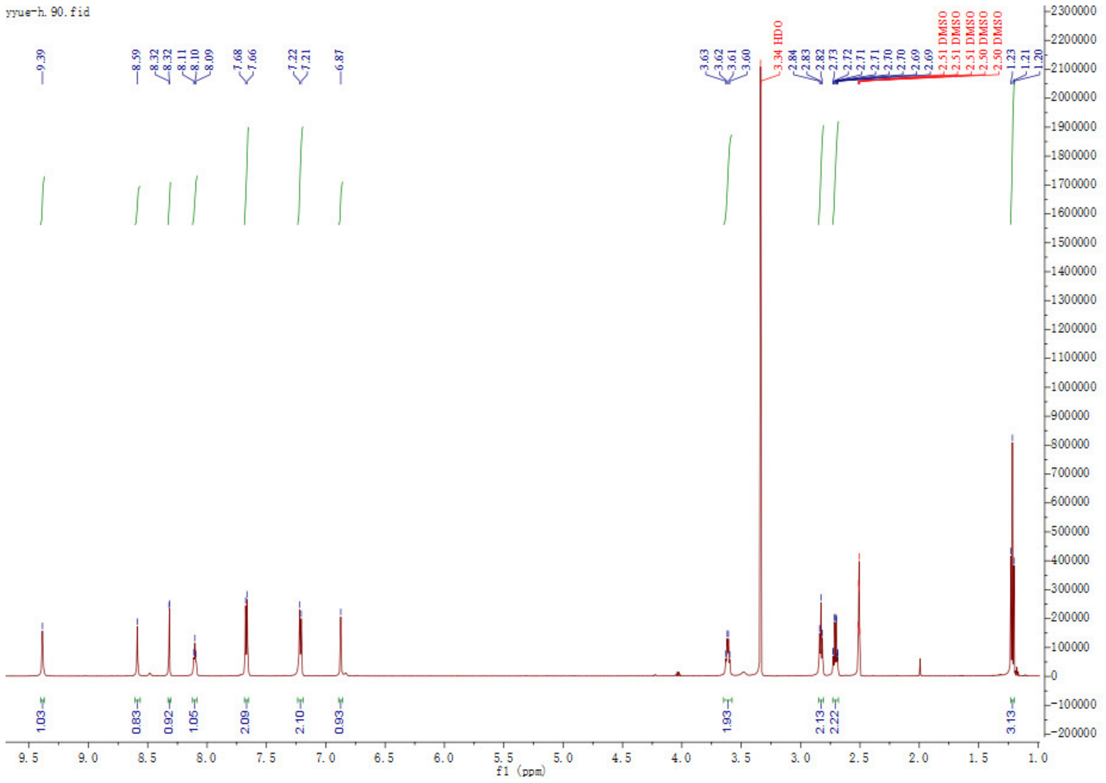

Figure S31. ^{1}H NMR Spectrum of 6k.

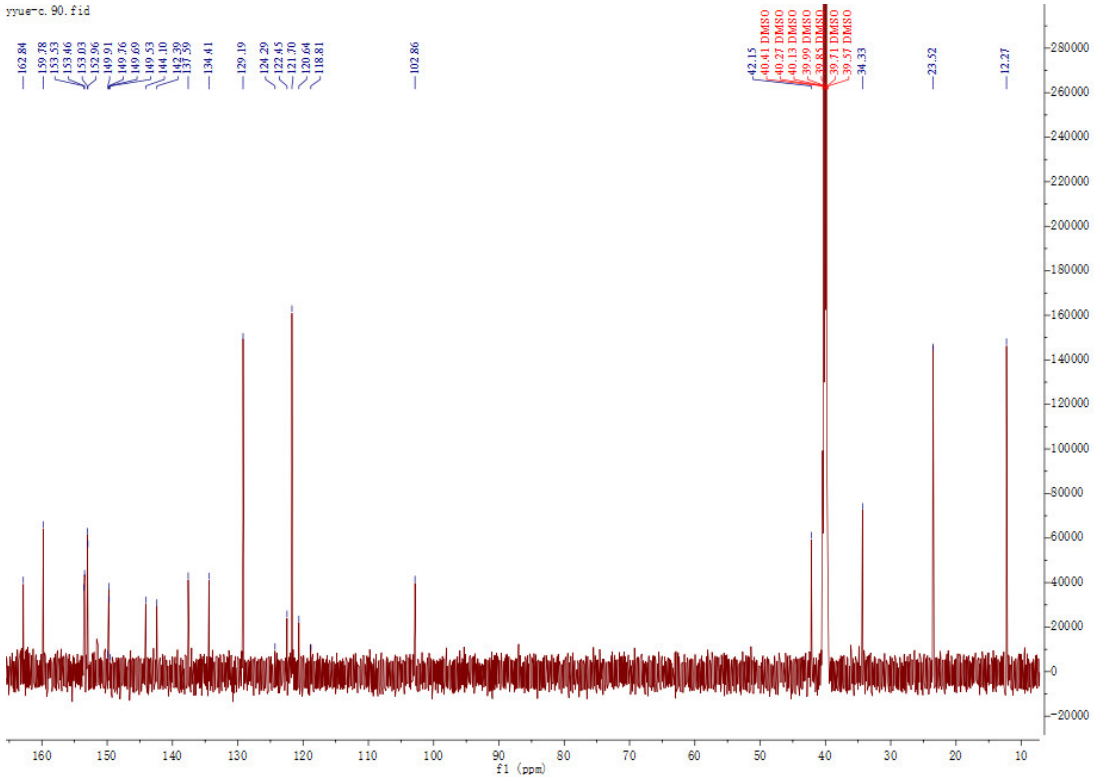

Figure S32. ^{13}C NMR Spectrum of 6k.

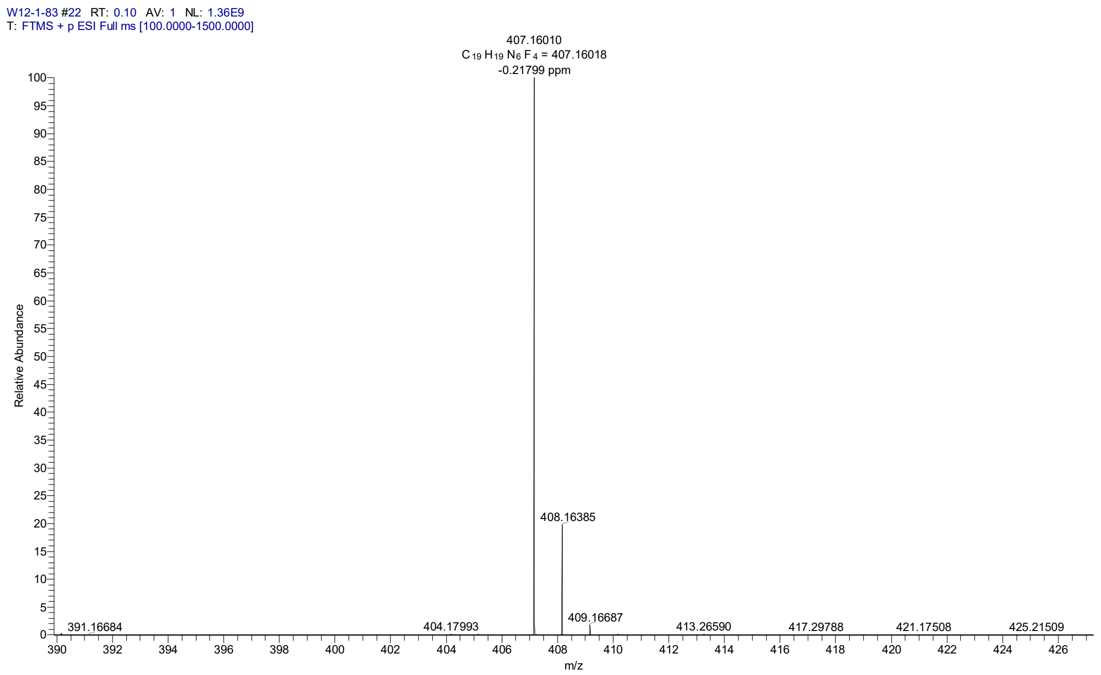

Figure S33. HRMS Spectrum of 6k.

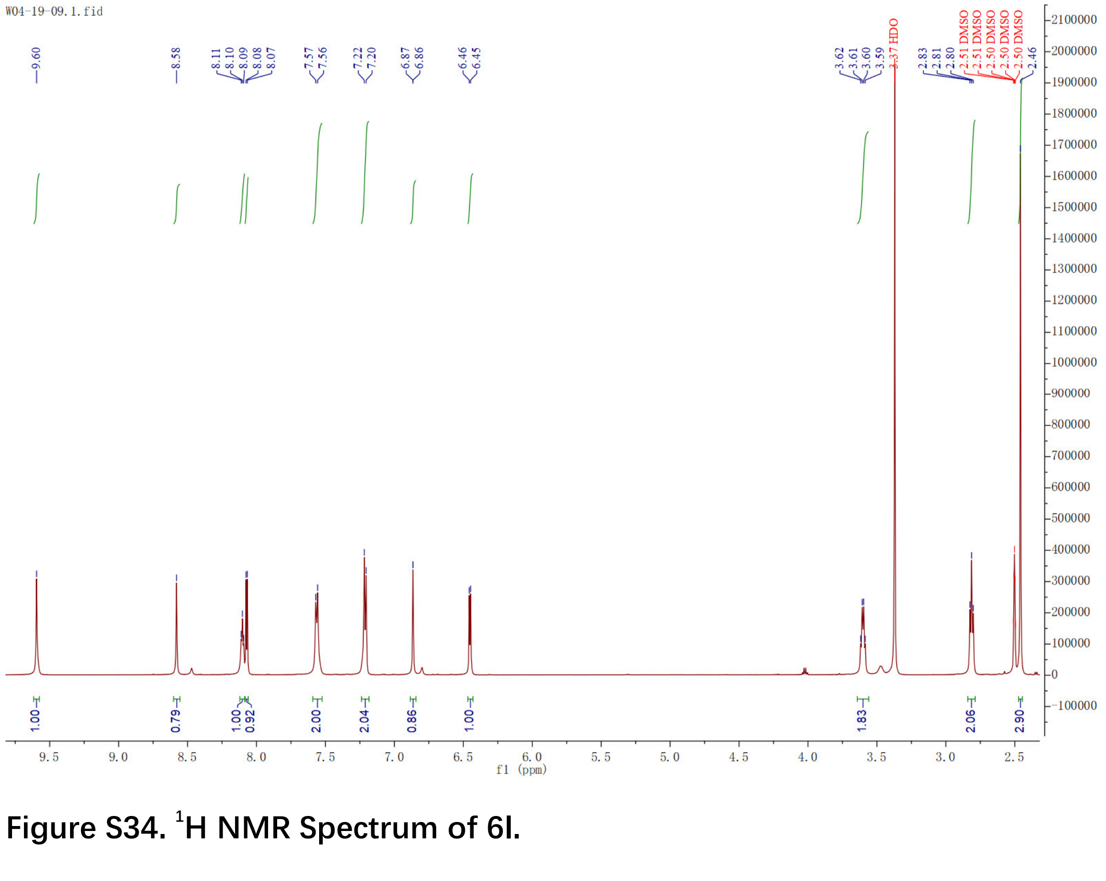

Figure S34. ^{1}H NMR Spectrum of 6l.

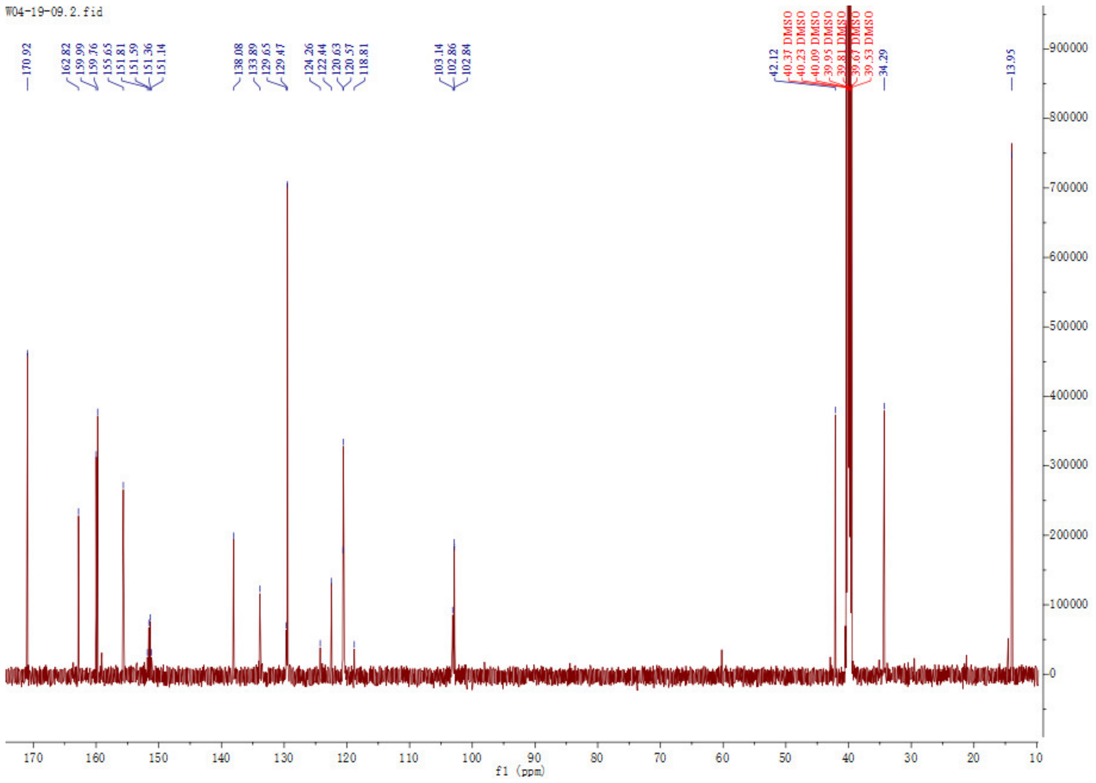

Figure S35. ^{13}C **NMR Spectrum of 6l.**

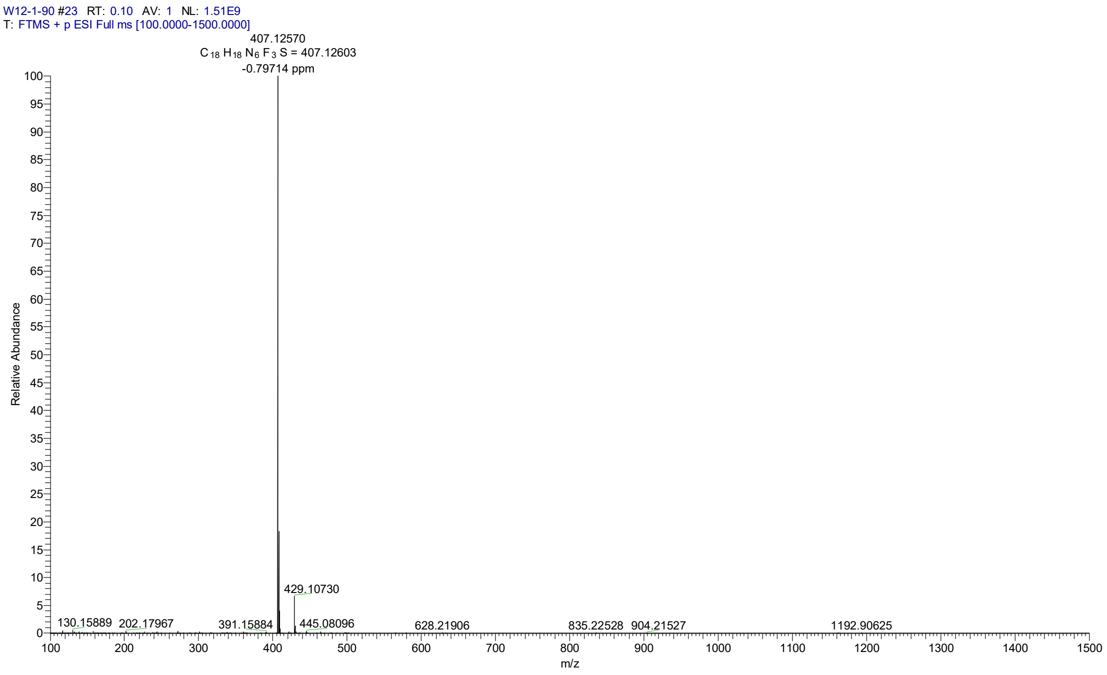

Figure S36. HRMS Spectrum of 6l.

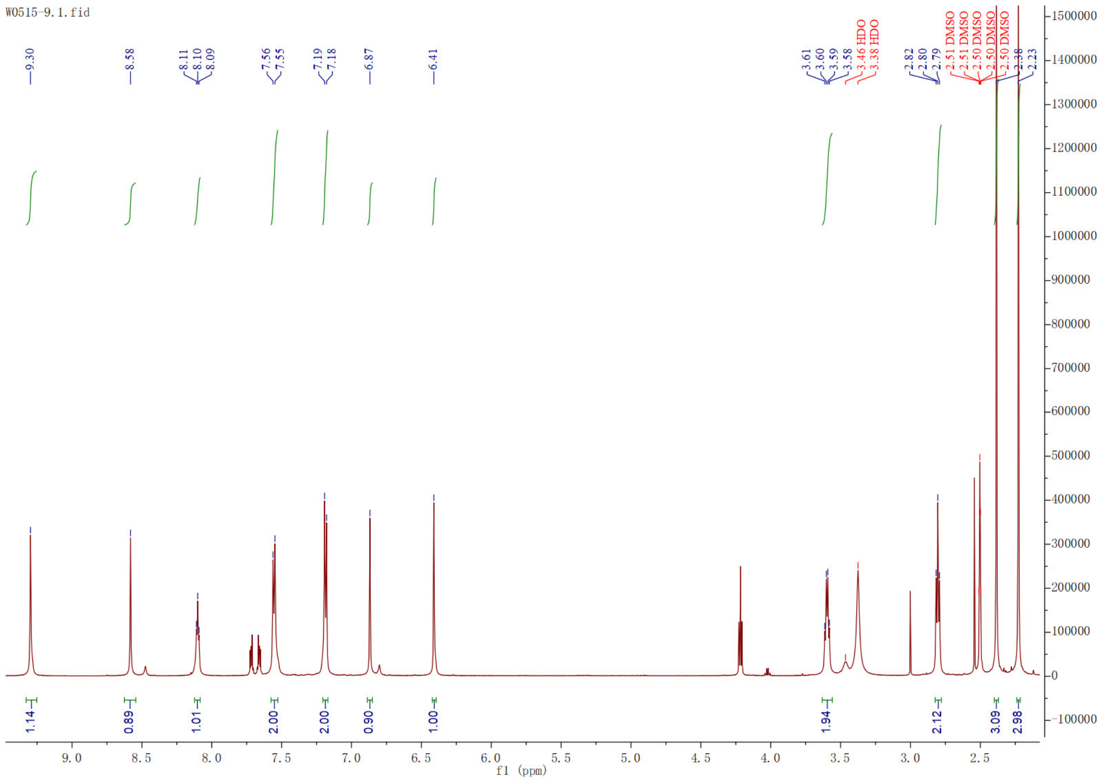

Figure S37. ^{1}H NMR Spectrum of 6m.

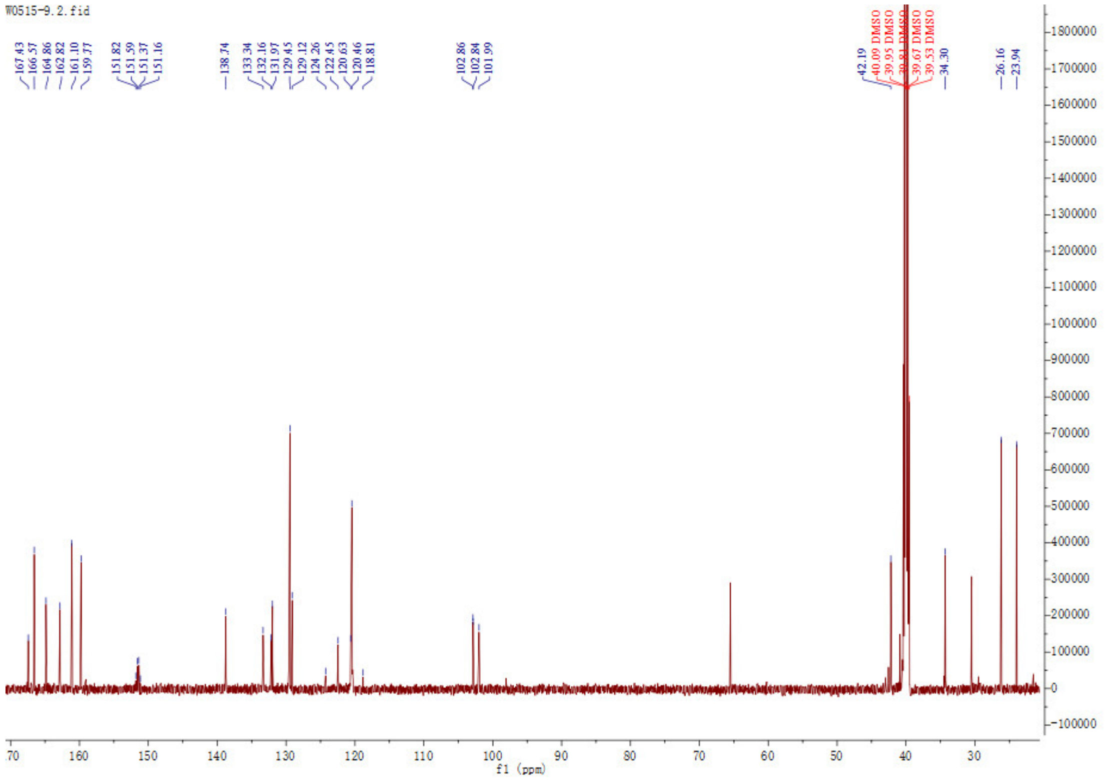

Figure S38. ^{13}C NMR Spectrum of 6m.

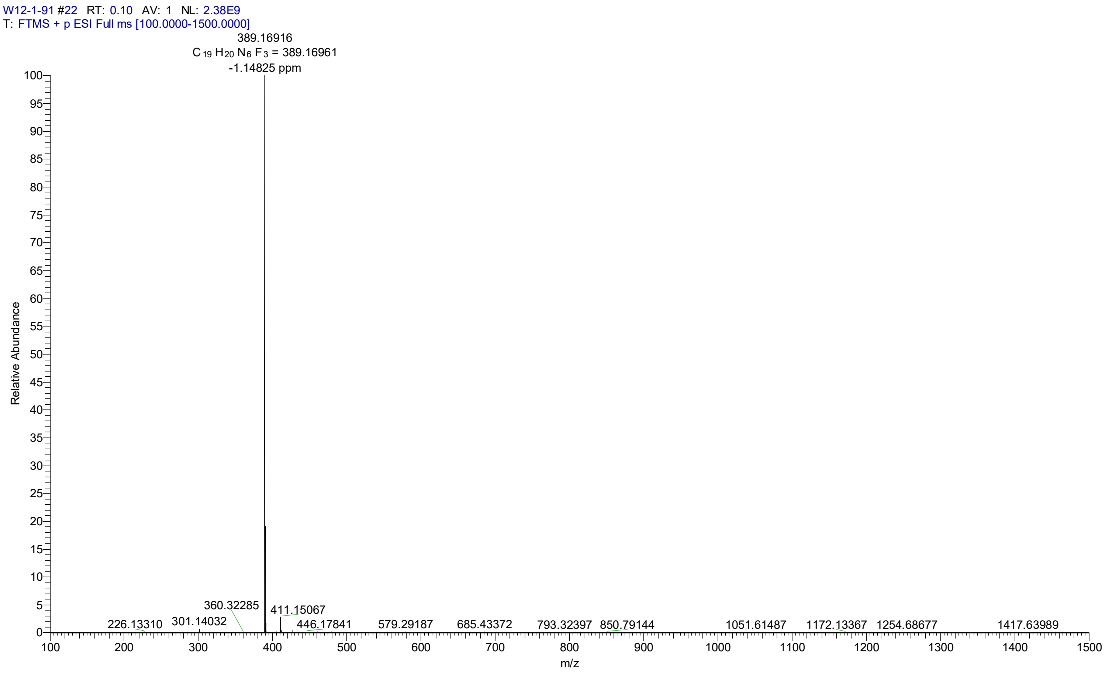

Figure S39. HRMS Spectrum of 6m.

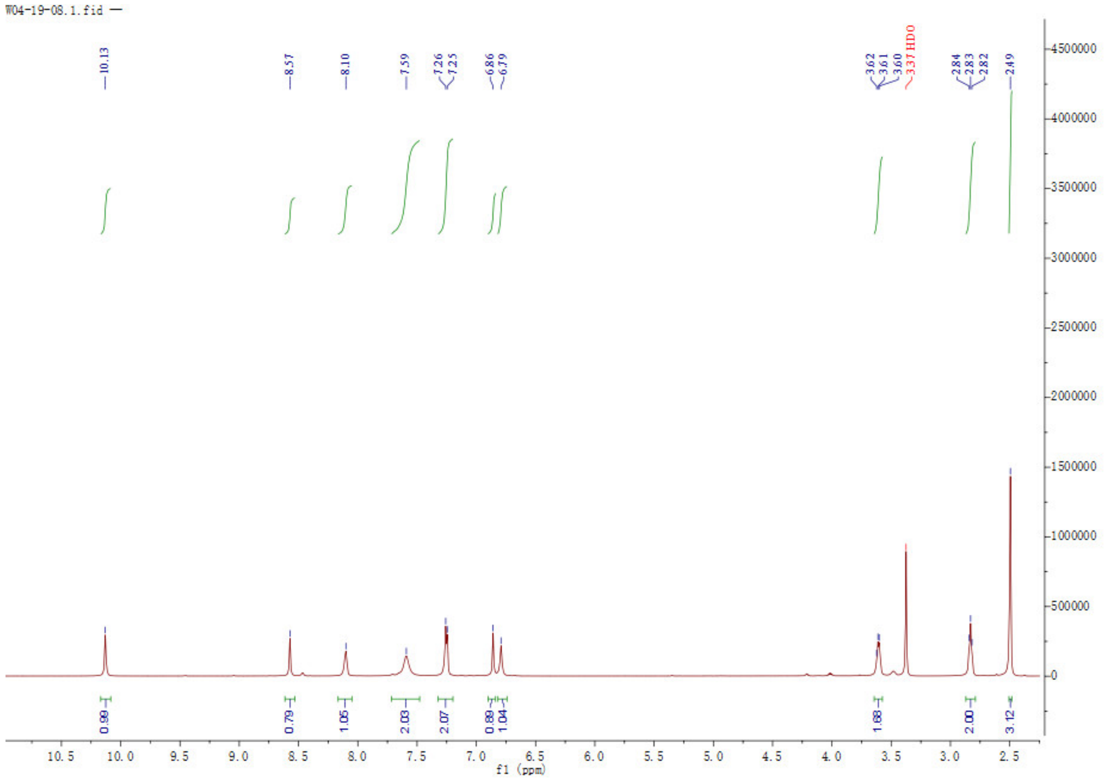

Figure S40. ^{1}H NMR Spectrum of 6n.

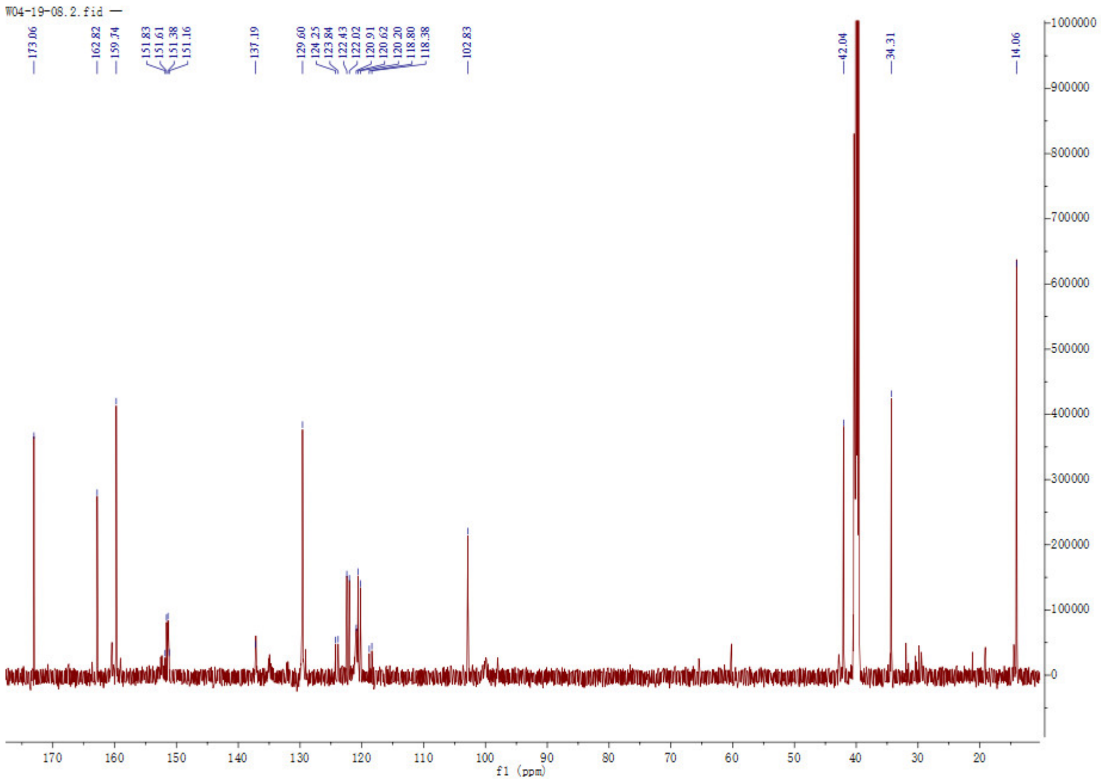

Figure S41. ^{13}C NMR Spectrum of 6n.

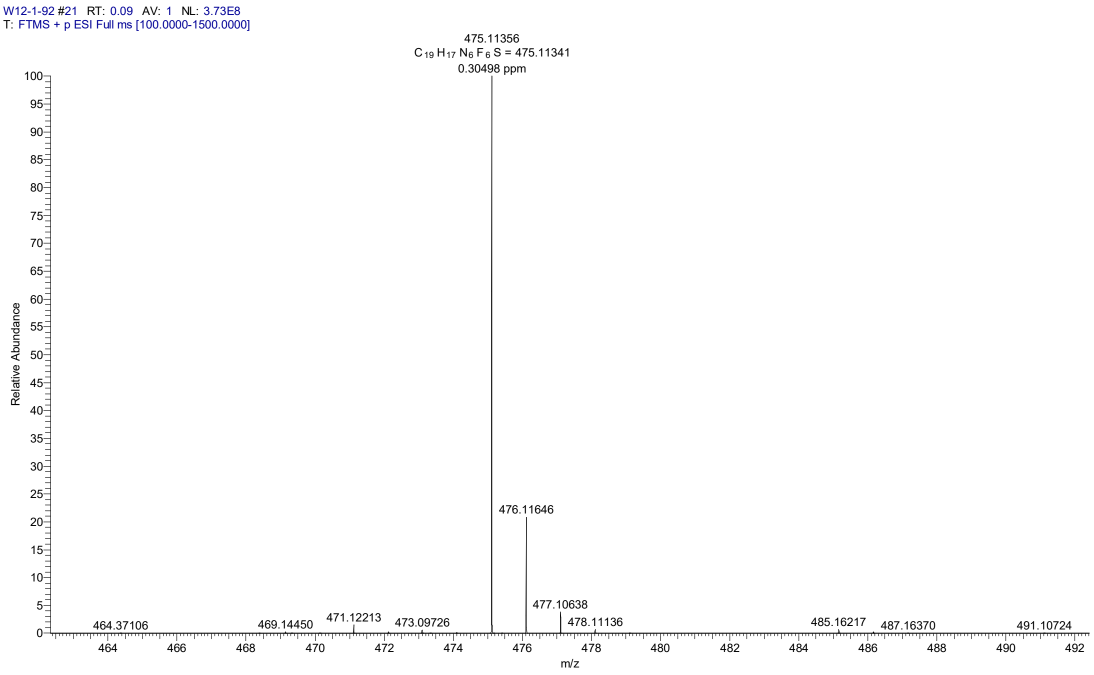

Figure S42. HRMS Spectrum of 6n.

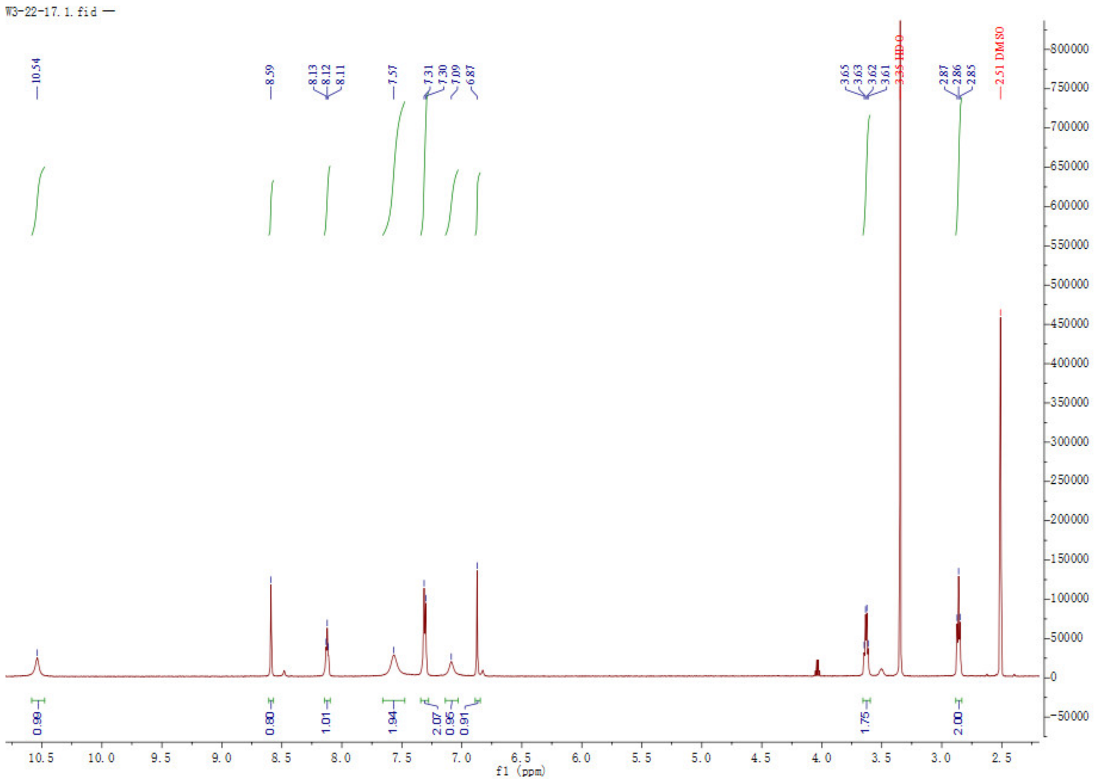

Figure S43. ^{1}H NMR Spectrum of 6o.

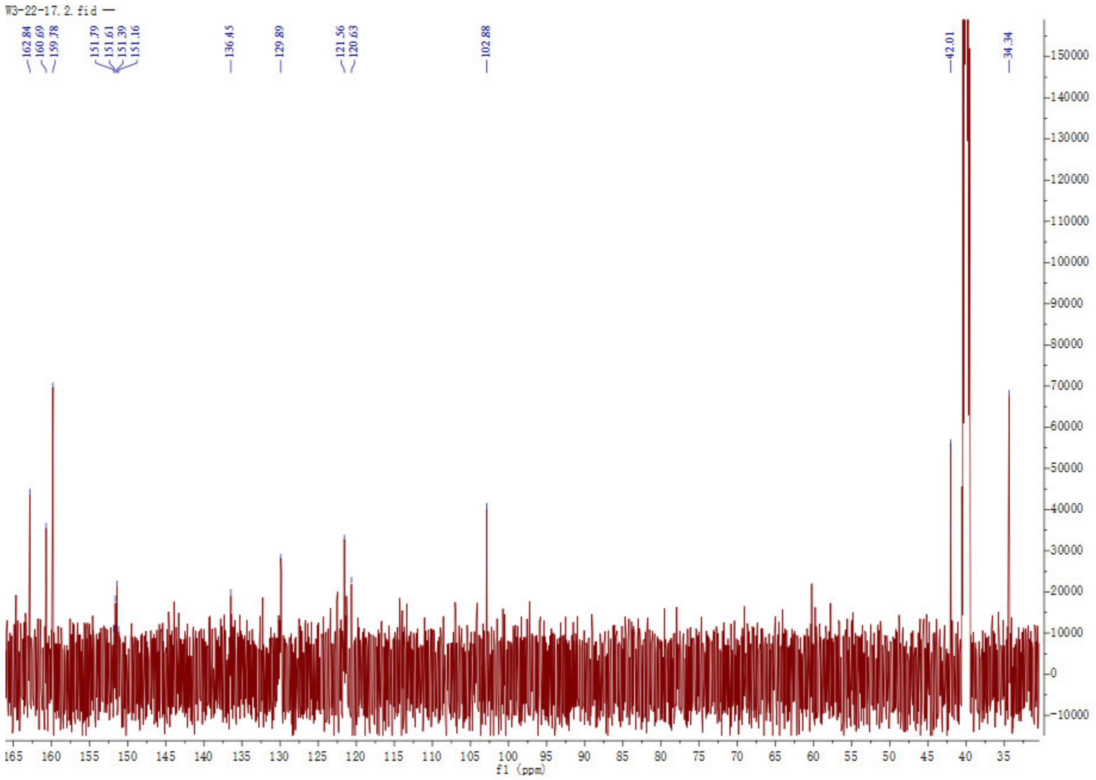

Figure S44. ^{13}C NMR Spectrum of 6o.

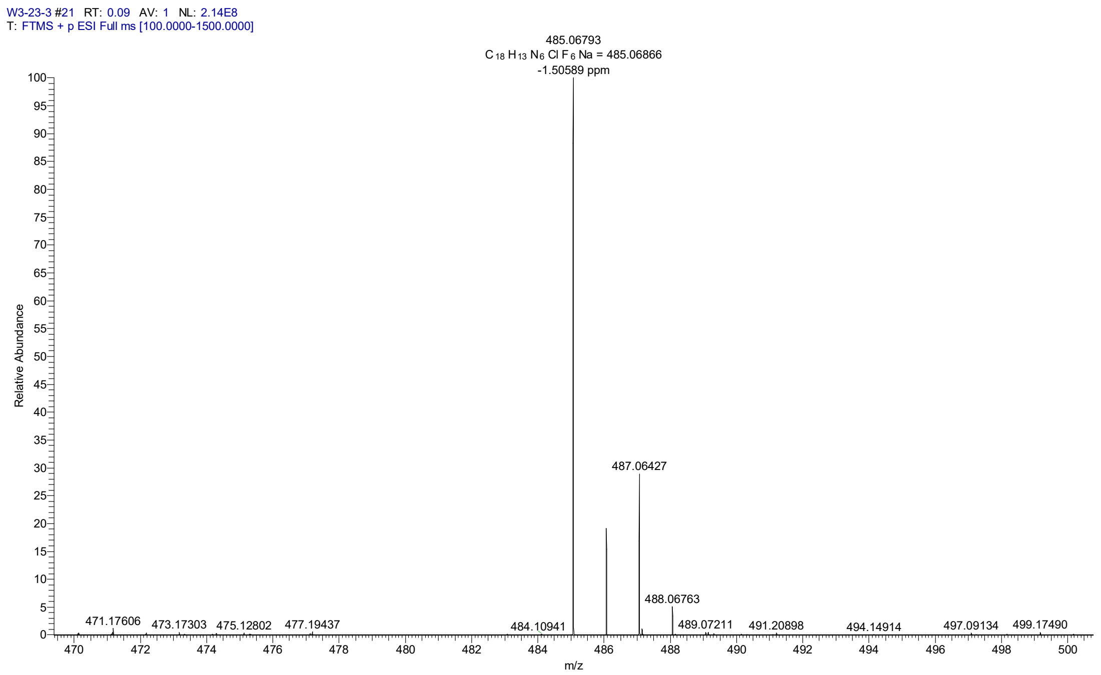

Figure S45. HRMS Spectrum of 6o.

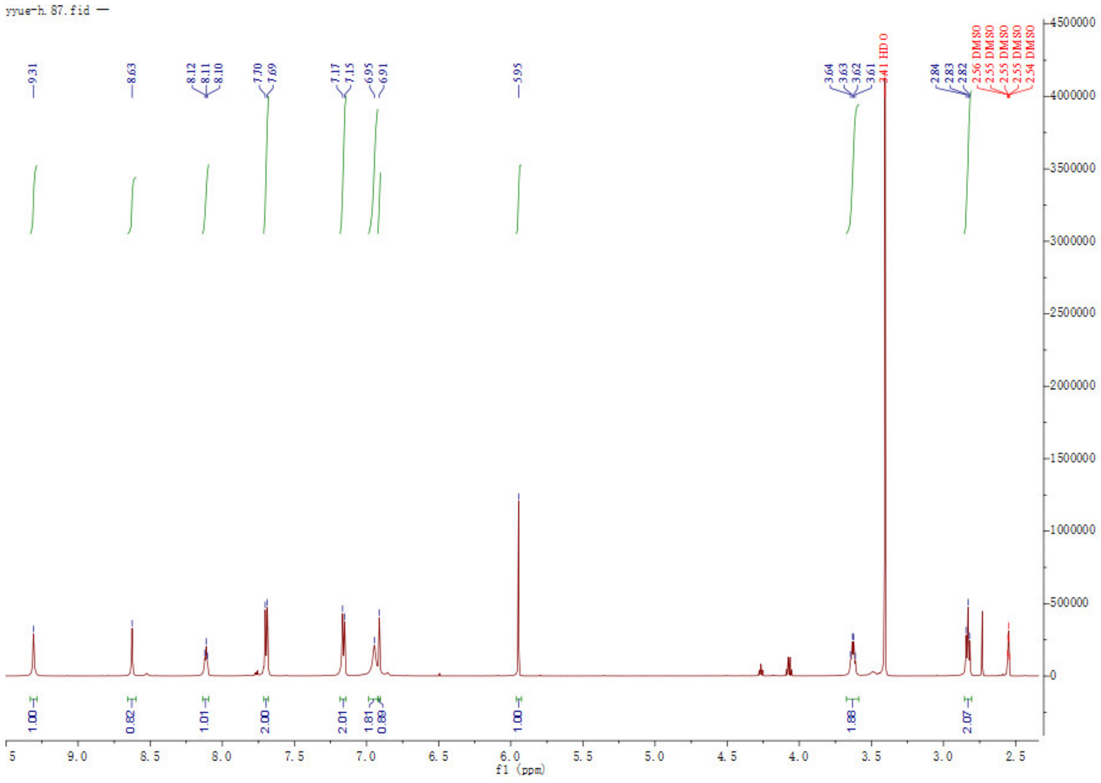

Figure S46. ^{1}H NMR Spectrum of 6p.

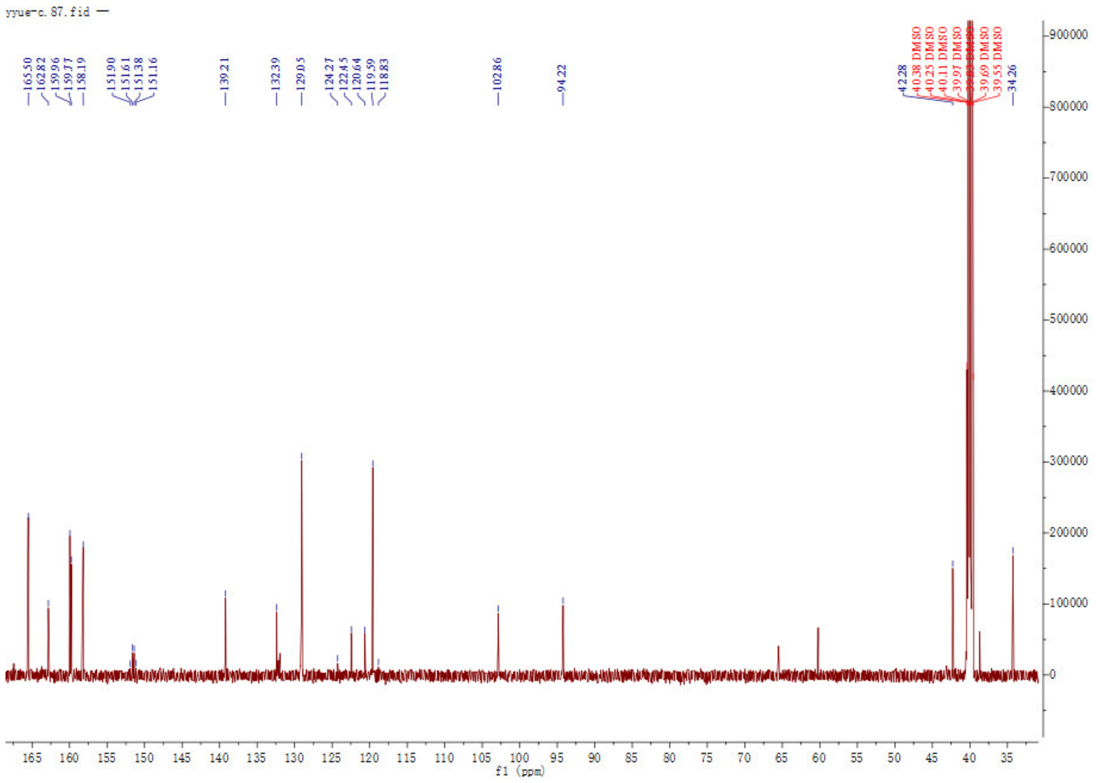

Figure S47. ^{13}C NMR Spectrum of 6p.

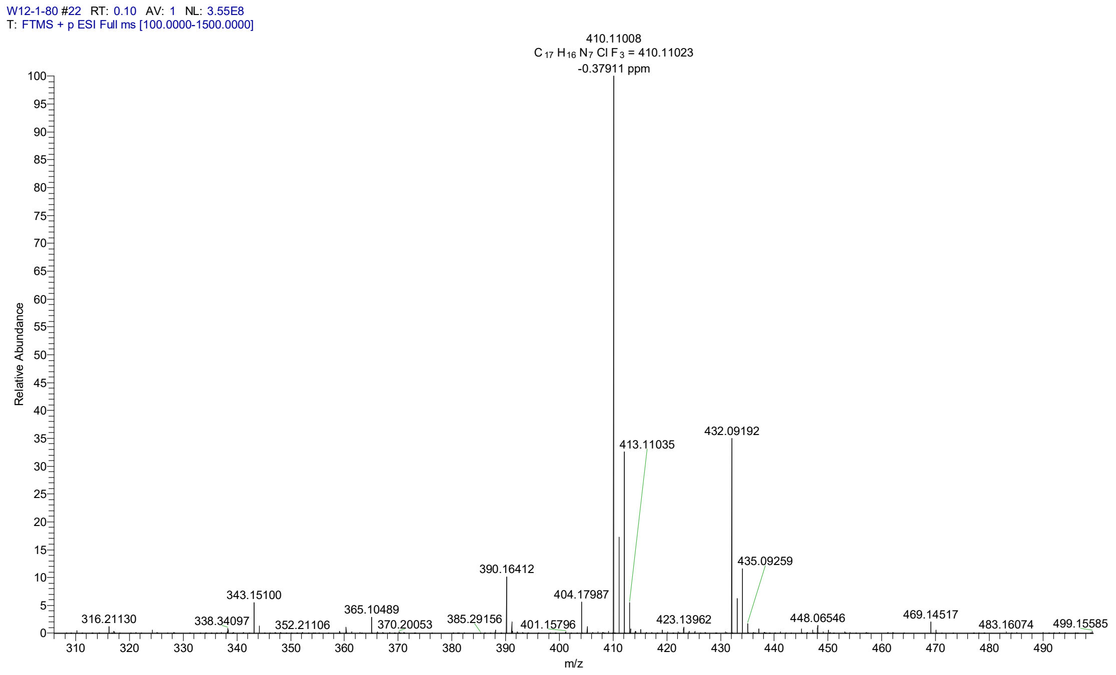

Figure S48. HRMS Spectrum of 6p.

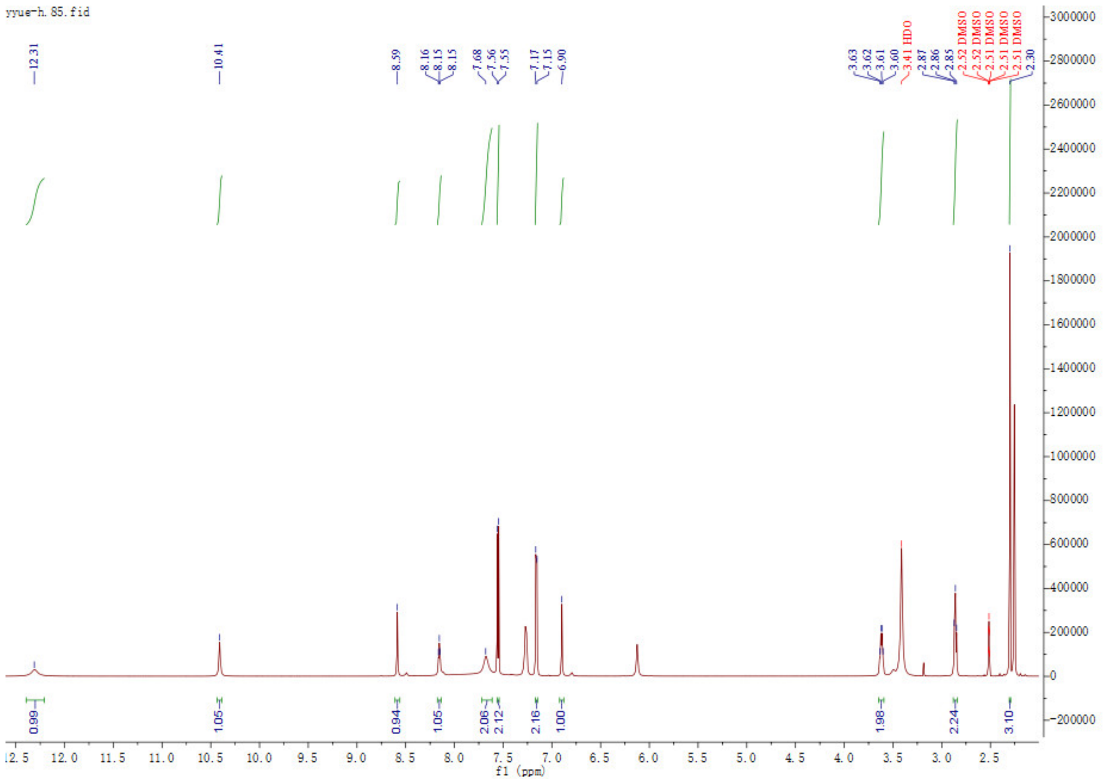

Figure S49. ^{1}H NMR Spectrum of 6q.

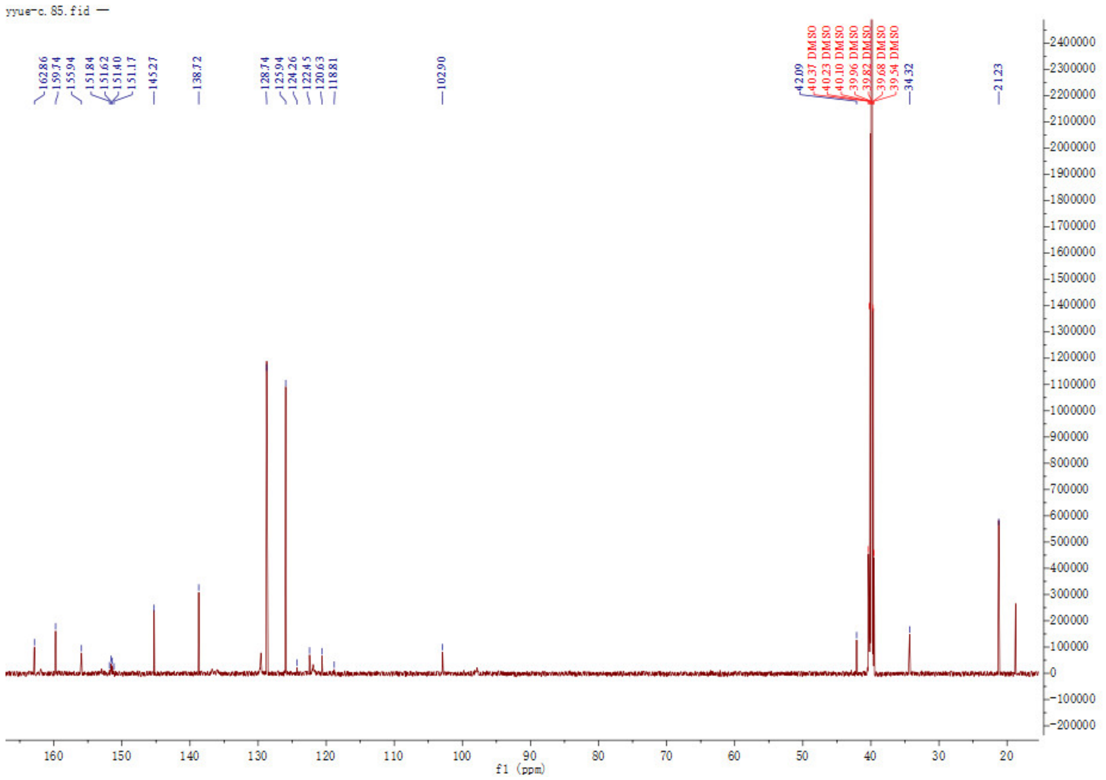

Figure S50. ^{13}C NMR Spectrum of 6q.

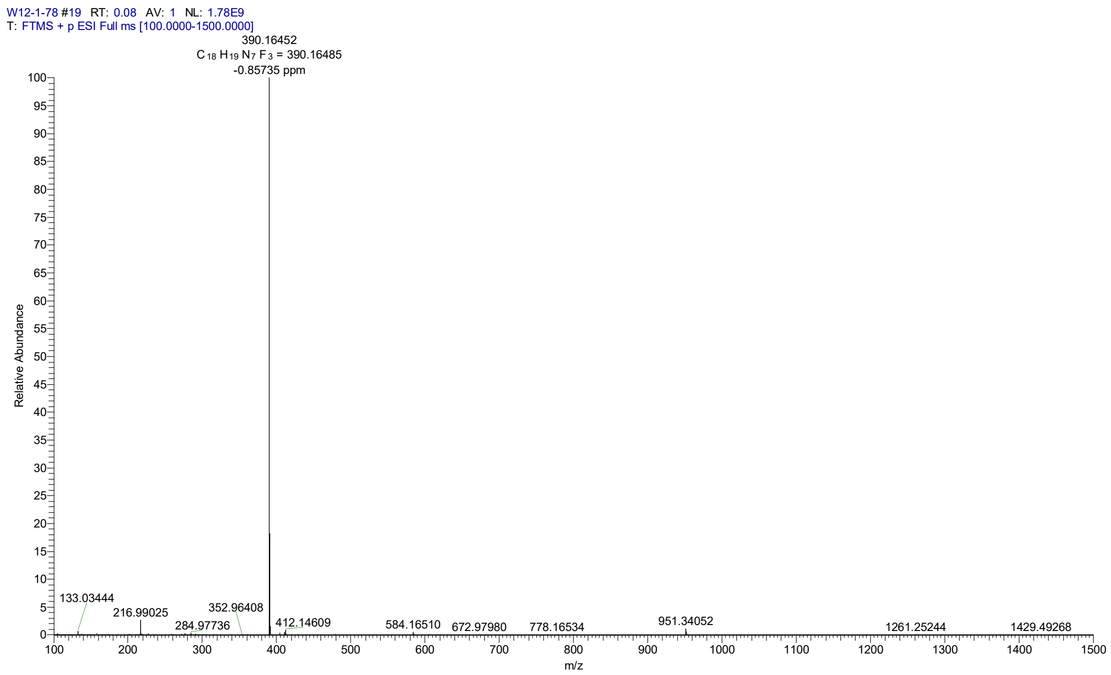

Figure S51. HRMS Spectrum of 6q.

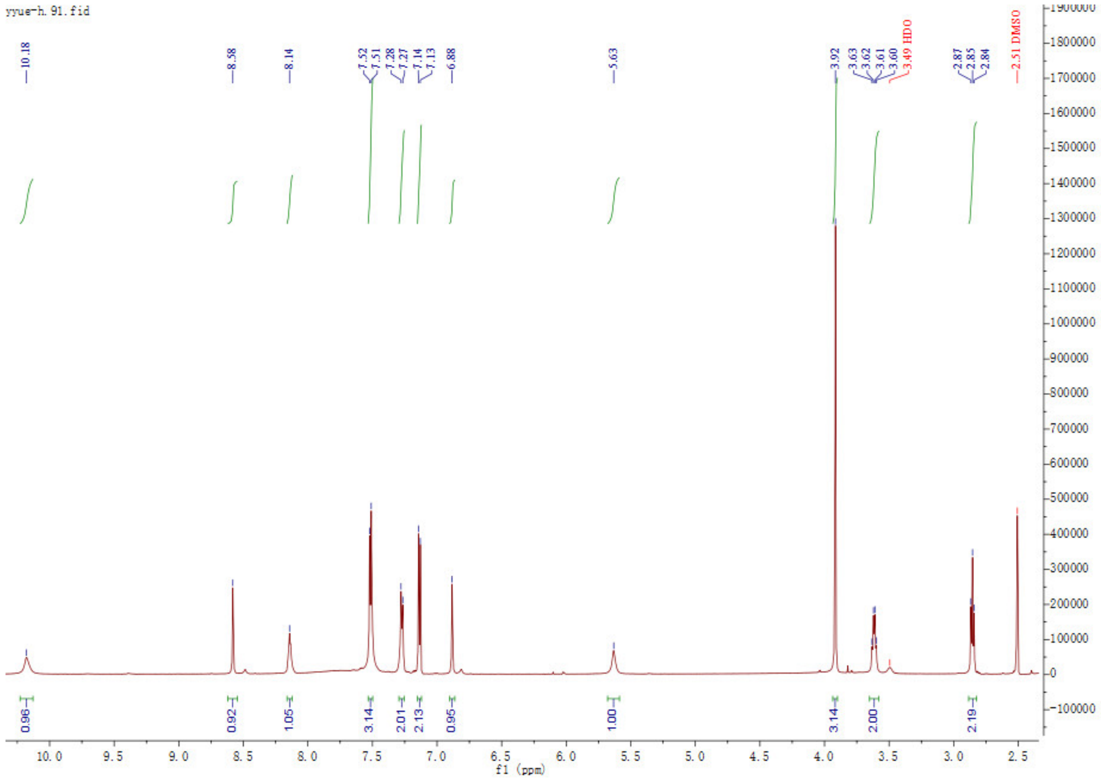

Figure S52. ^{1}H NMR Spectrum of 6r.

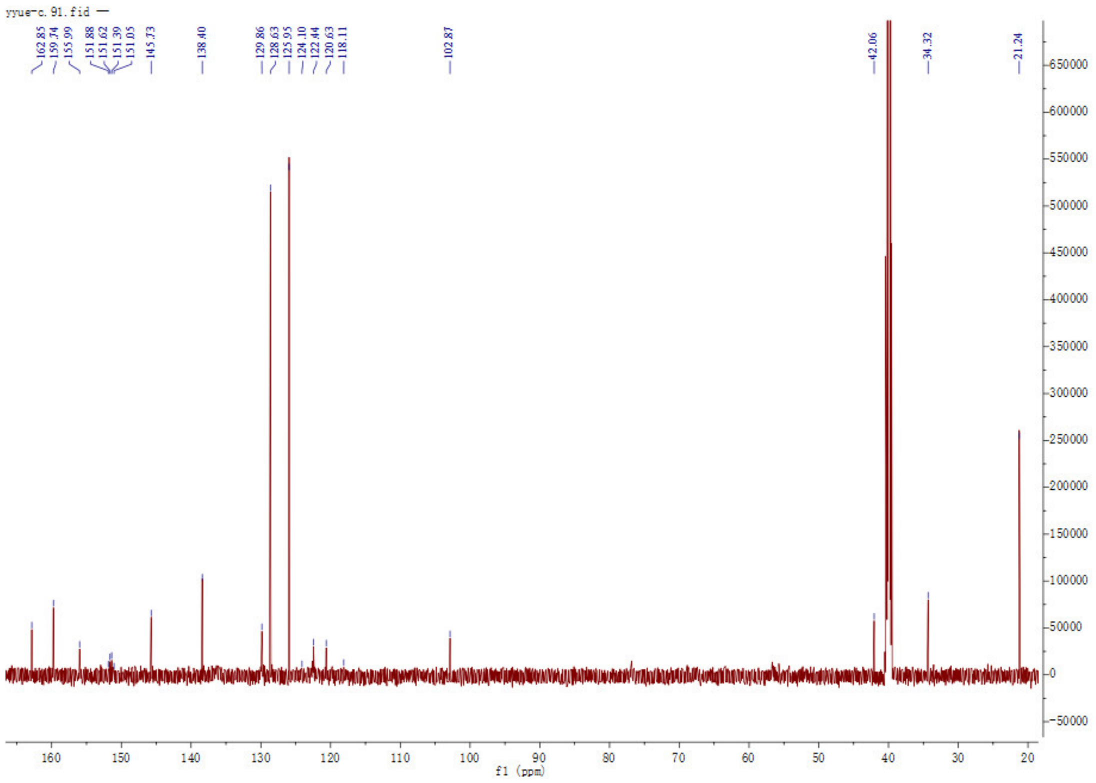

Figure S53. ^{13}C NMR Spectrum of 6r.

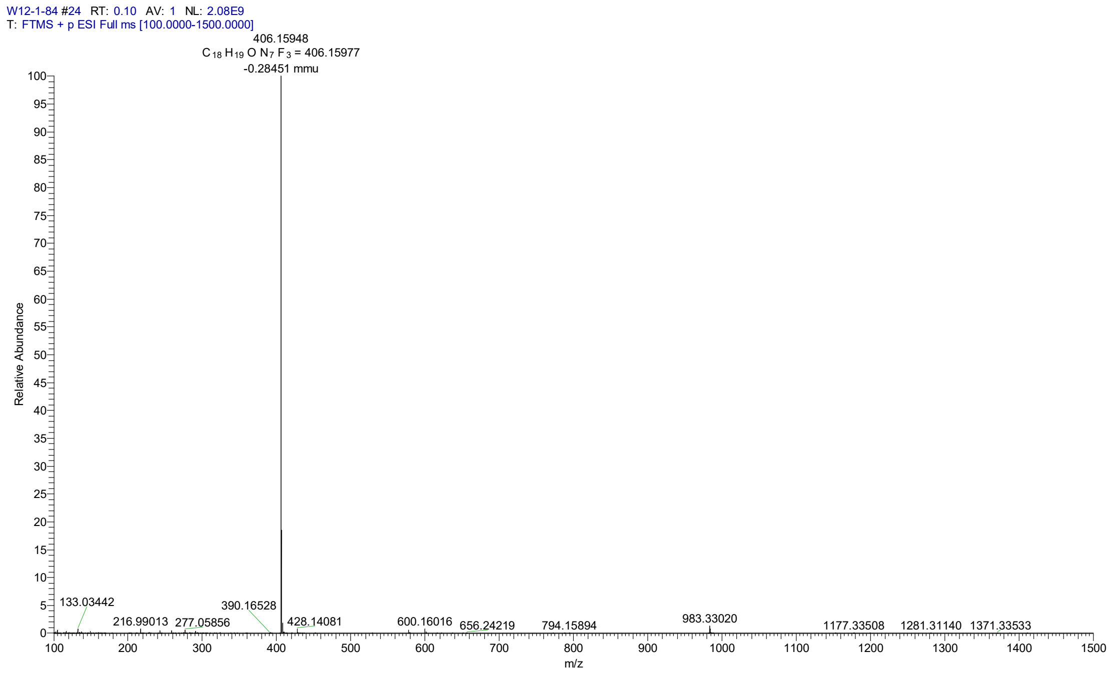

Figure S54. HRMS Spectrum of 6r.

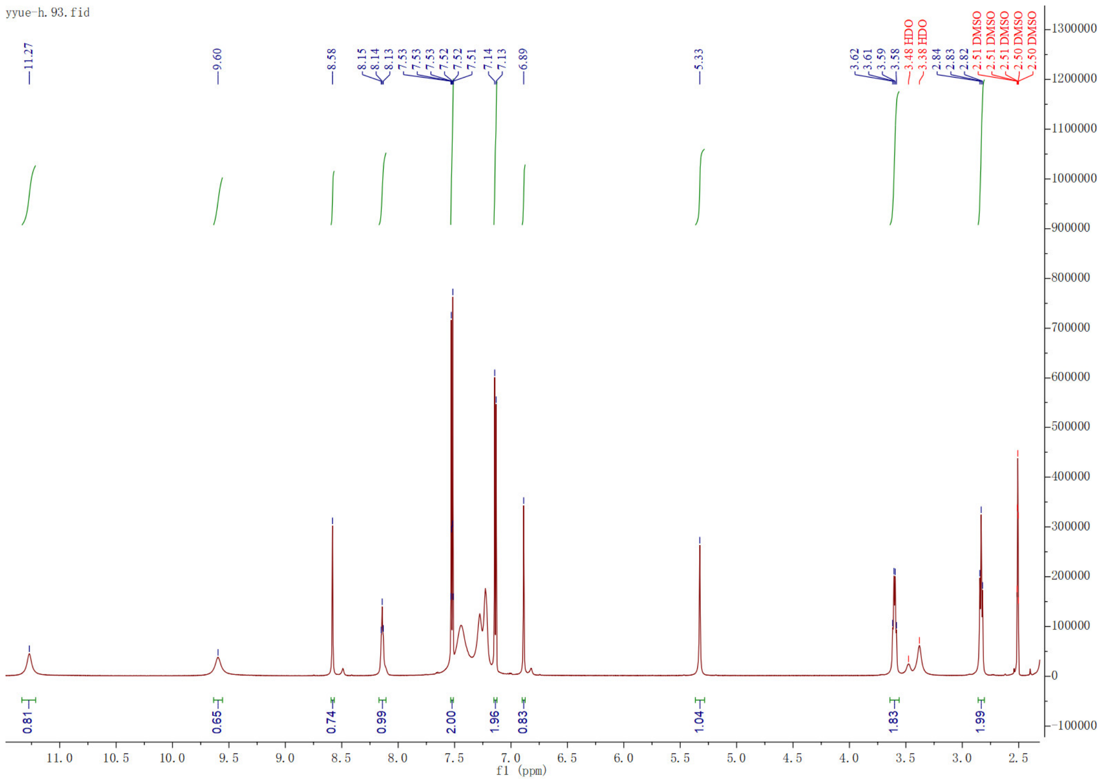

Figure S55. ^{1}H NMR Spectrum of 6s.

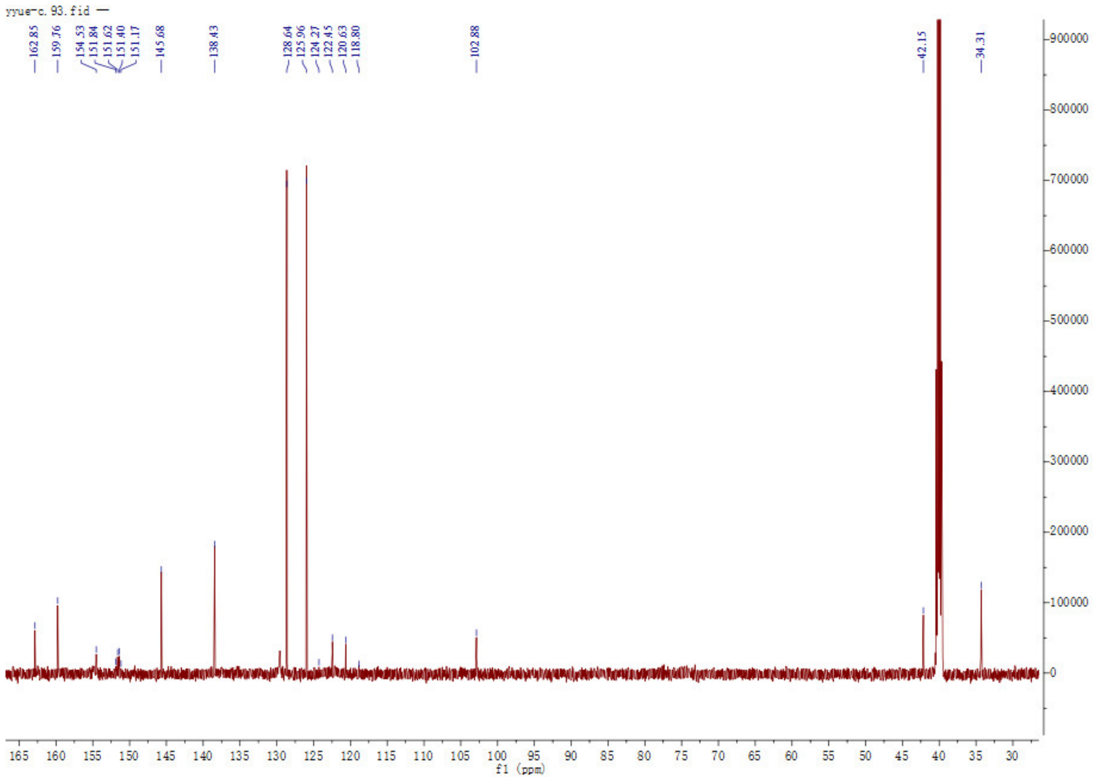

Figure S56. ^{13}C NMR Spectrum of 6s.

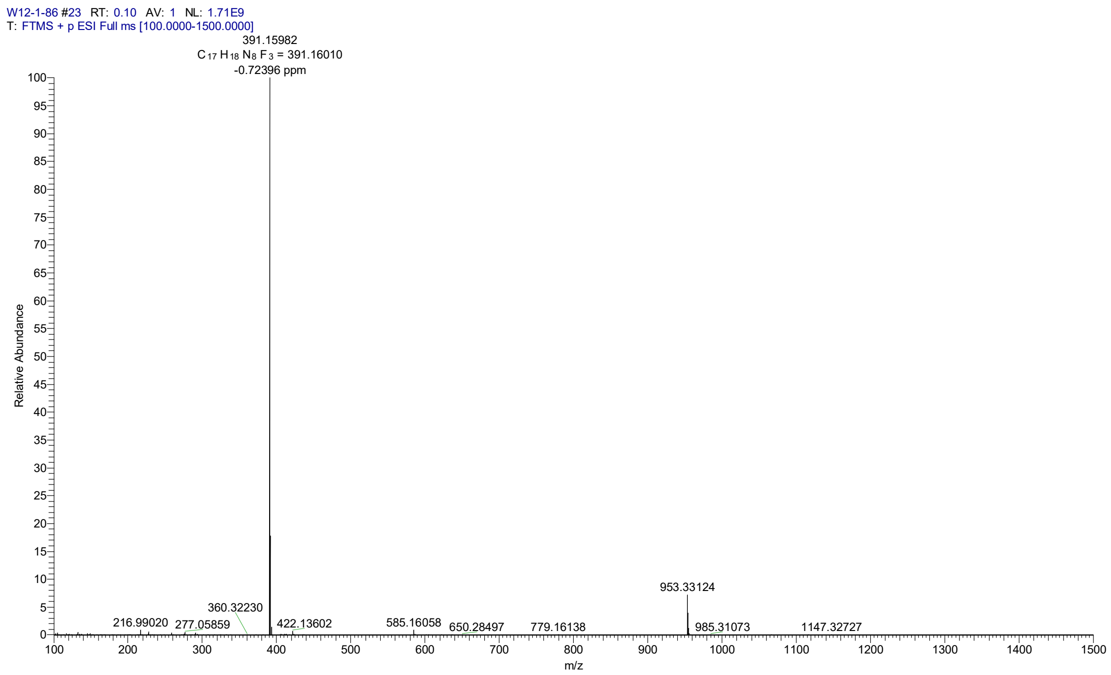

Figure S57. HRMS Spectrum of 6s.

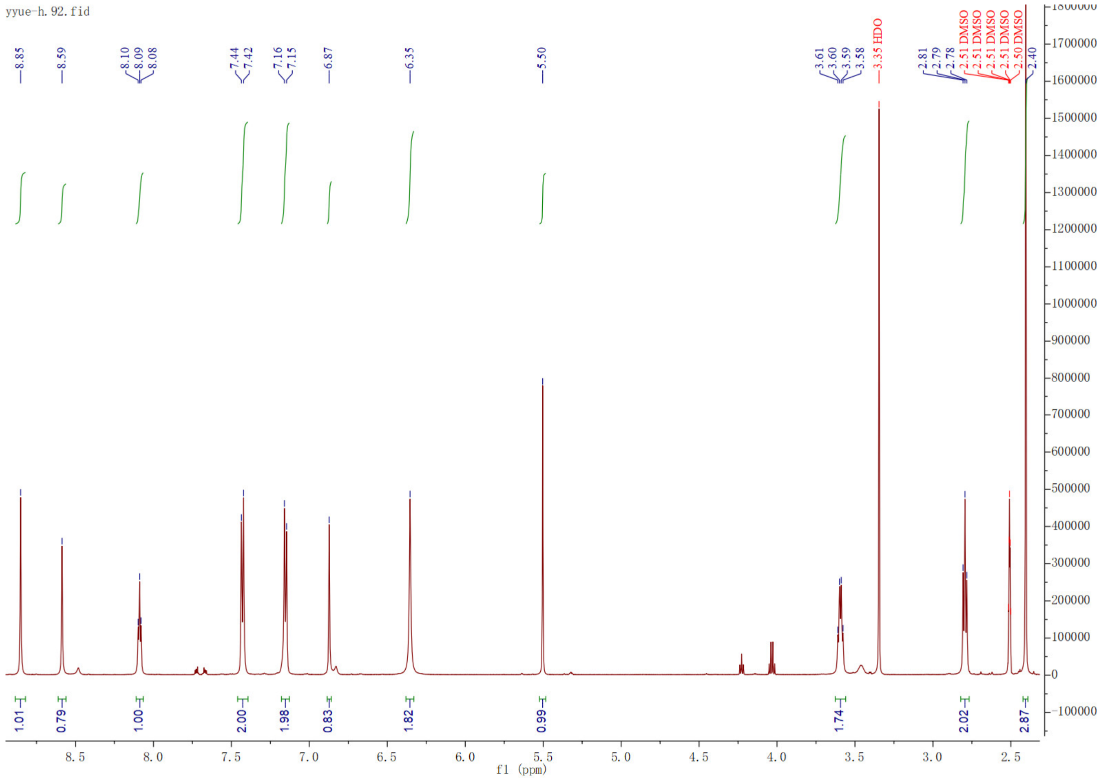

Figure S58. ^{1}H NMR Spectrum of 6t.

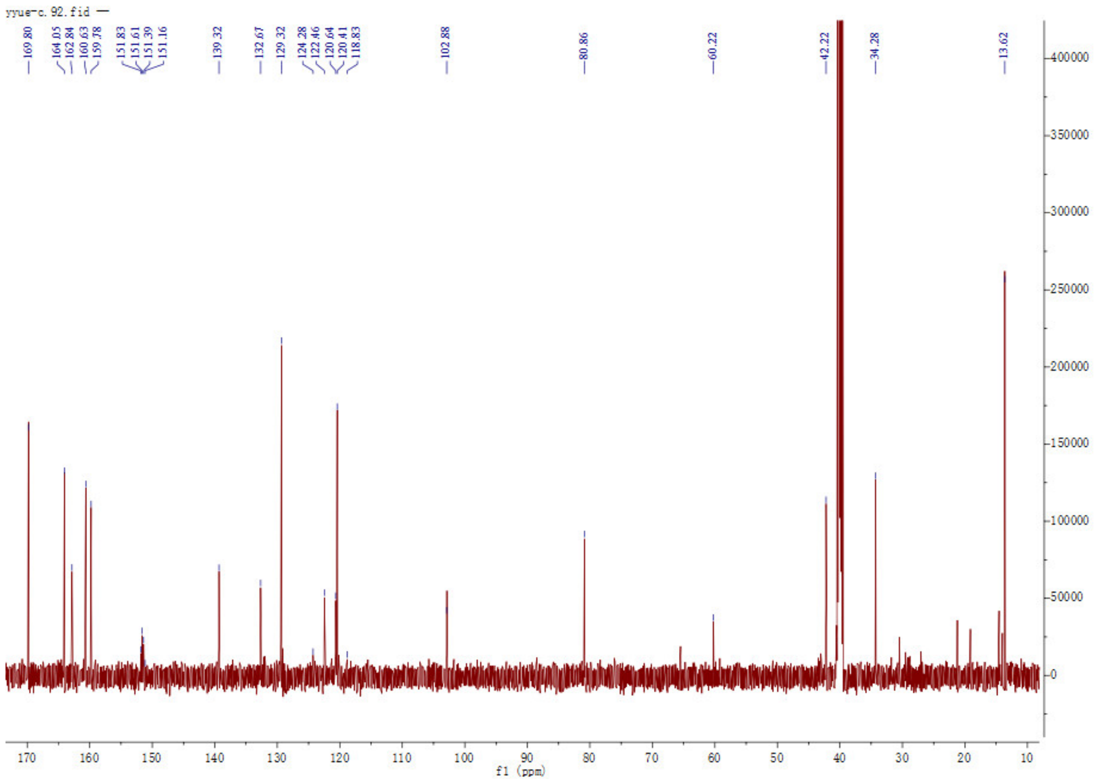

Figure S59. ^{13}C NMR Spectrum of 6t.

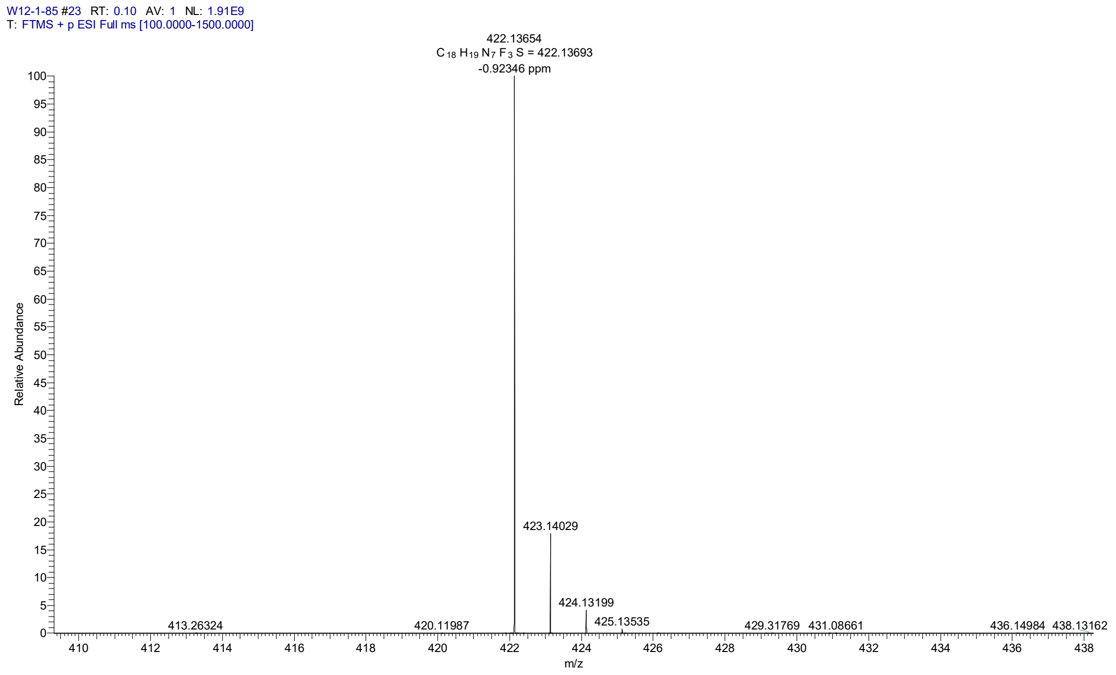

Figure S60. HRMS Spectrum of 6t.
